# Proximity labelling identifies pro-migratory endocytic recycling cargo and machinery of the Rab4 and Rab11 families

**DOI:** 10.1242/jcs.260468

**Published:** 2023-06-23

**Authors:** Beverley Wilson, Chloe Flett, Jakub Gemperle, Craig Lawless, Matthew Hartshorn, Eleanor Hinde, Tess Harrison, Megan Chastney, Sarah Taylor, Jennifer Allen, Jim C. Norman, Thomas Zacharchenko, Patrick T. Caswell

**Affiliations:** ^1^ Wellcome Trust Centre for Cell-Matrix Research, School of Biological Sciences, Faculty of Biology Medicine and Health, Manchester Academic Health Science Centre, The University of Manchester, Manchester, M13 9PT, UK; ^2^ Cancer Research UK Beatson Institute, Glasgow, G61 1BD, UK; ^3^ School of Cancer Sciences, University of Glasgow, Glasgow, G61 1QH, UK

**Keywords:** Endocytic recycling, Cell migration, Rab4, Rab11, Rab25

## Abstract

Endocytic recycling controls the return of internalised cargoes to the plasma membrane to coordinate their positioning, availability and downstream signalling. The Rab4 and Rab11 small GTPase families regulate distinct recycling routes, broadly classified as fast recycling from early endosomes (Rab4) and slow recycling from perinuclear recycling endosomes (Rab11), and both routes handle a broad range of overlapping cargoes to regulate cell behaviour. We adopted a proximity labelling approach, BioID, to identify and compare the protein complexes recruited by Rab4a, Rab11a and Rab25 (a Rab11 family member implicated in cancer aggressiveness), revealing statistically robust protein–protein interaction networks of both new and well-characterised cargoes and trafficking machinery in migratory cancer cells. Gene ontological analysis of these interconnected networks revealed that these endocytic recycling pathways are intrinsically connected to cell motility and cell adhesion. Using a knock-sideways relocalisation approach, we were further able to confirm novel links between Rab11, Rab25 and the ESCPE-1 and retromer multiprotein sorting complexes, and identify new endocytic recycling machinery associated with Rab4, Rab11 and Rab25 that regulates cancer cell migration in the 3D matrix.

## Teaser

**Highlighted Article:** Endocytic recycling pathways control cellular processes including cell migration. Here, we identify protein cargoes and trafficking machinery associated with the major recycling regulators Rab4a, Rab11a and Rab25.

## INTRODUCTION

Endocytosis, trafficking through the endocytic system and recycling back to the cell surface are evolutionarily conserved processes that control the composition of the plasma membrane, which forms the interface between cells and their microenvironment (Simonetti and Cullen, 2019). Endocytic trafficking regulates the positioning and availability of cell surface receptors, and their ability to switch on downstream signalling modules and mediate cell–cell and cell–matrix interactions (Scita and Di Fiore, 2010). Trafficking processes are, therefore, key to determining cell behaviour in normal physiology and in the progression of disease.

Endocytic recycling plays key roles across a myriad of cell functions, including nutrient uptake, ion homeostasis, cell plasticity, division, metabolism, polarity and migration, in a broad range of model organisms and systems. The Rab and Arf families of small GTPases play critical regulatory roles in endocytosis and transit of cargoes through the endolysosomal system (Stenmark, 2009). Rab GTPases act to coordinate trafficking and maintain the fidelity of interactions by recruiting effector proteins that control vesicle and/or endosome linkage to motor proteins, lipid composition, tethering and fusion with target membranes (Stenmark, 2009). Rab GTPases interact with Rab guanine dissociation inhibitors (GDIs) or Rab escort protein (REP) in the cytoplasm – REP facilitates geranylgeranylation at the C-terminus to allow interactions with membranes. Like other GTPases, Rabs cycle through active GTP-bound and inactive GDP-bound states regulated by guanine nucleotide exchange factors (GEFs) and GTPase-activating proteins (GAPs), respectively (Stenmark, 2009). Perhaps the best-characterised recycling regulators include members of the Rab4 (Rab4a and Rab4b) and Rab11 (Rab11a, Rab11b and Rab25) families, where Rab4 proteins control recycling from early endosomes (EEs) and Rab11 proteins control recycling from recycling endosomes (REs) (Grant and Donaldson, 2009).

Detailed molecular mechanisms coordinated by multiprotein complexes have been revealed to control sorting and fusion events in the endolysosomal pathway. Sorting in EEs is controlled by endosomal sorting complexes required for transport (ESCRTs), which recognise ubiquitinylated cargoes destined for degradation, and by specific retrieval pathways that use sorting nexin (SNX)-based recognition of cargo to target them for recycling [retromer, retriever/CCC and ESCPE-1 complexes – each of which depends on the actin regulatory Wiskott Aldrich Syndrome protein and scar homologue (WASH) complex; Simonetti and Cullen, 2019]. Fusion regulators include multi-subunit tethers such as the class C core vacuole/endosome tethering (CORVET) complex (EE:EE fusion), homotypic fusion and vacuole protein sorting (HOPS) complex (late endosomal fusion), class C homologs in endosome–vesicle interaction (CHEVI) complex (transit of cargoes through REs), endosome-associated recycling protein (EARP) (van der Beek et al., 2019; Schindler et al., 2015), and factors for endosome recycling and Rab interactions (FERARI) complex – a Rab11-interacting multiprotein complex that is crucial for Rab11 function (Solinger et al., 2020). Emerging evidence is beginning to describe the links between steps in traffic control. For example, Rab11 is required for the recruitment of the CHEVI complex to REs (van der Beek et al., 2019), and CHEVI (specifically the VPS33B subunit) can interact with the CCC complex (Hunter et al., 2018), which is, in turn, presented cargoes by retriever (McNally et al., 2017), suggesting that there might be handover between these critical regulatory players.

Recycling of receptor tyrosine kinases (including EGFR, c-Met, PDGFR and EphA2), chemokine receptors and adhesion receptors is implicated in eliciting the signalling and cell–matrix interactions that permit motility in 2D and 3D matrices, contributing to development, immune surveillance and the invasiveness of cancer cells (Wilson et al., 2018). The Rab11 family plays a key role in control of cell migration, cancer cell invasion and metastasis (Paul et al., 2015a). Rab25 is upregulated in some cancers and can increase their aggressiveness when the lysosomal protein CLIC3 is co-upregulated to control a pathway that rescues integrins from lysosomes (Dozynkiewicz et al., 2012). The Rab11 effector protein Rab11-family-interacting protein (FIP) 1 [RAB11FIP1; also known as Rab-coupling protein (RCP)] is similarly implicated in cell migration, invasion and cancer progression (Caswell et al., 2008; Muller et al., 2009; Zhang et al., 2009). In both cases, integrins, including α5β1, are key cargoes that drive invasion into the extracellular matrix that contains fibronectin (FN or FN1), the ligand for α5β1. Rab4-driven recycling has been shown to play a key role in directional migration and cancer cell invasion, by controlling the trafficking of cargoes such as αvβ3 integrin and MT1-MMP (Christoforides et al., 2012; Frittoli et al., 2014), particularly in 3D microenvironments low in FN. Although numerous studies have characterised specific GTPases and cargoes that are key to controlling migration and invasion in specific cell contexts, a broad understanding of machineries that control these pro-migratory recycling pathways is lacking.

Here, we used BioID-based proximity labelling to characterise the protein complexes that form around recycling vesicles in a mesenchymal, migratory, ovarian cancer cell line model, specifically focusing on Rab4a, Rab11a and Rab25. Our data reveal a protein–protein interaction (PPI) network that overlaps for these three recycling regulators and links Rab GTPases to previously identified multi-subunit complexes that control trafficking events in the endolysosomal system. In addition, new Rab4a, Rab11a and Rab25-associated proteins were identified that play key roles in the migration of cells in the 3D matrix.

## RESULTS

### Identification of Rab4a, Rab11a and Rab25 interactomes in live cells

**Fig. 1.**
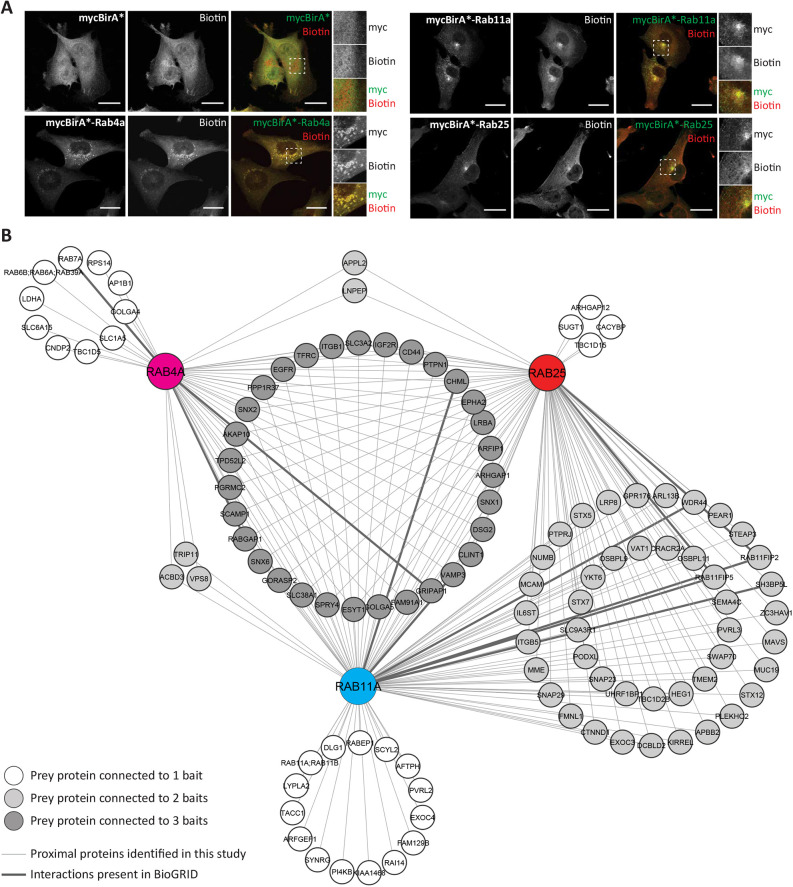
**BioID identifies a network of proteins associated with the regulators of endocytic recycling, Rab4a, Rab11a and Rab25.** (A) Localisation of fusion proteins and biotinylated proteins in A2780 cells stably expressing mycBirA* or mycBirA*-tagged Rab4a, Rab11a or Rab25 cultured with biotin (1 μM, 16 h). Zoomed regions show the perinuclear region of the cell. Images are representative of three independent experiments. Scale bars: 20 μm. (B) Network showing the proteins identified as high-confidence proximal proteins [Bayesian false discovery rate (BFDR)≤0.05] for Rab4A, Rab11a and Rab25. Thicker lines connecting proteins to bait Rab GTPases represent interactions found in the BioGRID PPI database. Coloured nodes represent bait proteins, white nodes represent proteins enriched to one Rab GTPase, pale grey nodes represent proteins enriched to two Rab GTPases and dark grey nodes represent proteins enriched to all three Rab GTPases.

Proximity labelling methods are powerful techniques to understand the formation of protein complexes within living cells. We chose BioID (Roux et al., 2012) as the long labelling time is particularly well suited to long-lived or repeated interactions, such as those that occur during cycles of endocytic recycling at steady state. Because Rab GTPase overexpression can lead to mislocalisation (Gemperle et al., 2022), we adopted a system to select for low expression of mycBirA*–Rab4a, mycBirA*–Rab11a and mycBirA*–Rab25 (alongside a cytoplasmic mycBirA* control), at a level close to the expression levels of the endogenous Rab11 protein (Fig. S1A–C). Rab GTPases localised to endosomal compartments consistent with EEs (Rab4) and REs (concentrated in a perinuclear region for Rab11 and Rab25; Fig. 1A), whereas mycBirA* alone was diffuse in the cytoplasm. Internalisation of transferrin (TFN or TF, the ligand for the recycling transferrin receptor) into EEs for 10 min showed that mycBirA*–Rab4a colocalised with these compartments, whereas a 30 min internalisation period (labelling REs) showed colocalisation with mycBirA*–Rab11a and mycBirA*–Rab25, giving confidence that fusion proteins were not aberrantly distributed (Fig. S1D). Each mycBirA*–Rab efficiently labelled proteins across a range of molecular weights (Fig. S1F), and biotinylated proteins co-distributed with mycBirA*–Rab proteins and were observed at the cell periphery (Fig. 1A). We therefore devised a proteomic approach to compare the protein complexes that form around Rab4a, Rab11a and Rab25 recycling vesicles and endosomes, where biotinylated proteins were isolated from cells expressing mycBirA*–Rab4a, mycBirA*–Rab11a and mycBirA*–Rab25 alongside the mycBirA* control under stringent conditions using an optimised protocol (adapted from Roux et al., 2012), before analysis by tandem mass spectrometry (MS/MS) and label-free quantification (quadruplicate samples; Fig. S1F,G).

Comparison of proteins biotinylated by each mycBirA*–Rab with those biotinylated by the mycBirA* control using robust statistical analysis by Significance Analysis of INTeractome (SAINT) (Teo et al., 2014) identified a high confidence interactome of proteins (Fig. S1E) associated with Rab4a (46 proteins), Rab11a (93 proteins) and Rab25 (81 proteins) that were well distinguished from those associated with the mycBirA* control by principal component analysis (Fig. 1B; Fig. S1H; Table S1). This extended to a ‘longlist’ of 134 proteins associated with Rab4a, 266 with Rab11a and 230 with Rab25 when preys were identified by a fold-change rule (>2-fold enrichment, at least two unique peptides, in at least three of four repeats; Table S2). Of the 31 high-confidence associated proteins shared by all three Rab GTPases, the fold enrichment was closely correlated between the Rab11 family members Rab11a and Rab25, but less so with Rab4a (Fig. S1I). The high-confidence interactomes were significantly enriched for Gene Ontology (GO) biological processes associated with protein localisation and vesicle fusion, although, interestingly, terms related to ‘vesicle docking’ were only enriched to Rab11a and Rab25 (Fig. S2A–C). GO cellular compartments were unsurprisingly dominated by vesicle- and endosome-related terms, and, interestingly, terms related to ‘cell leading edge’, ‘lamellipodium’ and ‘focal adhesion’ were enriched for all GTPases (Fig. S2A). The GO molecular function and biological process terms ‘lamellipodium organisation’ and ‘lamellipodium morphogenesis’ were enriched, suggesting that many of the cargoes are involved in processes related to adhesion and protrusion (Fig. S2B,C). Rab4a-associated proteins were also associated with terms related to ‘amino acid transport’ (Fig. S2B,C), suggesting these metabolic processes could be specifically regulated by this recycling regulator.

### Rab4 is required for focal adhesion and lamellipodia formation, whereas Rab11 and Rab25 promote filopodia formation

**Fig. 2.**
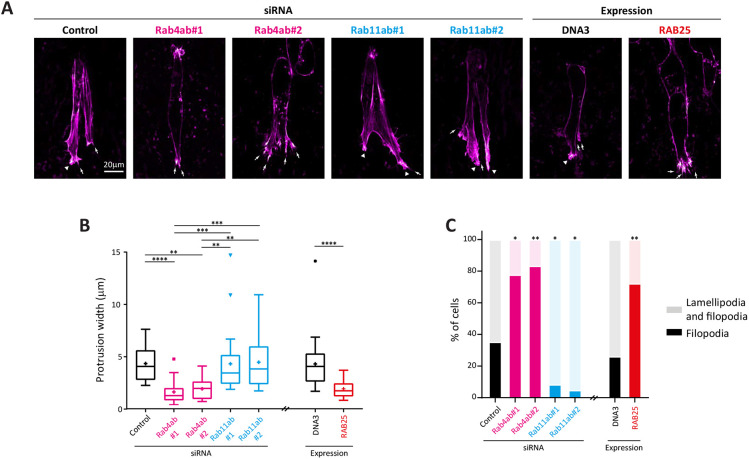
**The Rab4 and Rab11 families differentially regulate F-actin protrusions.** A2780-DNA3 cells and A2780-Rab25 cells [depleted of Rab4a and Rab4b (indicated as Rab4ab), or Rab11a and Rab11b (indicated as Rab11ab) by siRNAs where appropriate] were seeded into the cell-derived matrix (CDM) for 16 h before fixation, staining for F-actin, and imaging by confocal microscopy. (A) Representative images (magenta hot lookup table); arrows indicate filopodia and arrowheads indicate lamellipodia. Scale bar: 20 µm. (B) The width of the protrusion extended furthest from the cell was measured 2–5 µm from its greatest extent. Boxes indicate the 25–75th percentiles, whiskers are drawn in Tukey style where the upper whisker equals the 75th percentile plus 1.5× the interquartile range and the lower whisker equals the 25th percentile minus 1.5× the interquartile range, the mean is marked with a ‘+’, the median is marked with a line and individual points are plotted where they lie outside the whiskers. Statistical analysis was performed with one-way ANOVA and Kruskal–Wallis post hoc test (control versus Rab4ab or Rab11ab) or Mann–Whitney test (DNA3 versus Rab25 cells). (C) For each cell, protrusions were scored as ‘lamellipodial’ or ‘filopodial’. Note that cells with only lamellipodial protrusions were not observed (statistical analysis with Fisher's exact test). *n*>17 cells/condition from at least three independent experiments. *P<0.05; **P<0.01; ***P<0.001; ****P<0.001.

We and others have shown that Rab4 and Rab11 families control integrin recycling and cell migration (Moreno-Layseca et al., 2019; Paul et al., 2015a), and we further demonstrated that Rab11-RCP recycling can be induced to promote formation of filopodia in cells moving in 3D matrices (Jacquemet et al., 2013; Paul et al., 2015b). Interestingly, knockdown of Rab4a and Rab4b led to a decrease in the number of paxillin (PXN)-positive focal adhesions formed by cells in 2D, whereas Rab11 knockdown or Rab25 overexpression had no effect (Fig. S2D,E). Given that GO analysis also pointed towards cytoskeletal regulation, we analysed the morphology of F-actin within protrusions formed by cells moving within the 3D cell-derived matrix (CDM). A2780 cells moving in the 3D CDM extend several protrusions, and these protrusions are often tipped by small lamellipodia, characterised by wider veils of F-actin at the leading edge and/or by narrow finger-like filopodia (Fig. 2A). We therefore measured the width of the most advanced protrusion and scored for the presence of lamellipodia and filopodia-bearing protrusions, or filopodia alone (lamellipodia alone were not observed). Knockdown of Rab4a and Rab4b led to a decrease in protrusion width and an increase in the number of cells bearing protrusions tipped solely by filopodia (Fig. 2A–C). Conversely, knockdown of Rab11a and Rab11b had no effect on protrusion width; however, significantly fewer cells bore filopodial protrusions (Fig. 2A–C). Overexpression of Rab25 (at levels close to those observed in aggressive ovarian cancers; Caswell et al., 2007; Cheng et al., 2004) also significantly reduced the width of protrusions extended from cells moving in the 3D CDM, and most of these protrusions were tipped by filopodia alone (Fig. 2A–C). These data reinforce the notion that Rab4 and Rab11 pathways (and/or the cargoes they carry) are antagonistic, and demonstrate that Rab4 supports the formation of wide lamellipodial protrusions, whereas the Rab11 family promotes filopodial protrusion.

### The trafficking machinery associated with Rab4a, Rab11a and Rab25

**Fig. 3.**
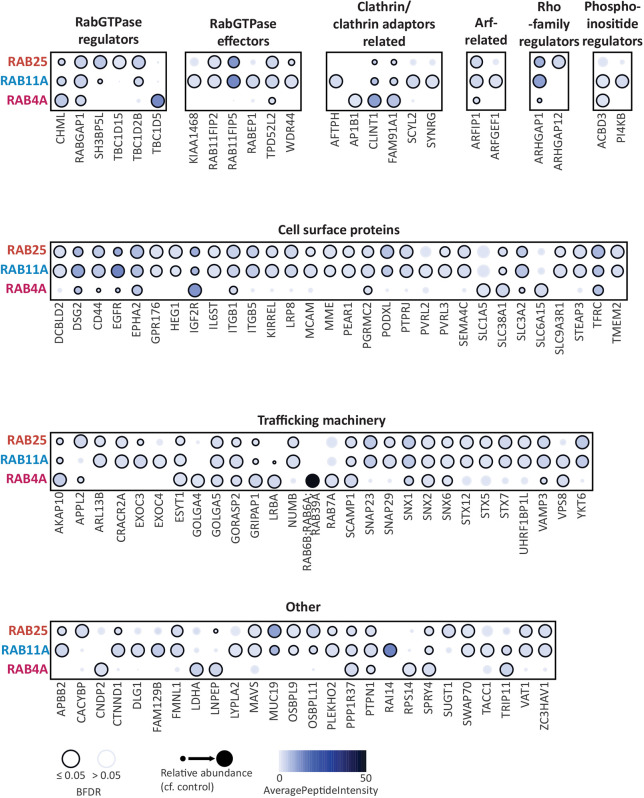
**Enrichment of associated proteins to endocytic recycling Rab GTPase baits.** Dot plots of the high-confidence proximal proteins were generated using the ProHits-viz online tool (Knight et al., 2017). Proximal proteins are represented by circles and are attributed to each of the three bait Rab GTPases in rows and were manually split into different groups based on their function. Circle size relates to the relative abundance of the protein in that sample (analogous to fold change). Circle colour relates to the average intensity (AveragePeptideIntensity) of the protein in that sample. Circle outline relates to whether the protein is classed as a high-confidence enriched protein to that sample (BFDR≤0.05).

The Rab-associated proteins within our robust proteomic dataset were predominantly a range of cell surface proteins, trafficking machinery, Rab/Arf/Rho regulators and effectors, phosphoinositide regulators and clathrin-associated proteins (Fig. 3). These included many known Rab4a, Rab11a and Rab25 interactors (categorised based on function; Fig. 3), for example, CHM-like Rab escort protein (CHML, also known as Rab escort protein 2) was robustly recruited by all three Rab GTPases (Fig. 3), but it also revealed specific interactions and previously uncharacterised links to trafficking regulators.

#### Identification of known Rab effectors

Of the well-characterised Rab11 interactors, Rab11FIP5, Rab11FIP2, WDR44, KIAA1468 (RELCH), EXOC3, OSBPL9 and OSBPL11 were robustly recruited to both Rab11 and Rab25 (Fig. 3; Tables S1 and S2) and Rab11FIP1 (longlists only) was also enriched. Interestingly, PI4KB was significantly enriched to Rab11a only, suggesting that although many effectors are shared, PI4KB is relatively specific. The intracellular nanovesicle protein TPD52L2 (TPD54) (Larocque et al., 2020) was recruited to each Rab, but was the most abundant with Rab11 and Rab25. Rab4a recruited its known effectors GRIPAP1 (GRASP1) and RUFY1 (longlist only), although, interestingly, the Rab effector RABEP1 (Rabaptin-5) was a high-confidence Rab11a-binding partner that appeared on the lower-confidence Rab4 longlist (Fig. 3; Tables S1 and S2). Interestingly, recruitment of other Rab GTPases was suggested by the BioID dataset. Surprisingly, Rab11a was not enriched to Rab25, despite their high sequence similarity. Rab4a strongly recruited Rab6 (referring to Rab6A, Rab6B and Rab39A; a protein group not distinguishable by MS/MS) and Rab7a, suggesting interplay between these trafficking routes (Fig. 3). Furthermore, Rab11a and Rab25 recruited the related GTPases Arl13b (Fig. 3), RABL6 (longlist only, Table S2) and CRACR2A (Rab46), a dynein adaptor that can play a role in the stimulated release of Weibel–Palade bodies in endothelial cells (Miteva et al., 2019; Pedicini et al., 2021; Wang et al., 2019). Taken together, these data give confidence that BioID is an excellent technique to capture and compare Rab interactions in live cells.

#### GEFs

Although GEFs for Rab11 have been identified, those for Rab4 have remained elusive. The TRAPP II complex activates Rab11 homologues in yeast and *Drosophila* (Lamber et al., 2019; Sakaguchi et al., 2015), but components of this complex were not enriched in our dataset. Recently, SH3BP5 (REI-1) was identified as a mammalian Rab11 GEF and, subsequently, SH3BP5 and the closely related SH3BP5L were shown to behave as GEFs for Rab11 and Rab25 *in vitro* (Jenkins et al., 2018; Sakaguchi et al., 2015). Our data show that SH3BP5L was enriched to both Rab11a and Rab25, albeit with higher levels associated with Rab25 (Fig. 3; Tables S1 and S2). DENND4C, related to the *Drosophila* Rab11 GEF CRAG and reported to have high activity towards Rab10 in mammalian cells (Xiong et al., 2012; Yoshimura et al., 2010), was enriched to Rab11a (longlist only, Table S2). Rab11 could therefore potentially act upstream of Rab10 in a Rab GTPase cascade, or, given that Rab10 was not identified in our dataset and analysis of DENND4C activity towards Rab11 has not been published, it is possible that DENND4C could act as an alternate GEF for Rab11. Furthermore, SMCR8 (part of the C9orf72–SMCR8 complex with GEF activity for Rab8 and Rab39) was enriched to Rab25 (longlist only, Table S2), which suggests that it has GEF activity specific to this GTPase. However, no potential Rab4 GEFs were identified.

#### GAPs

GAPs for the Rab4 and Rab11 families are better understood and, of these, the Tre-2/Cdc16/Bub2 (TBC) domain family are best characterised. RabGAP1 (TBC1D11; a Rab2, Rab4, Rab6, Rab11 and Rab36 GAP) was enriched to each Rab tested (Fig. 3; Tables S1 and S2). TBC1D5 (a Rab7 GAP; Müller and Goody, 2018), was identified as a high-confidence Rab4 proximal protein (Fig. 3) and TBC1D8 (specificity unknown) was a lower-confidence association (longlist only, Table S2). AKAP10, the *Drosophila* homologue of which has been proposed to act as a Rab4 GAP and Rab11 GEF (Eggers et al., 2009), was significantly enriched to all Rabs, but was the most abundant with Rab4 (Fig. 3). TBC1D15 (a Rab11a/b GAP; Müller and Goody, 2018) was identified as proximal to only Rab25, whereas TBC1D2B (a Rab22 GAP; Kanno et al., 2010), RabGAP1L (TBC1D18; a Rab22a, Rab34 and Rab39b GAP; longlist only), TBC1D5 (longlist only), TBC1D22B (specificity unknown, longlist only) were Rab11 and Rab25 proximal (Fig. 3; Tables S1 and S2). These data highlight the overlapping regulation of Rab GTPases by specific GAPs, but also point to a more complex and interconnected regulation between Rab-specific GEFs and GAPs.

Protein complex formation between Rab GTPases and GEFs/GAPs is not necessarily indicative of a substrate: enzyme association and, in fact, Rab and Arf cascades have been reported to operate via GTPase–GEF/GAP associations (D'Souza et al., 2014; Knödler et al., 2010; Ortiz et al., 2002). Exemplifying this, in addition to Rab regulators, GEFs and GAPs for other Ras superfamily members were also identified (Fig. 3; Tables S1 and S2). These were predominantly regulators of the Arf and Rho families, perhaps pointing towards the influence of vesicle trafficking on cytoskeletal signalling (Jacquemet et al., 2013; Wu et al., 2011) and Rab–Arf interconnected cascades (D'Souza et al., 2014). Rho GTPase regulators included ARHGAP1 [p50RhoGAP, previously identified as a vesicular protein (Sirokmány et al., 2006), enriched to all Rabs but most abundant with Rab11 and Rab25], ARHGAP12 (Rab25 only), ARHGAP18 (Rab11 longlist only) and ARHGEF1 (p115RhoGEF, Rab11 longlist only). Arf regulators included ARFGEF1 (BIG1, Rab11 only), ARFGEF2 (BIG2, Rab4 longlist only), ARFGAP1 (Rab11 and Rab25 longlist only) and ARFGAP3 (all Rab longlists only).

#### Multiprotein trafficking complexes

Rab11FIP5 of the FERARI complex was highly enriched to Rab11 and Rab25 (but not Rab4) (Fig. 3), and the FERARI complex components EHD1 (Rab11 and Rab25 longlists only) and VPS45 (Rab11 longlist only) were also identified, confirming the ability of BioID to identify trafficking complexes. Interestingly, VPS33B (CHEVI complex subunit) was recruited by Rab11 (longlist only) and VPS8 was identified with all Rabs (Fig. 3; Rab25 longlist only Table S2). VPS51 (Ang2), a component of the EARP tether complex, previously seen to localise to Rab4 and Rab11 endosomes (Schindler et al., 2015), was enriched to all Rabs (longlists only) together with its interaction partner tSNARE syntaxin 6 (STX6; longlists only). Interestingly, retriever and COMMD complex components were not identified, but SNX1, SNX2 and SNX6 were robustly identified with all Rabs (Fig. 3; Tables S1 and S2). SNX3 (longlists only) also associated with all Rabs, whereas SNX4 (Rab11 longlist), SNX29 (Rab4 longlist), FAM21A (or WASHC2A, WASH complex component, Rab11 and Rab25 longlist) were more selectively recruited. These data point towards a previously unknown association between the Rab4, Rab11 and Rab25 recycling machinery and sorting nexins associated with the ESCPE-1 (SNX1/SNX2/SNX6) and retromer (SNX3) recycling complexes.

### Detection of directly biotinylated peptides reveals proximal domains between interactors

**Fig. 4.**
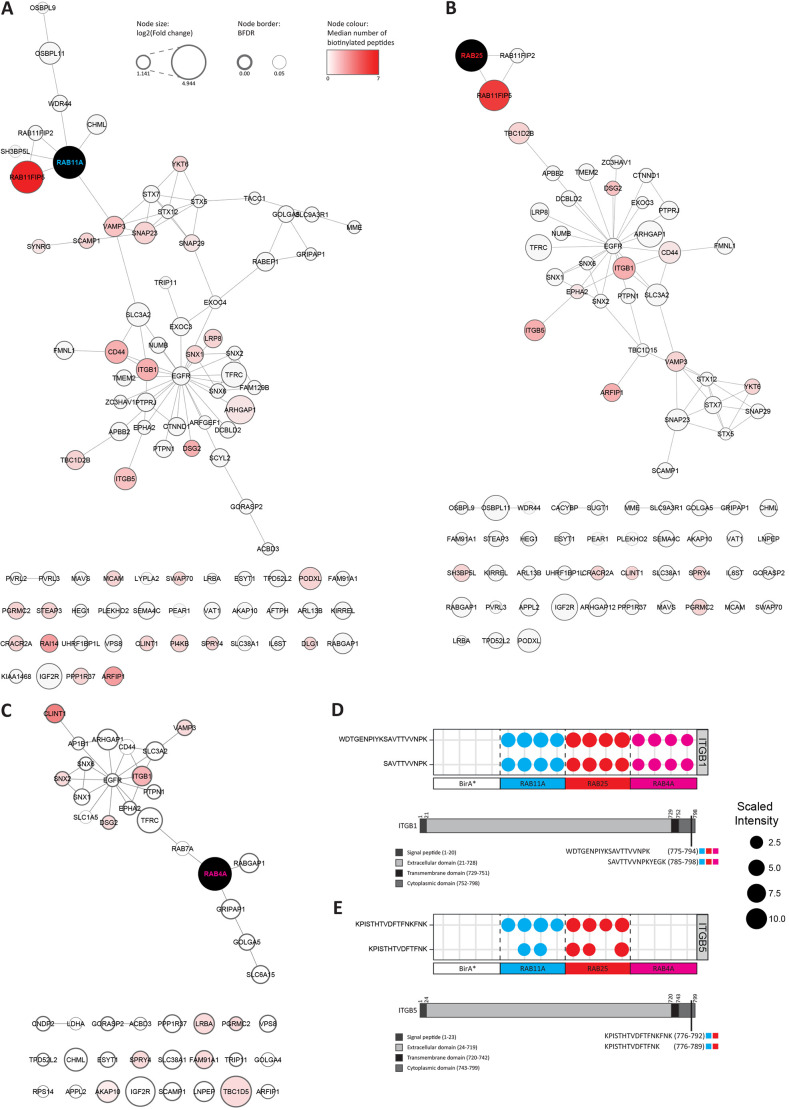
**Biotin-modified peptides reveal proximal interactors within PPI networks.** (A–C) High-confidence Rab4a (A), Rab11a (B) and Rab25 (C) proximal proteins (Fig. 1B) were mapped onto a PPI database. Rab GTPase nodes are coloured black, other nodes are the high-confidence proximal proteins identified for each Rab GTPase. Edges represent interactions present within the PPI database. Node sizes represent log_2_(fold change) values, node borders represent BFDR values from SAINTexpress analysis, and node colours represent the median numbers of biotinylated peptides identified. (D,E) Biotinylated integrin β1 (D) or β5 (E) peptides were mapped to protein domains. Protein domains are shown by grey colourings; domains are not to scale. The positions of the biotinylated peptides in the protein are indicated, and coloured boxes show the samples in which they were identified; Rab11a in blue, Rab25 in red, and Rab4a in pink.

BioID involves direct biotinylation of lysine residues in close proximity to the bait protein, which can be clearly detected as a modification by mass spectrometry. For the majority of proteins, the biotin modification was not specifically identified on a peptide; however, for the peptides it was identified on, this would indicate a more significant higher affinity and/or a longer-term or repeated interaction (Fig. S3A). This analysis not only revealed the overlapping interaction domains involved between associated trafficking machinery and/or cargoes and Rab4, Rab11 and Rab 25, but also highlighted key differences in proximal proteins (Fig. 4A–C; Fig. S3B–H; Table S3).

Rab11FIP5 is a key effector of Rab11 family GTPases and was heavily biotinylated by both Rab11a and Rab25 but not by Rab4a (Fig. 4A–C; Fig. S3C) in intrinsically disordered regions (predicted by AlphaFold 2) within 200 residues of the C-terminal Rab-binding domain, but away from the N-terminal C2 domain, consistent with the known site of interaction (Prekeris et al., 2001). Cargoes such as ITGB1 (β1-integrin) were detected in similar abundance between Rabs and showed a remarkably consistent level of biotinylation in two peptides that overlap (Fig. 4D) within the cytoplasmic domain of the integrin (Fig. S3B) that was previously found to interact with Rab25 (Caswell et al., 2007). We mapped the Rab25 interaction site within β1-integrin to a 17-amino-acid region encompassing the membrane-proximal NPIY motif (Fig. S4A) and, interestingly, this showed good overlap with proposed biotinylated lysines, suggesting that BioID does indeed provide insight into the interaction sites of cargoes and machinery. ITGB5 was detected as a biotinylated cargo for only Rab11 and Rab25 (Fig. 4E; Fig. S3B), indicating that this integrin might be more selectively recycled via Rab11 family members.

Rab4 showed selective biotinylation of FAM91A1, previously identified on Golgi recruited to endosomal vesicles (Shin et al., 2017) (Fig. 4C). Furthermore, although clathrin interactor 1 (CLINT1 or epsinR) was identified as a Rab4-, Rab11- and Rab25-proximal protein (Fig. 4), it was more heavily biotinylated close to the cargo-selective N-terminal ENTH domain by Rab4 (Fig. 4C; Fig. S3D). CLINT1 interacts with the clathrin adaptor AP1 to mediate trans Golgi network–lysosome trafficking of cathepsin D (CTSD) and localises to EEs to control retrograde trafficking (Mills et al., 2003), but its function with regard to Rab4 is not clear.

PI4KB, a phosphoinositide-4 kinase, was selectively biotinylated by Rab11a (Fig. 4A; Fig. S3E), suggesting a specific involvement in Rab11a-mediated processes. Interestingly, PI4KB plays a critical role in recruiting Rab11 and its effectors to Golgi membranes (Graaf et al., 2004), suggesting that the interaction of Rab11 with and function on this organelle is not shared with the closely related Rab25. Similarly, VPS51 was found to be selectively biotinylated by Rab25 (Fig. S3F), which indicates a closer or persistent interaction of Rab25 with the EARP complex. Both Rab11a and Rab25 (but not Rab4) identified CRACR2A as a potential interactor (Fig. S3G). Interestingly, of the potential GEFs, only SH3BP5L was biotinylated, and only by Rab25. This biotinylation occurred within the GEF domain and suggests that SH3BP5L is a more dominant Rab25 GEF and/or that the interaction is repeated or more stable (Fig. 4B; Fig. S3H). The Rab11a–SH3BP5 structure identified contact residues in SH3BP5 that form the coiled coil required for recognition, and these are conserved in SH3BP5L (Jenkins et al., 2018; Fig. S4B). Interestingly, multiple sequence alignment using Clustal indicates that the biotinylated residues identified here have sequence similarity with the SH3BP5 C-terminus but are situated on the opposite side of the fold, suggesting that Rab25 binding to SH3BP5L occurs at a different site (Fig. S4B). Of the putative GAPs, only TBC1D5 was biotinylated by Rab4, and only TBC1D2B by Rab11 and Rab25 (Fig. 4A–C; Table S3). SNARE proteins showed a varying degree of selective proximity: YKT6 and VAMP3 were biotinylated by all Rabs, VAMP2 appeared more proximal to Rab11a and Rab25, and SNAP23 and SNAP29 were most heavily biotinylated by Rab11a (Fig. 4A–C; Table S3). Taken together, these data indicate that although the abundance of prey proteins in BioID experiments can be extremely informative, close analysis of biotinylated peptides and residues provides an additional level of granularity that could be important in deducing functional interactions.

### Knock-sideways relocalisation of bait can validate high-affinity prey interactions

**Fig. 5.**
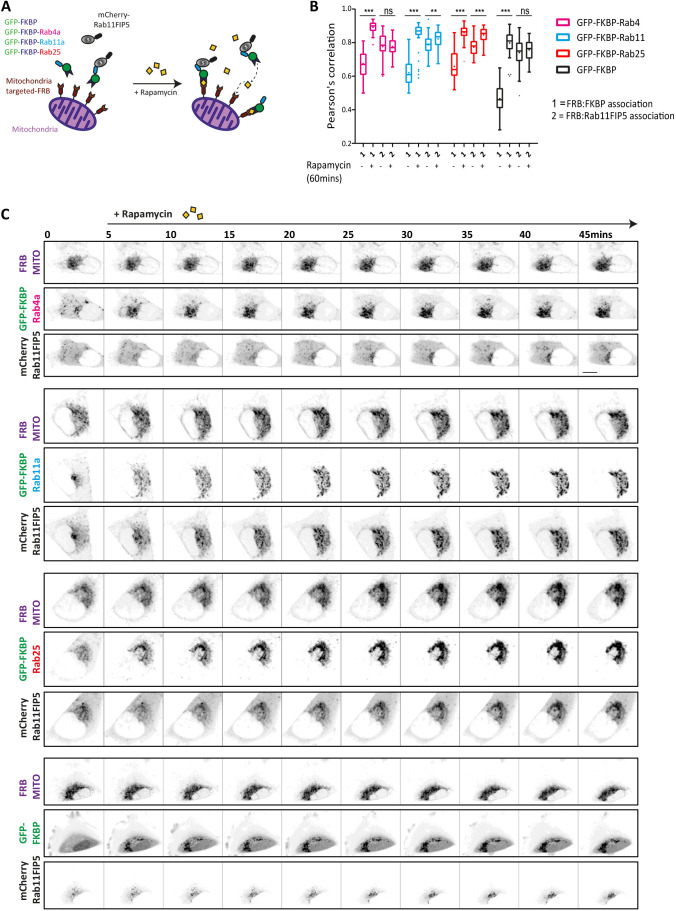
**Knock-sideways validation of proximity labelling-identified preys.** (A) Schematic illustration of knock-sideways, where mitochondria-targeted FRB/rapamycin is used to induce the re-localisation of GFP–FKBP-tagged Rabs and associated protein complexes. (B,C) A2780 cells expressing iRFP670–FRB and GFP–FKBP or GFP–FKBP-tagged Rab4a, Rab11a or Rab25 and mCherry–Rab11FIP5 were imaged by spinning-disk confocal microscopy, before and after the addition of rapamycin (200 nM) to visualise the redistribution of GFP-tagged proteins and the corresponding localisation of mCherry–Rab11FIP5. Re-distribution of GFP–FKBP fusion (1) and mCherry–Rab11FIP5 (2) were analysed by Pearson's correlation (at least 30 cells/condition). Box plots are presented as described as in Fig. 2. Statistical analysis was performed with one-way ANOVA with Holm–Sidak post hoc test. ns, not significant; **P<0.01; ***P<0.001. (C) Representative images from at least three independent timelapse experiments are shown. Scale bar: 10 µm.

Conventional fluorescence microscopy provides good insight into the co-distribution of proteins within cells and is valuable as a tool to validate the types of interactions detected by BioID, which are often missed by conventional pull-down strategies. However, colocalisation analysis is insufficient to provide information on the relative strengths of interactions within cells. We therefore adapted the knock-sideways approach (Robinson et al., 2010), wherein a mitochondrially targeted FRB domain is co-expressed with an FKBP domain fused to a ‘bait’ protein of interest, and rapamycin treatment triggers binding of FKBP to FRB and mitochondrial targeting of the bait, reasoning that strong and/or repeated prey interactors would be more likely to follow their ‘bait’ upon relocalisation to mitochondria (Fig. 5A). We first focused on Rab11FIP5 as this is a known high-affinity interactor of (active) Rab11a and Rab25 but not Rab4. Co-expression of FRB–mito with GFP–FKBP–Rab proteins and mCherry–Rab11FIP5 revealed that Rab proteins quickly redistributed to mitochondria upon addition of rapamycin [Rab4, T_1/2_ (time necessary to relocalise 50% of total GFP signal to mitochondria)=2.3±0.4 min (mean±s.e.m.); Rab11, T_1/2_=4.3±0.5 min; Rab25, T_1/2_=5.4±0.1 min], whereas mCherry–FIP5 redistributed to mitochondria over a slower timescale (T_1/2_=13–26 min), but only when co-expressed with GFP–FKBP–Rab11a (T_1/2_=13.88±1 min) or GFP–FKBP–Rab25 (T_1/2_=24.3±2.3 min) (Fig. 5B,C; Fig. S5A). When relocalisation of endogenous Rab11FIP5 was assessed in knock-sideways experiments, relocalisation was again only observed in the presence of rapamycin when GFP–FKBP–Rab11a and GFP–FKBP–Rab25 were expressed (Fig. S5B–D). We next tested a second known interactor, PI4KB, which our data indicated might specifically associate with Rab11a but not Rab4a or Rab25. Indeed, whereas GFP–FKBP–Rab11a redistributed endogenous PI4KB to mitochondria in the presence of rapamycin to a significant extent, Rab4a and Rab25 were inefficient in this regard (Fig. S5E). These data indicate that knock-sideways relocalisation of bait proteins can act as a tool to validate potential interactions identified by BioID and strengthen the notion that PI4KB is a more robust interactor of Rab11a than of Rab25.

**Fig. 6.**
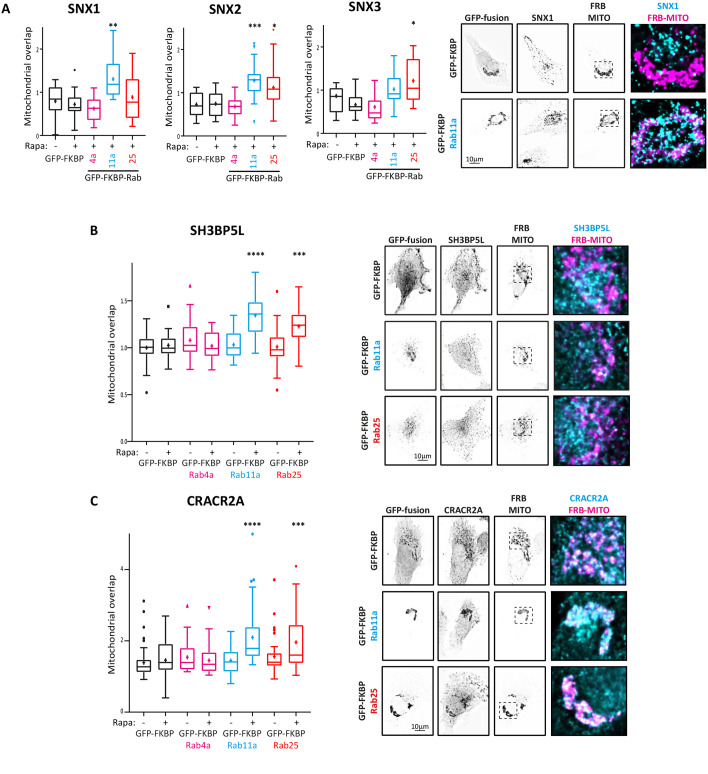
**Trafficking machineries are selectively recruited to recycling Rabs.** (A–C) A2780 cells expressing iRFP670–FRB and GFP–FKBP–Rab proteins treated with rapamycin (200 nM, 4 h) were fixed and stained for endogenous SNX1, SNX2 or SNX3 (A), SH3BP5L (B) or CRACR2A (C). Redistribution of candidate trafficking machinery was analysed by quantifying mitochondrial overlap (see Materials and Methods; 9–47 cells/condition). Box plots are presented as described as in Fig. 2. Statistical analysis is indicated compared to GFP–FKBP +rapamycin control using Kruskal–Wallis test with Dunn's multiple comparisons. *P<0.05; **P<0.01; ***P<0.01; ****P<0.0001. Representative images from at least three independent experiments are shown. Scale bars: 10 µm.

We next tested knock-sideways efficiency in the relocalisation of sorting nexins that were strong hits in BioID abundance, but more variably biotinylated. SNX1 was identified by Rab4a, Rab11a and Rab25 (Fig. 3) but only showed relatively consistent biotinylation with Rab11a (Fig. 4A–C). Consistent with this, GFP–FKBP–Rab11a significantly redistributed endogenous SNX1 to mitochondria in the presence of rapamycin, but Rab4a and Rab25 knock sideways did not induce mitochondrial localisation (Fig. 6A). SNX2 showed similar abundance across Rabs, but SNX2 was only rerouted to mitochondria in cells expressing GFP–FKBP–Rab11a and GFP–FKBP–Rab25 (Fig. 6A; Fig. S6A). SNX3 was identified by all Rabs (longlist only), but only significantly redistributed in GFP–FKBP–Rab25-expressing cells (Fig. 6A; Fig. S6B). Endogenous SH3BP5L (Fig. 6B) and CRACR2A (Fig. 6C) were significantly redistributed to mitochondria by GFP–FKBP–Rab11a and GFP–FKBP–Rab25, but not GFP–FKBP–Rab4a. This is consistent with BioID data (Fig. 3); however, biotinylated SH3BP5L peptides were only identified for Rab25, whereas biotinylated CRACR2A peptides were observed for both Rab11a and Rab25 (Fig. S3G,H). Our Rab4 BioID network showed enrichment of CLINT1 and its interactor AP1B1 (Figs 1B and 3), and CLINT1 was itself directly biotinylated in Rab4 samples (Fig. 4C; Fig. S3D); however, knock-sideways experiments showed that Rab4a was unable to induce significant re-localisation of CLINT1 (Fig. S6C), despite good colocalisation between Rab4 and CLINT1 (Fig. S6D). Taken together, these data confirm key findings of proximity labelling but also point towards a more complex relationship among BioID abundance, direct detection of biotinylation, the affinity of bait for prey and the ability to redistribute potential prey proteins.

### Rab4-, Rab11- and Rab25-associated machinery is required for cell migration within the 3D CDM

Endocytic recycling pathways regulated by Rab4, Rab11 and Rab25 have each been implicated in cell migration and invasion in the 3D matrix. Rab4 controls Rac-driven lamellipodial migration of cells in 2D and invasion in low FN through the recycling of αvβ3, whereas Rab11 and Rab25 control α5β1 trafficking to influence filopodia formation and migration in a high-FN 3D matrix (Fig. 2, Caswell et al., 2007, 2008; Christoforides et al., 2012; Dozynkiewicz et al., 2012). We therefore selected Rab4-, Rab11- and Rab25-associated proteins from our dataset that had not previously been implicated in cell migration and/or invasion for further analysis in cells that lack Rab25 (A2780-DNA3 cells, in which migration is supported by Rab4; Christoforides et al., 2012) and Rab11 (Gemperle et al., 2022), or express Rab25 at levels similar to those found in ovarian cancer (Cheng et al., 2004) (A2780-Rab25) and migrate in a Rab25/α5β1-dependent manner (Caswell et al., 2007; Dozynkiewicz et al., 2012).

**Fig. 7.**
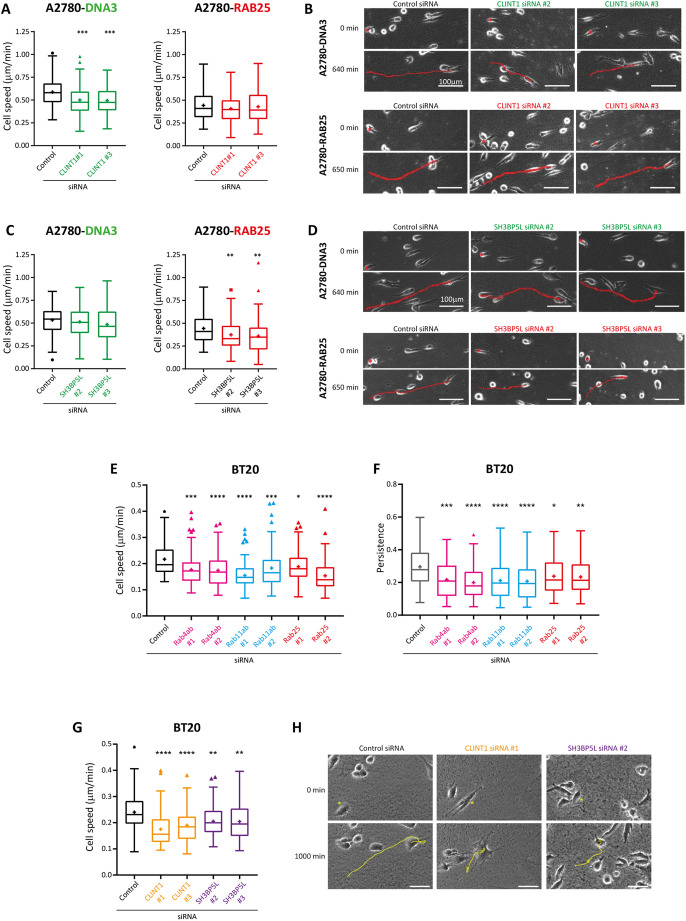
**Rab4- and Rab25-specific machinery is required for cell migration in the 3D matrix.** (A–D) A2780-DNA3 and A2780-Rab25 cells were depleted of CLINT1 (A,B) or SH3BP5L (C,D) by siRNA and seeded into CDM for 4 h, before migration was analysed by brightfield time-lapse imaging for >16 h. Cell speed was analysed by manual tracking (A,C). Representative images from at least three independent experiments are shown (B,D). Red lines indicate the migration of cells marked with asterisks. Scale bars: 100 µm. (E,F) BT20 cells were depleted of Rab4a and Rab4b (indicated as Rab4ab), Rab11a and Rab11b (indicated as Rab11ab), or Rab25 by siRNA and migration analysis performed as above to determine cell speed (E) and cell persistence (F). (G) BT20 cells were depleted of CLINT1 or SH3BP5L by siRNA and migration analysis performed as above. (H) Representative images of BT20 cells analysed in G. Yellow lines indicate the migration of cells marked with asterisks. Scale bars: 50 µm. For A–H, *n*≥90 cells from three independent experiments. Box plots are presented as described as in Fig. 2. Statistical analysis was performed with Kruskal–Wallis and Dunn's multiple comparisons test. *P<0.05; **P<0.01; ***P<0.001; ****P<0.0001.

Because CLINT1 was enriched to Rab4a, was directly and extensively biotinylated in Rab4 samples, and colocalised with Rab4 (Figs 3 and 4C; Figs S3D and S6D), we focused on the role that CLINT1 plays in cell migration in the 3D CDM. Knockdown of CLINT1 with either of two siRNA oligonucleotides significantly reduced cell migration in the 3D CDM (Fig. 7A,B; Fig. S7B), but only in cells that lacked Rab25. This is consistent with the notion the CLINT1 plays a role in Rab4 (but not Rab25)-dependent migration in the 3D CDM. We next investigated a Rab11a-specific candidate, PI4KB, which was enriched to and directly biotinylated in Rab11 samples (Fig. 3; Figs S3E and S4B), and could be significantly re-localised by knock-sideways of Rab11a alone (Fig. S5E). Knockdown of PI4KB, however, did not impact migration (Fig. S7A).

SH3BP5L is an interesting candidate shown to behave as a Rab11a- or Rab25-specific GEF *in vitro* (Jenkins et al., 2018). Our data indicate that SH3BP5L is significantly enriched to Rab11a but to a greater extent with Rab25 (Fig. 3), and that although both Rab11a and Rab25 knock-sideways-relocalised SH3BP5L (Fig. 6B), biotinylated SH3BP5L peptides only appeared in Rab25 samples and hinted at a different mode of interaction to that of Rab11 and SH3BP5L (Fig. 4B; Figs S3H and S4A). SH3BP5L knockdown had no effect on 3D migration in cells lacking Rab25 but significantly decreased migration of cells migrating in a Rab25-dependent manner (Fig. 7C,D; Fig. S7B). These data suggest that SH3BP5L could be specifically required to maintain Rab25 activity in cells migrating in the 3D matrix.

We next sought to confirm our findings using BT20 triple-negative breast cancer cells, which express Rab4a, Rab4b, Rab11a, Rab11b and Rab25 (Fig. S7D). BT20 cells maintain directional persistence as they migrate in the 3D CDM, but depletion of Rab4a, Rab4b, Rab11a, Rab 11b or Rab25 decreased both speed and persistence of motility (Fig. 7E,F; Fig. S7C,D), indicating that migration of this cell line requires each of the Rab GTPase subfamilies. Depletion of the Rab4-recruited trafficking machinery CLINT1 or the Rab25 GEF SH3BP5L reduced the speed of migration of BT20 cells in the 3D CDM (Fig. 7G,H; Fig. S7E), suggesting that their function in motility is conserved.

**Fig. 8.**
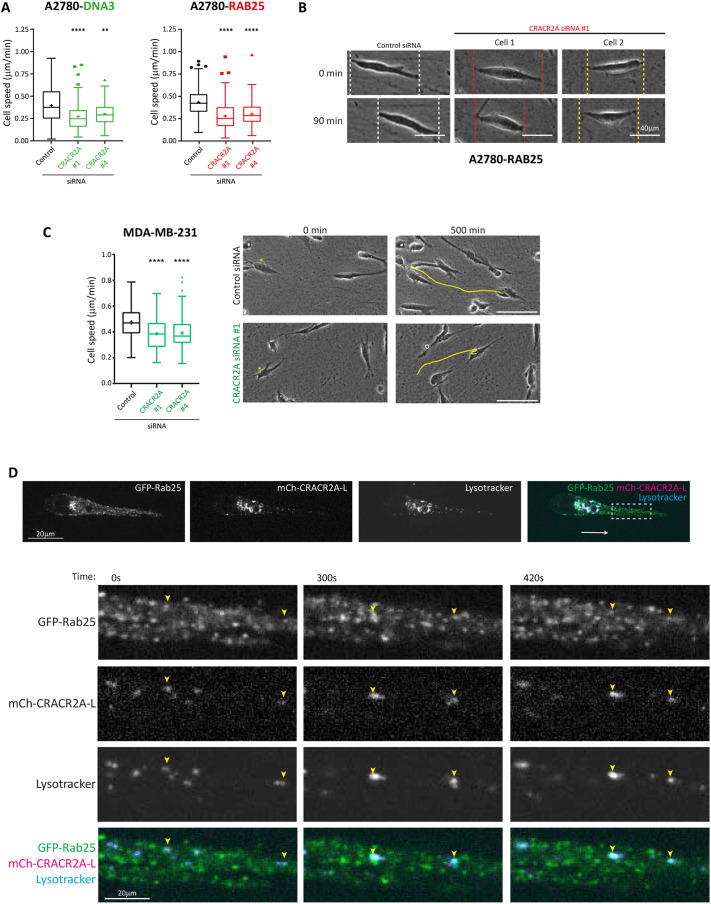
**CRACR2A is required for cell migration in the 3D matrix.** (A,B) A2780-DNA3, A2780-Rab25 (A,B) and MDA-MB-231 (C) cells were depleted of CRACR2A by siRNA and seeded into the CDM for 4 h, before migration was analysed by brightfield time-lapse imaging for >16 h. Cell speed was analysed by manual tracking. *n*≥90 cells from three independent experiments. Box plots are presented as described as in Fig. 2. Statistical analysis was performed with Kruskal–Wallis and Dunn's multiple comparisons test. **P<0.01; ****P<0.0001. Representative images from at least three independent experiments are shown. Dotted lines (B) indicate the extremities of each cell at that timepoint. Yellow lines (C) indicate the migration of cells marked with asterisks. Scale bars: 40 µm (A,B); 100 µm (C). (D) A2780 cells expressing GFP–Rab25 and mCherry–CRACR2A were seeded into the CDM for 4 h before time-lapse imaging by spinning-disk confocal microscopy. The dashed white box indicates the area enlarged in lower panels. The white arrow indicates the direction of migration; yellow arrowheads indicate the positions of CRACR2A-positive late endosomes and/or lysosomes that show overlap with Rab25. Images are representative of three independent experiments. Scale bars: 20 µm.

Our data suggest that CRACR2A association is highly significant to both Rab11a and Rab25 to almost identical extents (Figs 1B, 3 and 6C; Fig. S3G). Interestingly, CRACR2A knockdown significantly suppressed migration in the 3D CDM in both Rab25-expressing and non-expressing A2780 cells (Fig. 8A,B; Fig. S8A,B). CRACR2A was also required to support migration of MDA-MB-231 breast cancer cells (which lack Rab25 expression, but express high levels of CRACR2A) in the 3D CDM (Fig. 8C; Fig. S8C). Interestingly CRACR2A depleted cells showed a ‘stalling’ phenotype (Fig. 8B; Fig. S8A), reminiscent of Rab25-expressing cells that lack the lysosomal protein CLIC3 (Dozynkiewicz et al., 2012). mCherry-tagged CRACR2A localised to lysosomes within the cell body (similar to CLIC3 distribution; Dozynkiewicz et al., 2012), and to LysoTracker-positive vesicles that also contained Rab25 towards the leading edge of pseudopodial protrusions generated in the CDM (Fig. 8D). These data suggest that the dynein adaptor CRACR2A is required for efficient migration and might associate with Rab25 to deliver cargoes for trafficking to lysosomes.

## DISCUSSION

Here, we show that BioID can be deployed to comparatively analyse the interactomes of Rab family GTPases and reveal new trafficking machinery that controls cell motility in the 3D matrix. This generated a robust catalogue of Rab4a-, Rab11a- and Rab25-associated proteins within migratory A2780 ovarian cancer cells, providing an important resource for further understanding of how trafficking networks perform their myriad functions, including in cell migration.

GO analysis of the cohorts of Rab4a-, Rab11a- and Rab25-associated proteins were unsurprisingly dominated by vesicle trafficking-related terms, but terms associated with the cytoskeleton (e.g. ‘leading edge’, ‘lamellipodium’) and focal adhesions were also identified. This prompted us to investigate focal adhesion formation and F-actin organisation within protrusions. Owing to technical limitations related to imaging focal adhesions in the 3D CDM, we analysed the focal adhesion marker paxillin in cells on 2D substrates. Whereas Rab11 and Rab25 expression levels did not impact focal adhesions, knockdown of Rab4a and Rab4b reduced the number of paxillin-positive cell–matrix adhesions (Fig. S2D). This agrees with previous work demonstrating that Rab4 recycles αvβ3 integrins and that αvβ3 trafficking is linked to adhesion formation (Gu et al., 2011; Woods et al., 2004). Rab11 and Rab25 have been implicated in the recycling of α5β1 integrin, and future studies are required to resolve whether this integrin is more prominent in cell–matrix adhesion complexes in 3D. Depletion of Rab4 and Rab11, or expression of Rab25 at levels similar to those seen in aggressive ovarian cancer (Cheng et al., 2004), had significant effects on the architecture of F-actin within protrusions of cells moving in the 3D CDM. Rab4a and Rab4b knockdown reduced the width of the protrusions and reduced the frequency with which protrusions were tipped by lamellipodia, increasing filopodial protrusions. Rab11a and Rab11b knockdown, however, increased the width of protrusions and increased the occurrence of lamellipodial protrusions, with fewer cells extending protrusions tipped by filopodia alone. In line with this, Rab25 overexpression increased filopodia-tipped protrusions at the expense of lamellipodia. These data concur with previous observations that Rab4 and Rab11 recycling pathways are antagonistic (Caswell et al., 2008; Christoforides et al., 2012; White et al., 2007) and that Rab11 (and RCP)-dependent trafficking of α5β1 favours the formation of filopodial protrusions to promote invasive migration (Jacquemet et al., 2013; Paul et al., 2015b).

Proximity labelling methods have proven to be an excellent tool for the identification of protein complexes, including for Rab4 and Rab11 GTPases (Gillingham et al., 2019; Go et al., 2021; Liu et al., 2018; Del Olmo et al., 2019). Two studies include both Rab4a and Rab11a in their analyses: Go et al. (2021) use BioID to map the proteome in HEK-293 cells (spectral counting-based quantification, two replicates), whereas Liu et al. (2018) combine affinity purification and BioID in a ‘multiple approaches combined’-tagging strategy (HEK-293 and cancer cell lines, spectral counting-based quantification, two replicates), but neither allow for direct quantitative comparison between GTPases. MitoID combines BioID with mitochondrial tagging to provide a standardised subcellular localisation, and was used to identify potential interaction partners of active and inactive Rab11 mutants in HeLa cells (spectral counting-based quantification, three replicates) (Gillingham et al., 2019), and an APEX2-Rab4a dataset was also obtained in HeLa cells (spectral counting-based quantification, three replicates) (Del Olmo et al., 2019). Our data show good overlap with these studies but differ primarily in the direct comparison between Rab4a, Rab11a and Rab25 analysed using quantitative label-free proteomics to generate a statistically robust interaction network linking these three recycling regulators. Interestingly, CLINT1 was identified as a Rab4-proximal protein in other datasets (Go et al., 2021; Del Olmo et al., 2019), ESCPE-1 and retromer complex members have been identified with Rab11 and Rab4 baits (Gillingham et al., 2019; Go et al., 2021; Del Olmo et al., 2019), and the CRACR2A:Rab11a proximity was also found by Go et al. (2021). Our study furthers this complementarity by using directly biotinylated peptides to provide another level of confidence, for example, the association between Rab4a and Rab11a/Rab25 with the GAPs TBC1D5 and TBC1D2B, respectively. Furthermore, we report the first description of protein interaction networks formed by the cancer-relevant protein Rab25.

Rab11a and Rab25 share 66% sequence identity at the protein level, differing primarily in their membrane-targeting C-termini. It is therefore unsurprising that significant overlap is seen within the cohorts of associated proteins, with particularly high correlation between the level of enrichment to Rab11a and Rab25 for the 31 proteins recruited to Rab4a, Rab11a and Rab25 (whereas recruitment to Rab4a was less well correlated; Fig. S1I). In light of this, it is therefore surprising that Rab11a was not found to associate with Rab25, even though there is an indication of Rab4 association with both Rab11a and Rab25. Dimeric Rab11FIP structures have been described to recruit two copies of Rab11 to form a tetramer, but our data hint that these might be homotypic in nature, such that Rab25 and Rab11a might function separately. GO analysis indicates that, at least in the migratory cancer cells used in these experiments, the primary functions of these recycling Rabs relate not only to vesicle and endosome function, but also to focal adhesion and leading edge-related processes, suggesting broad links to adhesion and migration (Fig. S2). In addition, ‘amino acid transport’ is a significantly enriched GO term for Rab4a-associated proteins (Fig. S2), indicating that transporter trafficking might constitute an important role of Rab4a that is relatively understudied.

Our dataset includes biotin-modified lysine residues detected, which can be used to infer proximity between proteins. Although this does not necessarily map to sites of direct interaction, we noticed that Rab11FIP5 was extensively biotinylated by its interaction partners Rab11a and Rab25 but not Rab4a, and this occurred primarily in an unstructured region close to the known Rab11/Rab25 interaction motif (Fig. S3C). Because we had previously shown that Rab25 and β1 integrin interact directly, we further mapped the Rab25-binding region in the β1 cytoplasmic tail to a 17-amino-acid stretch covering the membrane-proximal NPXY motif and one of the potential lysine biotinylation sites identified by BioID (with other potential sites of biotinylation within 14 residues; Fig. S4A). This suggests that BioID can provide a good indication of specific binding interfaces. The structure of Rab11a in complex with SH3BP5 (closely related to SH3BP5L) has been solved (Jenkins et al., 2018). We therefore analysed this interaction site and the biotinylation pattern found in SH3BP5L for Rab25 (Fig. S4B). Interestingly, this analysis suggests that the Rab25:SH3BP5L interaction motifs share homology but are distinct from the Rab11a:SH3BP5 binding site. This could explain why SH3BP5L is only biotinylated by mycBirA*–Rab25 and why SH3BP5L appears to be specifically required for Rab25 (but not Rab11)-mediated migration (Fig. 7C,G,H).

### New trafficking machinery linked to Rab4a, Rab11a and Rab25

In addition to well-characterised trafficking machinery previously implicated in Rab4/Rab11/Rab25 recycling, our datasets also provide novel links between the recycling Rabs and other proteins or complexes implicated in different aspects of vesicle trafficking, and new twists on known interactions. For example, the EARP complex component VPS51 is enriched to all Rabs tested (Table S2), but only biotinylated directly by Rab25 (Fig. S3F), which suggests a more direct role for EARP in Rab25-mediated trafficking events. Sorting nexins such as SNX4 (identified here in the Rab11 longlist) have previously been associated with Rab11-dependent recycling (van Weering et al., 2012; Solis et al., 2013), but our experiments also identified ESCPE-1 components (specifically SNX1/SNX2/ SNX 6) and the retromer component SNX3 (Figs 1B, 3, 4 and 6A; Fig. S6A,B). Both ESCPE-1 and retromer function in tandem with the WASH complex, of which FAM21A was detected for Rab11a and Rab25. In fact, SNX1 was biotinylated in three of four Rab11 samples and selectively re-localised by Rab11a knock sideways. Both Rab11a and Rab25 were able to re-localise SNX2, whereas Rab4 was not able to do so. This points towards a closer association between ESCPE-1 and Rab11a/Rab25 than previously anticipated. SNX3, however, was re-localised by Rab25. The retromer complex can mediate delivery of endocytic cargoes to late endosomes and, interestingly, Rab25 is involved in the sorting of activated α5β1 integrin to late endosomes (Dozynkiewicz et al., 2012), suggesting that Rab25 and retromer act in concert. Further investigation of these new links between Rab recycling and ESCPE-1/retromer complexes might help to build a more integrated view of how cargoes can be routed along the endosomal pathway.

### Proximity labelling identifies functionally relevant associations in migrating cells

Rab4, Rab11 and Rab25 have each been implicated in cell migration, in particular in 3D matrices, by controlling the trafficking of cargoes such as integrins. Because our datasets included integrins, and the cohort of associated proteins was enriched for GO terms such as ‘cell leading edge’ and ‘focal adhesion’, we reasoned that many of the associated proteins could be involved in motility. Rab4 is required for directional lamellipodial migration by trafficking αvβ3 integrin (Christoforides et al., 2012; White et al., 2007), whereas Rab25 expression promotes a different mode of migration characterised by pseudopod extension in the 3D matrix (Caswell et al., 2007; Dozynkiewicz et al., 2012). Rab11, through its effector Rab coupling protein, controls recycling of α5β1 integrin and cell migration or invasion in cells that express mutant p53, or when Rab4 or αvβ3 are inhibited (Caswell et al., 2008; Muller et al., 2009), but we have also found that Rab11 is required for efficient migration in the absence of these factors (Gemperle et al., 2022). We therefore investigated the ability of cells to migrate in a 3D CDM in the presence or absence of Rab25 expression after knockdown of candidate machinery closely linked to Rab4a/Rab11a/Rab25 through our experiments, reasoning that Rab25-interacting proteins could be required for migration in the context of Rab25 expression alone and that the Rab4-related machinery might become redundant when Rab25 is expressed. Indeed, SH3BP5L, a reported Rab25 GEF *in vitro* that is selectively biotinylated and re-localised by Rab25, had no effect on basal migration of A2780-DNA3 cells but suppressed migration of A2780-RAB25 cells (Fig. 7C,D). Similarly, CLINT1, enriched and more highly biotinylated by Rab4a, was required for migration in the 3D CDM, but only in the absence of Rab25 expression (Fig. 7A,B). In a different cell type, BT20 breast cancer cells, knockdown of Rab4a, Rab4b, Rab11a, Rab11b or Rab25 significantly decreased motility, as did knockdown of CLINT1 or SH3BP5L. This confirms that the importance of each is generalisable to motility of other cell types, but the reliance of BT20 cells on CLINT1 suggests that there could be differences in the way that the two cell types tested here rely on trafficking pathways to migrate. Endogenous Rab25 is expressed in BT20 cells, albeit at a lower level than in the A2780-Rab25 line, in which overexpression levels reflect those found in aggressive ovarian cancers. The difference in migration could therefore reflect expression level, and it is interesting to speculate that high expression of Rab25 could overcome the requirement for the Rab4 pathway in motility (as is the case when Rab11-RCP trafficking is activated; Caswell et al., 2008; Christoforides et al., 2012). Interestingly, the selective Rab11-associated protein PI4KB was not required for migration (Fig. S7A), which could reflect its role in Golgi-mediated processes rather than recycling.

The dynein adaptor CRACR2A is linked similarly to Rab11 and Rab25 (but not Rab4a) through our proteomic, biotinylation and re-localisation data (Fig. 3). Interestingly, knockdown of CRACR2A reduced migration in the 3D CDM in both the presence and absence of Rab25. Our findings further indicate that CRACR2A resides predominantly on a late endosomal or lysosomal compartment. Rab25 endosomes periodically associate with CRACR2A late endosomes, suggesting that cargo exchange occurs in an analogous manner to that seen for Rab25 and CLIC3 in the trafficking of activated α5β1 integrin. How Rab11 might associate with such a pathway and which cargoes it might deliver is not known; however, given that the CLIC3-related protein CLIC4 is implicated in integrin trafficking and motility, it is possible that a parallel pathway handles alternative integrin cargoes in the absence of Rab25.

The findings presented here represent a robust resource cataloguing Rab4a-, Rab11a- and Rab25-associated proteins, which confirm previous findings but also create links to previously unrelated trafficking machineries. Our validation approach using knock-sideways allows for a functional appraisal of the relevance of candidates, which could be more broadly expanded and applied to confirm proximity labelling data, revealing dynamic interactors. Moreover, linking the robust identification of prey proteins with phenotypes associated with their specific baits has identified new players in the endocytic recycling pathways that regulate cell migration.

## MATERIALS AND METHODS

### Cell culture

A2780 (DNA3 and RAB25) ovarian cancer cell lines (Cheng et al., 2004) were cultured in Roswell Park Memorial Institute-1640 (RPMI-1640) medium (R0883, Sigma-Aldrich). A2780 cells transiently or stably expressing mycBirA* fusion proteins were cultured in RPMI-1640 medium without biotin (R9002-01, US Biological), made up according to the manufacturer's instructions. Telomerase-immortalised fibroblasts (TIFs; Caswell et al., 2007) were cultured in Dulbecco's modified Eagle eedium (DMEM; D5796, Sigma-Aldrich). The cell culture medium was supplemented with 10% (v/v) foetal bovine serum (FBS; Sigma-Aldrich), 1% antibiotic-antimycotic (Sigma-Aldrich), and 2 mM L-glutamine (Sigma-Aldrich). BT-20 [American Type Culture Collection (ATCC)] and MDA-MB-231 (ATCC) breast cancer cells were cultured in DMEM (D6429, Sigma-Aldrich) supplemented with 10% (v/v) FBS and ciprofloxacin (5 µg/ml, Sigma-Aldrich). Cells were maintained at 37°C in a humidified atmosphere with 5% (v/v) CO_2_. Biotin (Sigma-Aldrich) was added to the cell culture medium to give the final concentrations indicated for a total of 16 h.

### Antibodies and probes

A range of primary and secondary antibodies were used in this study for immunofluorescence (IF) and western blotting (WB): anti-c-Myc 9E10 (Sigma-Aldrich, M4439; WB, 1:1000; IF, 1:200) or 9B11 (Cell Signalling Technology, 2276S; WB, 1:1000; IF, 1:100); anti-Rab11 (Invitrogen, 715300; WB, 1:1000; IF, 1:200) anti-α-tubulin DM1A (Abcam, ab7291; WB, 1:5000) or YL1/2 (Abcam, ab6160; WB, 1:1000); anti-CLINT1 (Abcam, ab223088; WB, 1:500); anti-CLINT1 (Thermo Fisher Scientific, PA5-60308; IF, 1:200); anti-CRACR2A (Proteintech, 15206-1-AP; WB, 1:1000; IF, 1:200); anti-PI4KB (Proteintech, 13247-1-AP; WB, 1:500); anti-SH3BP5L (Atlas Antibodies, HPA038068; WB, 1:500; IF, 1:200); anti-RFP (5F8; Chromotek, 5f8-100; IF, 1:200); anti-Rab11FIP5 (Proteintech, 14594-1-AP; IF, 1:1000); anti-β-tubulin (Proteintech, 66240-1-19; WB, 1:10,000); anti-paxillin (BD Transduction Laboratories, 612405; IF, 1:200); and anti-EEA1 (Cell Signalling Technology, C45B10; IF, 1:200). Streptavidin DyLight-800 (1:5000, Thermo Fisher Scientific) was used for western blotting, and Streptavidin-Cy3 (1:200, Invitrogen) was used for immunofluorescence microscopy. Phalloidin Alexa Fluor 647 (Life Technologies, A22287; IF, 1:50), Hoechst 33342 (Invitrogen, H3570; IF, 1:1000), transferrin Alexa Fluor 647 (Invitrogen, T23366; 25 µg/ml), donkey anti-mouse Alexa Fluor 488 (Jackson ImmunoResearch Laboratories, 715-545-151; 1/200) and donkey anti-rabbit Cy3 (Jackson ImmunoResearch Laboratories, 711-165-152; 1/200) were used for immunofluorescence microscopy. LysoTracker Deep Red (1:500,000; Molecular Probes, Invitrogen) and MitoTracker Deep Red FM (1:4000–1:8000; Molecular Probes, Invitrogen) dyes were used for cell imaging.

### Generation of stable cell lines

The lentiviral vector pCDH tagBFP T2A myc-BirA*-Bax used as a base for the BioID lentiviral vectors generated for this study and was a gift from Andrew Gilmore (University of Manchester, UK). Rab4a, Rab11a and Rab25 constructs were expanded by PCR using primers to introduce XhoI/SalI restriction sites, and ligated to replace myc-BirA*-Bax and generate constructs as detailed in Fig. S1A (annotated plasmid maps available at https://doi.org/10.48420/19391300). Lentiviral particles were produced in HEK293T cells via a polyethylenimine (PEI; Sigma-Aldrich)-mediated transfection with pCDH, pM2G (gift from Dr Andrew Gilmore, University of Manchester, UK) and psPAX2 (gift from Dr Andrew Gilmore, University of Manchester, UK) plasmids for 72 h before the medium containing viral particles was collected and subjected to centrifugation for 4 min at 180 ***g*** and filtration through a 0.45 μm filter (Starlab). A2780 cells were resuspended in the filtered virus-containing medium before plating, and the virus was left on cells for 24 h, after which it was removed and replaced with normal growth medium.

TagBFP-expressing cells were selected via fluorescence-activated cell sorting (FACS). A2780 cells were resuspended in Ham's F12 medium (Sigma-Aldrich) supplemented with 25 mM HEPES (Sigma-Aldrich) and 1% antibiotic-antimycotic (Sigma-Aldrich), and filtered through a 50 μm filter (Filcon, BD Biosciences). Cells were sorted using an LSR Fortessa cell analyser and BD FACSDiva 8 software (BD Biosciences) based on tagBFP expression.

### Proximity labelling

Cells expressing mycBirA* fusion protein constructs were plated onto tissue culture plates at a density to ensure sub-confluency and space for cell motility the following day. More than 4 h after cells were plated, 1 μM biotin (Sigma-Aldrich) was added to cell culture medium, and cells were incubated in the presence of biotin for 16 h. BioID cell lysis was carried out at room temperature using a protocol adapted from Roux et al. (2012). Briefly, cells were washed in PBS before addition of the BioID lysis buffer [50 mM Tris pH 7.4, 500 mM NaCl, 0.4% SDS, 5 mM EDTA, 1 mM dithiothreitol (DTT), supplemented with the protease inhibitors: 100 μg/ml leupeptin (Sigma-Aldrich), 100 μg/ml aprotinin (Sigma-Aldrich), 0.5 mM AEBSF (Calbiochem), 500 μM ALLN (Calbiochem) and 50 μM PD150606 (Calbiochem)]. Cell lysates were collected using a cell scraper and subjected to needle lysis. Triton X-100 (Sigma-Aldrich) was added to the cell lysates to a final concentration of 2% before needle lysis and addition of an equal volume of 50 mM Tris pH 7.4. Lysates were clarified by centrifugation (16,000 ***g*** for 10 min at 4°C). Cell lysates were incubated with 15 μl of MagReSyn Streptavidin microspheres (per 10 cm plate; ReSyn Biosciences) overnight at 4°C with rotation. Beads were washed twice in wash buffer 1 (2% SDS), once in wash buffer 2 (0.1% deoxycholate, 1% Triton X-100, 500 mM NaCl, 1 mM EDTA, 50 mM HEPES pH 7.4) and once in wash buffer 3 (0.5% NP-40, 0.5% deoxycholate, 1 mM EDTA, 10 mM Tris pH 8.1). Bound proteins were eluted by the addition of 40 μl of 2× sample buffer saturated with biotin (250 mM Tris-HCl pH 6.8, 2% SDS, 10% glycerol, 0.2% Bromophenol Blue, 20 mM DTT, with >1 mM biotin) at 70°C for 5 min. Finally, 10–25% of eluted samples were retained for western blot analysis and the remaining samples were used for mass spectrometry analysis.

### Mass spectrometry sample preparation

Eluted proteins from BioID experiments were subjected to SDS-PAGE at 100 V for 8 min. Gels were stained with InstantBlue Protein Stain (Expedeon) for 10 min and subsequently washed in ddH_2_O at 4°C overnight with gentle shaking. The entire complement of proteins in the ‘gel-top’ protein band were excised and subjected to in-gel digestion as part of an optimised method to prevent contamination of samples with streptavidin peptides (which are released by harsh elution in our hands). Gel pieces were transferred to a 96-well perforated plate containing ddH_2_O and then centrifuged at 230 ***g*** for 1 min to remove liquid. Gel pieces were de-stained sequentially using 50% acetonitrile (ACN)/50% 25 mM NH_4_HCO_3_, followed by 100% ACN. De-stained gel pieces were dried using a vacuum centrifuge and proteins reduced (10 mM DTT in 25 mM NH_4_HCO_3_) and then alkylated (55 mM iodoacetamide in 25 mM NH_4_HCO_3_) in the absence of light. Gel pieces were washed alternately in 25 mM NH_4_HCO_3_ and 100% ACN, dried using a vacuum centrifuge, and digested with trypsin (1.25 ng/μl in 25 mM NH_4_HCO_3_) overnight. Peptides were collected in 96-well collection plates by centrifugation at 230 ***g*** for 1 min, washed in 99.8% ACN/0.2% formic acid (FA), then in 50% ACN/0.1% FA. Peptide solutions were dried to completion using a vacuum centrifuge and resuspended in 5% ACN/0.1% FA. Desalting of peptides was carried out using POROS Oligo R3 beads (Thermo Fisher Scientific). Beads were washed with 50% ACN, then 0.1% FA, and peptides eluted twice with 50% ACN/0.1% FA. Peptide solutions were dried to completion using a vacuum centrifuge and resuspended in 20 μl 5% ACN/0.1% FA for mass spectrometry analysis.

### Mass spectrometry analysis

Peptides were analysed using liquid chromatography tandem mass spectrometry (LC-MS/MS) using an Orbitrap Elite mass spectrometer (Thermo Fisher Scientific) in the Biological Mass Spectrometry core facility, University of Manchester. Peptides were automatically selected for fragmentation by data-dependent analysis. Four biological replicates of each sample were analysed in one batch on the same day in the following order: mycBirA*–Rab25, mycBirA*–Rab4a, mycBirA*–Rab11a and myc-BirA* only, with all biological replicates of each bait analysed consecutively before replicates of the next bait were analysed. All samples were analysed using 2 h runs and the LC column was equilibrated with a pooled sample (consisting of an equal mix of all samples) between each set of baits.

For MS1 intensity-based analysis, data were analysed using MaxQuant (version 1.6.2.10; https://www.maxquant.org). Default MaxQuant parameters were used, with fixed modifications of cysteine carbamidomethylation and variable modifications of methionine oxidation, protein N-terminus acetylation and lysine biotinylation. Label-free quantification (LFQ) was selected and match between runs was turned on. Data were searched against a human SwissProt/UniProt database (downloaded March 2018). LFQ intensities from MaxQuant analysis were analysed by SAINTexpress (version 3.6.3; https://saint-apms.sourceforge.net/Main.html) using default parameters.

A range of bioinformatic analyses were performed on the mass spectrometry data. PPI network visualisation was performed using Cytoscape (https://cytoscape.org/), with proteins mapped onto the *Homo sapiens* Biological General Repository for Interaction Dataset (BioGRID) 3.4.162 database (https://downloads.thebiogrid.org/BioGRID/Release-Archive/BIOGRID-3.4.162/). Proportional Venn diagrams were generated using an online tool (http://eulerr.co/). Dot plots of the high-confidence proximal proteins identified to each Rab GTPase were generated by ProHits-viz (Knight et al., 2017) (https://prohits-viz.lunenfeld.ca/) from SAINTexpress output data. GO analysis was performed using clusterProfiler (Yu et al., 2012) in R. Principal component analysis (using prcompf base package), pairwise comparisons and the visualisation of biotinylated peptides were all performed in R and plotted using ggplot2 (https://ggplot2.tidyverse.org/).

### Immunofluorescence

For staining with anti-myc (9E10, Fig. 1) antibody, cells were fixed in −20°C methanol for 10 min at −20°C followed by −20°C acetone for 1 min at room temperature. For all other antibody staining procedures (including anti-myc 9B11, Fig. S1), cells were fixed in 4% (w/v) paraformaldehyde (Sigma-Aldrich) for 15 min at room temperature, before permeabilisation with 0.2% Triton X-100 (Sigma-Aldrich) in PBS− (Dulbecco's phosphate-buffered saline without CaCl_2_ and MgCl_2_; Sigma-Aldrich) for 5 min at room temperature. For transferrin internalisation experiments, transferrin Alexa Fluor 647 was added to live cells for the indicated time points and washed three times with PBS- on ice prior to fixation.

Fixed cells were blocked in 1% (w/v) heat-denatured bovine serum albumin (BSA) (Sigma-Aldrich) in PBS− with 10% (v/v) FBS and incubated with primary and secondary antibodies diluted in 1% heat-denatured BSA in PBS− for 1 h at room temperature. Cells were washed five times with PBS and once with ddH_2_O before mounting with either ProLong Gold Antifade Mountant (Molecular Probes, Invitrogen) or ProLong Diamond Antifade Mountant (Molecular Probes, Invitrogen). Images were acquired using a Leica TCS SP8 AOBS inverted confocal microscope using a 63× or 100× APO objective, using hybrid detectors with the appropriate detection mirror settings and sequential image collection, capturing *z*-stacks using LAS X software (Leica).

### Knock sideways imaging and analysis

Cells were nucleofected (VCA-1002, Lonza) with the required amount of plasmid DNA using programme A-23 (Lonza). For live-cell experiments, the following amounts of plasmid DNA were used; 1 μg Mito-mCh(K70N)-FRB (gift from Steve Royle, University of Warwick, UK), 3 μg mCh-Rab11FIP5 (gift from Andrew Lindsay, University College Cork) and 1 μg of the required GFP-FKBP plasmid, to achieve optimal protein expression. Rab11a, Rab4 and Rab25 constructs were introduced into the GFP-FKBP-C1 plasmid (gift from Steve Royle, University of Warwick) by PCR.

Cells were nucleofected as indicated and imaged after 24 h. The cell growth medium was replaced with Opti-Klear Live Cell Imaging Buffer (Tebubio) supplemented with 10% FBS and MitoTracker Deep Red FM (1:8000) for at least 30 min before image acquisition. Cells were imaged using 3i spinning-disk confocal microscopy. Single-section confocal images were collected using a CSU-X1 spinning disc (Yokogawa) on a Zeiss Axio-Observer Z1 microscope with a 63×/1.46 α Plan-Apochromat objective, a Prime 95B Scientific CMOS camera (Photometrics) and motorised *xyz* stage (Applied Scientific Instrumentation). Lasers were controlled using an acousto-optic tunable filter (AOTF) through the laser stack (Intelligent Imaging Innovations, 3i), allowing both rapid ‘shuttering’ of the laser and attenuation of the laser power. Slidebook software (3i) was used to capture images of approximately ten positions per condition, every 5 min for 1 h. Rapamycin (Sigma-Aldrich) to a final concentration of 200 nM was added to cells on the microscope immediately after the first image was acquired to stimulate the rerouting of GFP–FKBP constructs to the mitochondria.

For the knock-sideways fixed approach, we simplified nucleofection by using 2 μg pMito-iRFP670-FRB (Gemperle et al., 2022) with 1 μg of the required GFP–FKBP. The following day, rapamycin was added to the cell growth medium at a final concentration of 200 nM for 4 h. Cells were then fixed and stained for the relevant candidate protein (described above), which was detected by a Cy3 secondary antibody and imaged using Decon Vision deconvolution microscopy. Images were acquired using an Olympus IX83 inverted microscope using Lumencor LED excitation, with a 60×/1.42 Plan Apo objective and a Retiga R6 (Q-Imaging) CCD camera. Metamorph software (Molecular Devices) was used to capture *z*-stacks, and these were then deconvolved using the Huygens Pro software (Scientific Volume Imaging) with default settings.

All images were processed using ImageJ, relocalisation of GFP–FKBP–Rab proteins and mCherry–Rab11FIP5 to mitochondria was analysed using the Colocalization finder plugin and scored based on Pearson's correlation coefficient (no thresholding, noise subtraction using the ScatterPlot function; https://imagej.nih.gov/ij/plugins/colocalization-finder.html). Knock-sideways relocalisation dynamics were corrected for signal crosstalk, normalised to a scale of 0–1, fitted with non-linear fit using GraphPad Prism, and the T_1/2_ was determined as the time necessary to relocalise 50% of the total GFP signal (equilibrium) to mitochondria. For fixed deconvoluted images, a mitochondrial mask was created using a custom-made script (available at https://doi.org/10.48420/19391597) and the mean pixel intensity of the candidate protein was compared between that in mitochondria and that in the cell body.

### siRNA-mediated protein depletion

Lipofection (Lipofectamine 2000, Invitrogen) or CombiMag-mediated magnetofection (OZ Biosciences) were used to introduce siRNA into A2780, BT20 and MDA-MB-231 cells, according to the manufacturers’ instructions.

The following siRNAs were used: CLINT1 siRNA_1, 5′-CAGGCTTCGTGAAGAGCGAAA-3′ (SI04178748, Hs_CLINT1_1); CLINT1 siRNA_3, 5′-ATGGTAAGGATCAAGGTATAA-3′ (SI04230926, Hs_CLINT1_3); CRACR2A siRNA_1, 5′-TACCGTGTGACGGAGAGTCTA-3′ (SI04218130, Hs_EFCAB4A_1); CRACR2A siRNA_4, 5′-AAGAAGGAGGAACCTCATTTA-3′ (SI04322682, Hs_EFCAB4B_4); PI4KB siRNA_5, 5′-TCGGCTGATAGTGGCATGATT-3′ (SI00605850, Hs_PIK4CB_5); PI4KB siRNA_6, 5′-CGACATGTTCAACTACTATAA-3′ (SI02660077, Hs_PIK4CB_6); SH3BP5L siRNA_2, 5′-ATGCACAACGCTGCTCGAGAA-3′ (SI04172315, Hs_SH3BP5L_2); and SH3BP5L siRNA_3, 5′-CACGTCAGTCTGGACGGCCAA-3′ (SI04192993, Hs_SH3BP5L_3). Silencer select siRNA reagents were purchased from Invitrogen: negative control (NS) (4390843), Rab4a siRNA #1 (4390824, ID: s11675) and #2 (4390824, ID: s11676), Rab4b siRNA #1 (4390824, ID: s28800) and #2 (4390824, ID: s28802), Rab11a siRNA #1 (4390824, ID: s16702) and #2 (4390824, ID: s16703), Rab11b siRNA #1 (4390824, ID: s17648) and #2 (4390824, ID: s17647), and Rab25 siRNA #1 (4392420, ID: s32701) and #2 (4392420, ID: s32702).

### RNA extraction and reverse-transcription quantitative PCR

RNA extraction and reverse-transcription quantitative PCR were performed as previously described (Gemperle et al., 2022). The cycling conditions were as follows: 50°C for 2 min, 95°C for 2 min, followed by 35 cycles at 95°C for 15 s, 53°C for 15 s, and 72°C for 60 s. Amplification and melt-curve analysis was carried out using an AriaMx real-time PCR system (Agilent) and Agilent Aria 1.8 software. Relative gene expression was determined using the ΔΔCt method, in which the expression of genes of interest was normalised to that of *GAPDH* per sample and compared relative to a control sample. The following primers were used: GAPDH FW, 5′-AGGTGAAGGTCGGAGTCAAC-3′, and RV, 5′-CCATGTAGTTGAGGTCAATGAAG-3′ (Integrated DNA Technologies); Rab4a FW, 5′-ACTAGCACTAGGGATTCTGG-3′, and RV, 5′-AGAATGTGTTTTCTAGCAGG-3′ (Merck); Rab4b FW, 5′-ACGACTTCCTCTTCAAATTC-3′, and RV, 5′-TCTGTAGCTTCACAGTCTTC-3′ (Merck); Rab11a FW, 5′-ACATCAGCATATTATCGTGG-3′, and RV, 5′-GACGTAGATCACTCTTATTGC-3′ (Merck); Rab11b FW, 5′-GAACAACTTGTCCTTCATCG-3′, and RV, 5′-AGATCTCTGTGAGGATGTTC-3′ (Merck); and Rab25 FW, 5′-AAAACAATGGACTGCTCTTC-3′, and RV, 5′-GATTTCTTTCAGGACAGTCTC-3′ (Merck).

### 3D CDM migration analysis

CDMs were prepared as described previously (Caswell et al., 2007; Cukierman et al., 2001) on either tissue-culture plastic multi-well plates (for long-term time-lapse microscopy) or 35 mm glass-bottomed dishes (MatTek) (for live imaging). Briefly, plates were coated with 0.2% gelatin (Sigma-Aldrich) in PBS before cross-linking with 1% glutaraldehyde (Sigma-Aldrich) in PBS. Plates were washed with PBS and quenched with 1 M glycine (Thermo Fisher Scientific) in PBS before washing and equilibration in DMEM. TIFs were plated onto the prepared plates at a density to ensure confluency the following day. TIFs were grown for 8–10 days, with the medium changed for DMEM supplemented with 25 μg/ml ascorbic acid (Sigma-Aldrich) 24 h after seeding and every 48 h after that. Matrices were denuded of cells with extraction buffer [20 mM NH_4_OH and 0.5% (v/v) Triton X-100 in PBS] and washed twice with PBS+ (Dulbecco's phosphate buffered saline with CaCl_2_ and MgCl_2_, Sigma-Aldrich), incubated with 10 μg/ml DNase I (Roche) and washed three times with PBS+, before A2780 cells were plated at a density of 4×10^4^ cells per well of a six-well plate or 35 mm dish. Cells were allowed to spread and start migrating for at least 4 h before use in experiments.

Images were acquired on an Eclipse Ti inverted microscope (Nikon) using either a 20×/0.45 S Plan Fluor or a 10×/0.3 Plan Fluor objective, and a pE-100 LED (CoolLED) light source. Images were collected using a Retiga R6 (Q-Imaging) camera, and cells were maintained at 37°C and 5% CO_2_ for the duration of imaging. NIS Elements AR.46.00.0 software (Nikon) was used to acquire images of multiple positions per well, every 10 min for 16 h. For cell migration analysis, at least 90 cells (in total) were individually manually tracked per position using the ImageJ MTrackJ plugin, and this was used to calculate the average cell speed and persistence, where persistence is equal to the path length divided by euclidean distance (Meijering et al., 2012).

### Live-cell imaging

Cells were nucleofected with mCherry-CRACR2A-L (Addgene #79593) and EGFP–Rab25 (Caswell et al., 2007) as indicated and seeded onto 35 mm glass-bottomed CDMs 24 h later. Cell growth medium was replaced with Opti-Klear Live Cell Imaging Buffer (Tebubio) supplemented with 10% FBS at least 30 min before image acquisition. Cells were incubated with LysoTracker Deep Red (1:500000) diluted in Opti-Klear for at least 30 min before image acquisition and maintained at 37°C during imaging.

Live-cell imaging was carried out using spinning-disk confocal microscopy (3i Marianis). Single-plane images were collected using a CSU-X1 spinning disc (Yokogawa) on a Zeiss Axio-Observer Z1 microscope with a 63×/1.46 α Plan-Apochromat objective and a Prime 95B Scientific CMOS camera (Photometrics). Lasers were controlled using an AOTF through the laser stack (Intelligent Imaging Innovations, 3i) allowing both rapid ‘shuttering’ of the laser and attenuation of the laser power. Slidebook software (3i) was used to capture images of cells every 30 s for 10 min. Images were processed and analysed using ImageJ.

### Analysis of focal adhesions and cell protrusions

Images were acquired using spinning-disk confocal microscopy (3i Marianism as above, *z*-stacks of fixed cells captured). Deconvolution was carried out using Huygens Pro software prior to image analysis. Images were further processed in ImageJ using background subtraction (rolling ball radius of 10 pixels) and Gaussian blur (sigma radius of 1 pixel) prior to adhesion analysis. For quantification of paxillin-positive adhesions, semi-automated thresholding was applied to create a mask by which adhesion number per cell could be measured. One focus plane was chosen per cell stack and thresholded particles with a size below 0.3 µm^2^ were removed. For analysis of protrusions, cells in the CDM were fixed and stained for F-actin using Alexa Fluor 647-phalloidin. Images were captured using a 63× objective and 3i Marianis spinning-disk confocal microscope, and analysed in ImageJ. For protrusion width, the width measurement was made for the pseudopod that had extended furthest from the cell body 2–5 µm from the furthest extent of protrusion (width being orthogonal to the direction of protrusion). To score protrusions as bearing filopodia or lamellipodia, the structure of F-actin was defined as ‘filopodial’ where protrusions were tipped by high-intensity thin needle like projections, or ‘lamellipodial’ where protrusions were tipped with veils of F-actin that appeared wider and less intense than filopodia.

### GST pull-down assays

GST pull-downs were performed as described previously (Caswell et al., 2007). Briefly, His–Rab25 was expressed in *Escherichia coli* strain BL-21 and purified by sequential Ni-affinity and size-exclusion chromatography. Rab25 was loaded with GTPγS by incubation in PBS containing 4.3 mM EDTA and 0.11 mM GTPγS (Sigma-Aldrich) at room temperature for 1 h, before addition of MgCl_2_ to 6.5 mM. Glutathione–agarose beads (Thermo Fisher Scientific) were bound to bacterially expressed purified GST–β1 integrin cytoplasmic tail truncations, washed extensively and incubated with His–Rab25 GTPγS for 2 h at 4°C. After extensive washing to remove unbound proteins, bound proteins were eluted by boiling in Laemmli sample buffer and proteins resolved by SDS-PAGE, followed by Coomassie Blue staining or western blotting.

### Statistics

Data were tested for normality and one-way ANOVA with Tukey’s or Holm–Sidak post hoc test used for multiple comparisons as indicated in the legends. Where data were not normally distributed, Kruskal–Wallis test with Dunn's multiple comparison was used. All statistical analysis was performed with GraphPad Prism software, where ***P<0.001, **P<0.01 and *P<0.05. Data represent at least three independent experiments; *n*- and *P*-values are described in the relevant figure legends.

## Supplementary Material

10.1242/joces.260468_sup1 Supplementary information
