# Supplementary material for: Proximity labelling identifies pro-migratory endocytic recycling cargo and machinery of the Rab4 and Rab11 families
Source: J Cell Sci. 2023 Jun 23;136(12):jcs260468. doi: 10.1242/jcs.260468 (PMC10323252; doi:10.1242/jcs.260468)
Supplement: Supplementary information [file joces-136-260468-s1.pdf]

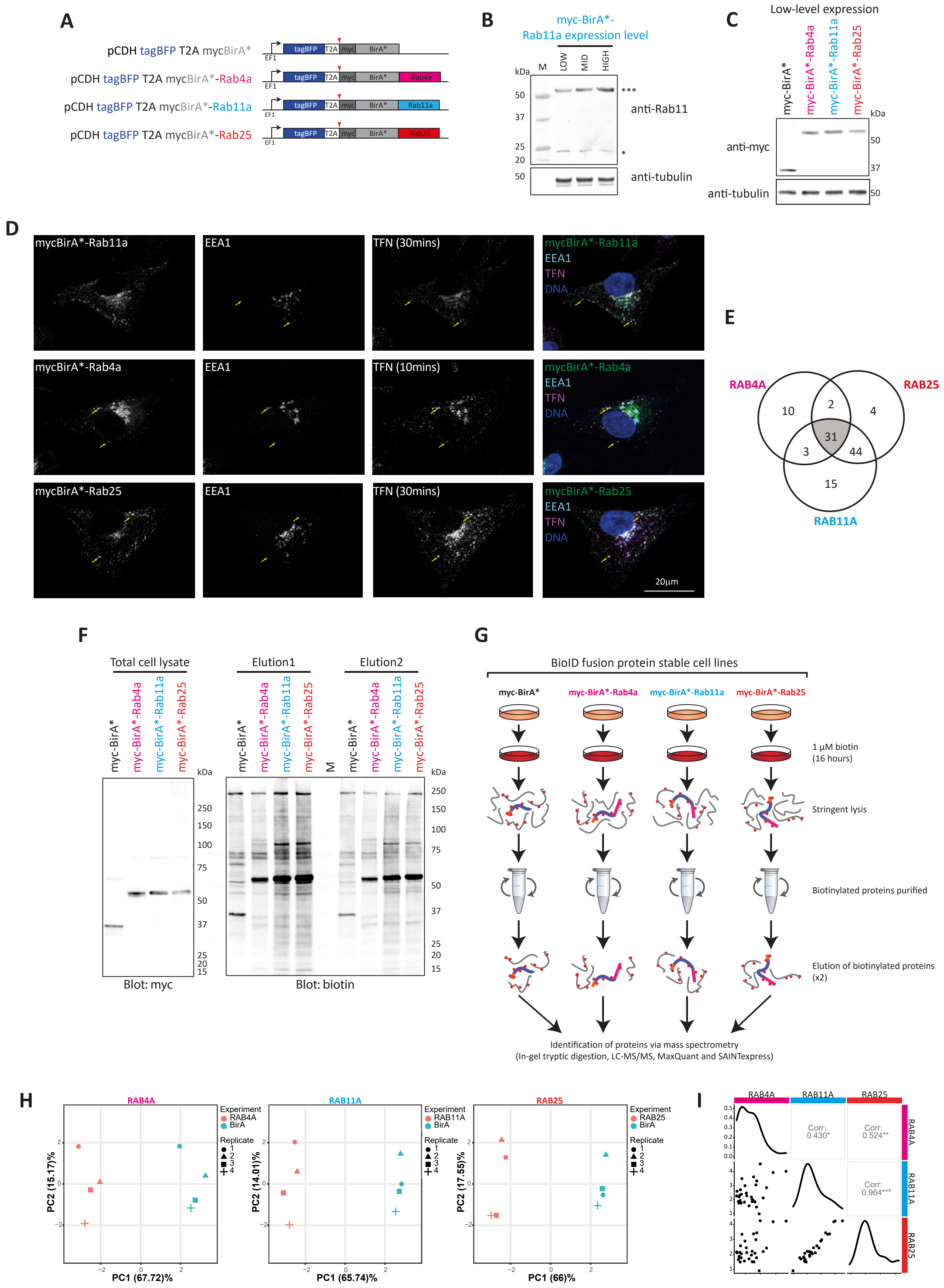

Journal of Cell Science • Supplementary information

Fig. S1. Rab4a/11a/25 proximity biotinylation.

(A). Schematic of the lentiviral BioID constructs used to generate stable cell lines. Co-expression occurs before co-translational cleavage of the T2A peptide, enabling the separate expression of tagBFP and the fusion protein. **(B).** Expression of myc-BirA*-Rab11a in stable cell lines was confirmed by western blotting using a Rab11 antibody (upper panel), and α-tubulin as a loading control (lower panel). *, endogenous Rab11; ***, myc-BirA*-Rab11a. Molecular weight marker, M, is shown in kDa. **(C).** Expression of BirA* fusion proteins in stable cell lines was confirmed by western blotting using a myc antibody (myc, upper panel), and α-tubulin as a loading control (lower panel). **(D).** A2780 cells stably expressing low levels of mycBirA* fusion proteins were labelled with Transferrin Alexa Fluor™ 647 (25μg/ml) for 10mins or 30mins to label early or recycling endosomes respectively. Cells were fixed and stained for myc fusion proteins alongside EEA1, and imaged by scanning confocal microscopy. Representative images are shown, scale bar=20μm. **(E).** Venn diagram showing the overlap of high-confidence proximal proteins (BFDR≤0.05) identified for Rab4a, Rab11a and Rab25 using an optimised BioID protocol (see S1G) that was carried out with four biological replicates (n=4). Samples were analysed by mass spectrometry before data analysis by MaxQuant and SAINT express to identify high-confidence Rab GTPase proximal proteins. **(F)**. A2780 cells stably expressing BirA* fusion proteins were plated for 7-8 h and supplemental biotin (1μM) added for 16 h as indicated. Cells were lysed and biotinylated proteins were affinity purified using MagReSyn® streptavidin microspheres, with two subsequent elutions carried out in 2x sample buffer saturated with biotin, for 5 min at 70 °C. A proportion of resultant samples (10%) were analysed by western blot, and a representative blot from one of the four replicates is shown. Biotinylated proteins detected by Alexa800-streptavidin (biotin). Molecular weight marker, M, is shown in kDa. **(G).** Experimental pipeline. Identification of enriched proteins was carried out by LC-MS/MS, followed by analysis by MaxQuant and SAINTexpress. **(H).** Principle component analysis of label-free quantification intensities (from MaxQuant) for the identified affinity-purified proteins. The analyses were carried out on all replicates of the BirA*-only control and all replicates of each BirA*-Rab GTPase fusion protein in turn. **(I).** Pairwise comparison of the Rab4a, Rab11a and Rab25 fold changes (log_2FoldChange) over myc-BirA* of the 31 high-confidence proximal proteins shared by all 3 Rab GTPases, Pearson correlation values are shown.

Journal of Cell Science • Supplementary information

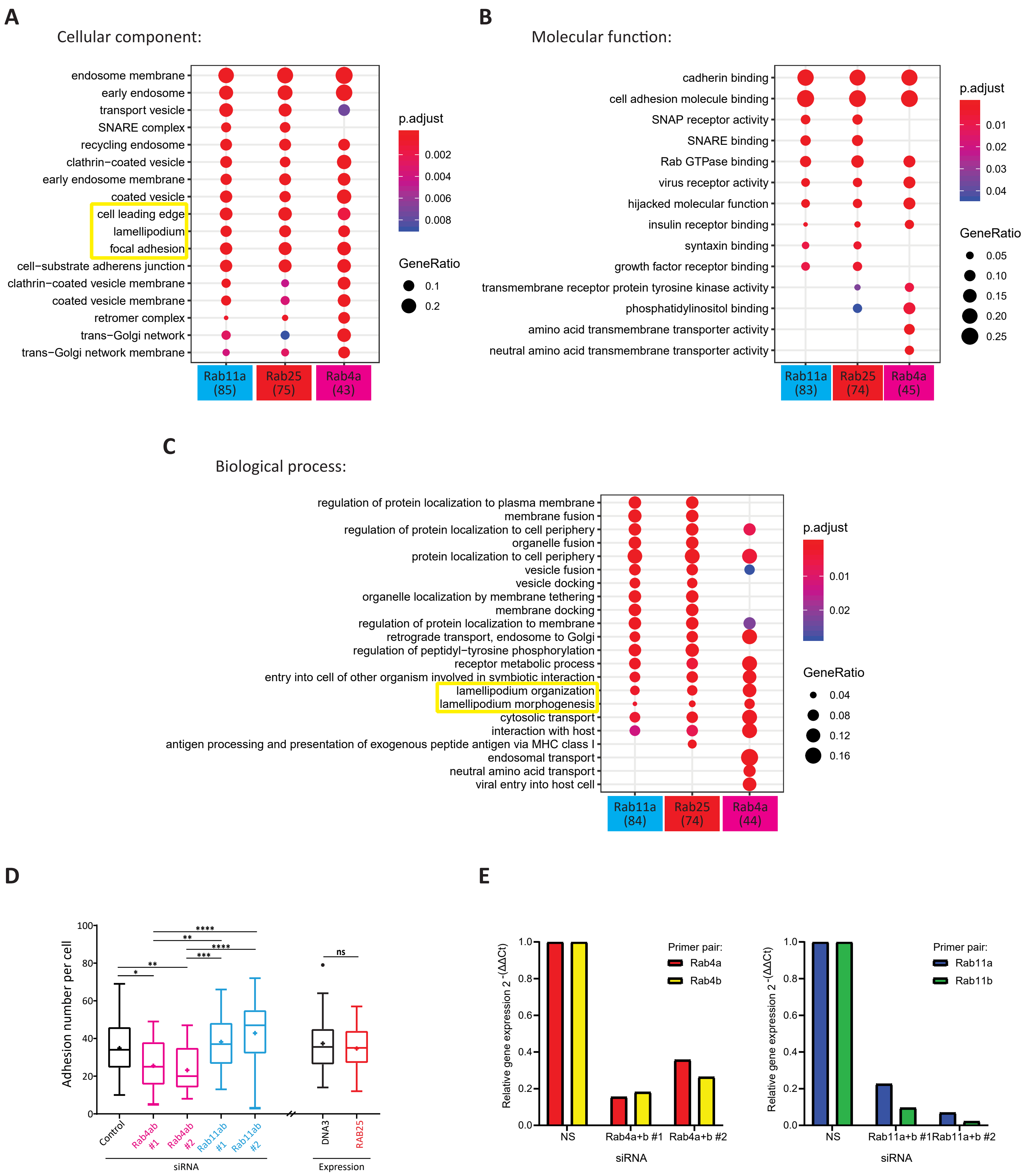

Journal of Cell Science • Supplementary information

Fig. S2. Gene ontology analysis of high-confidence Rab GTPase proximal proteins.
GO analysis of the high-confidence proximal proteins for Rab4a, Rab11a and Rab25 was carried out. The top 10 enriched GO terms under the stated categories are displayed for each bait (p-value ≤ 0.05), nodes coloured according to p.adjust, adjusted p-value. Number of proteins recognised per bait in brackets; GeneRatio, proportion of total proteins identified in each GO term. **(A).** Cellular component GO category. **(B).** Molecular function GO category. **(C).** Biological process GO category. **(D).** A2780-DNA3 cells and A2780-Rab25 (depleted of Rab4a and Rab4b or Rab11a and Rab11b by siRNA where appropriate) were seeded onto glass coverslips for 16 hours before fixation, staining for F-actin and paxillin, and imaged by confocal microscopy. Number of adhesions was quantified using ImageJ . Statistical analysis with ANOVA/Kruskal-Wallis post-hoc test (Control versus Rab4ab/Rab11ab) or unpaired Student's T-test ((DNA3 versus Rab25); *$p<0.05$; **$p<0.01$; ****$p<0.001$). $n>29$ cells/condition from at least 3 independent experiments. **(E).** Knockdown of Rab4a and Rab4b or Rab11a and Rab11b using two different siRNA oligos was confirmed using qPCR. Data representative of 3 independent experiments shown.

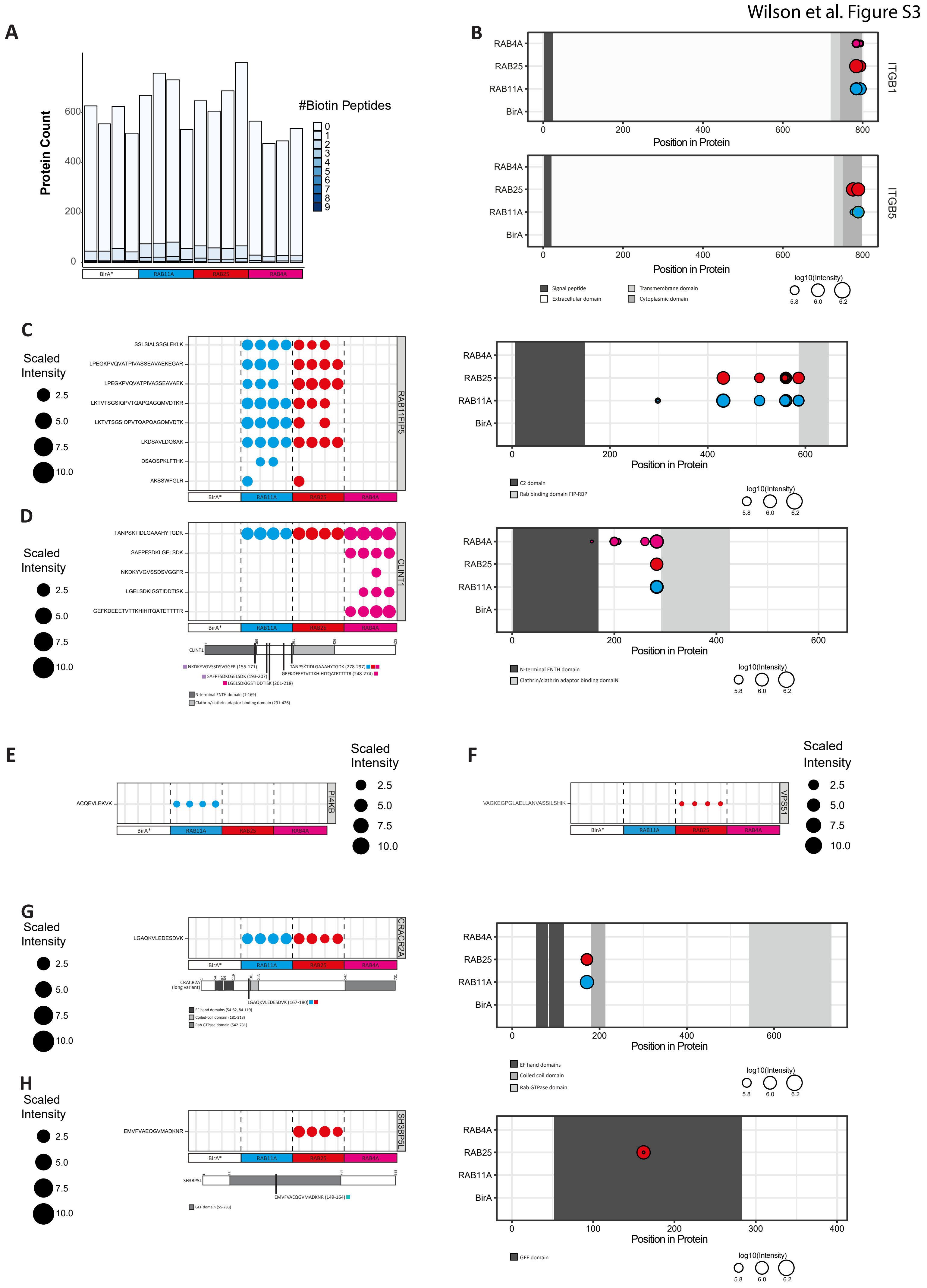

Fig. S3. Mapping biotin modifications in prey proteins.

(A). Numbers of biotinylated peptides identified for each protein group in each replicate, colour coded according to the number of biotinylated peptides identified for each. **(B-H).** Biotin modifications within prey proteins were mapped according to peptide and/or position in the prey protein.

Journal of Cell Science • Supplementary information

A

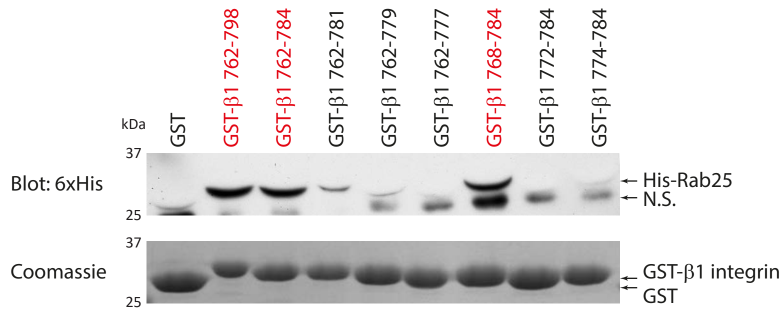


```
GST-b1 762-798 EFAKFEKEKMNAKWDTGENPIYKSAVTTVVNPKYEGK
GST-b1 762-784 EFAKFEKEKMNAKWDTGENPIYK
GST-b1 762-781 EFAKFEKEKMNAKWDTGENP
GST-b1 762-779 EFAKFEKEKMNAKWDTGE
GST-b1 762-777 EFAKFEKEKMNAKWDT
GST-b1 768-784       KEKMNAKWDTGENPIYK
GST-b1 772-784           NAKWDTGENPIYK
GST-b1 774-784             KWDTGENPIYK
Biot. peptide 1             WDTGENPIYKSAVTTVVNPK
Biot. peptide 2                       SAVTTVVNPKYEGK
```

B

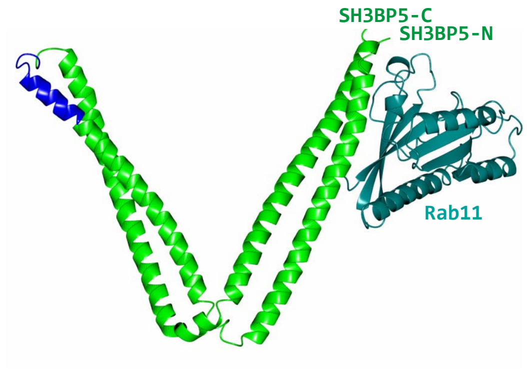


```
SH3BP5    ---------GMDAALKRSRSEEPAEILPPARDEEE-----EEEEGMEQGLEEEEEVDPRI   46
SH3BP5L   MAELRQVPGGRETPQGELRPEVVEDEVPRSPVAEEPGGGGSSSSEAKLSPREEEEELDPRI  60

SH3BP5    QGELEKLNQSTDDINRRETELEDARQKFRSVLVEATVKLDELVKKIGKAVEDSKPYWEAR   106
SH3BP5L   QEELEHLNQASEEINQVELQLDEARTTYRRILQESARKLNTQGSHLGSCIEKARPYYEAR   120

SH3BP5    RVARQAQLEAQKATQDFQRATEVLRAAKETISLAEQRLLEDDKRQFDSAWQEMLNHATQR   166
SH3BP5L   RLAKEAQQETQKAALRYERAVSMHNAAREMVFVAEQGVMA-DKNRLDPTWQEMLNHATCK   179

SH3BP5    VMEAEQTKTRSELVHKETAARYNAAMGRMRQLEKKLKRAINKSKPYFELKAKYYVQLEQL   226
SH3BP5L   VNEAEEERLRGEREHQRVTRLCQQAEARVQALQKTLRRAIGKSRPYFELKAQFSQILEEH   239

SH3BP5    KKTVDDLQAKLTLAKGEYKMALKNLEMISDEIHERRRSSA    266
SH3BP5L   KAKVTELEQQVAQAKTRYSVALRNLEQISEQIHARRRGGL    279
```

Fig. S4. BioID mediated biotinylation provides insight into bait: prey interactions

(A). His-Rab25 loaded with GTPγS was incubated with glutathione sepharose beads pre-coated with GST or GST-beta1 integrin cytoplasmic tail truncations before extensive washing. GST pulldowns were evaluated by SDS-PAGE and western blotting (for His-Rab25) or Coomassie brilliant blue staining (to identify GST protein loading). (B). Pymol model showing position of Rab11 and SH3BP5 binding interface (PDB: 6DJL), with the position of the SH3BP5L biotinylated peptide identified by Rab25 in blue. Sequence comparison of SH3BP5 and SH3BP5L showing the Rab11 binding site is conserved as it contains the contact residues identified by Jenkins et al., at both the N and C-terminus that form the coiled-coil required for recognition (green bold and highlighted yellow). The peptide biotinylated by Rab25 is shown in blue underlined.

Journal of Cell Science • Supplementary information

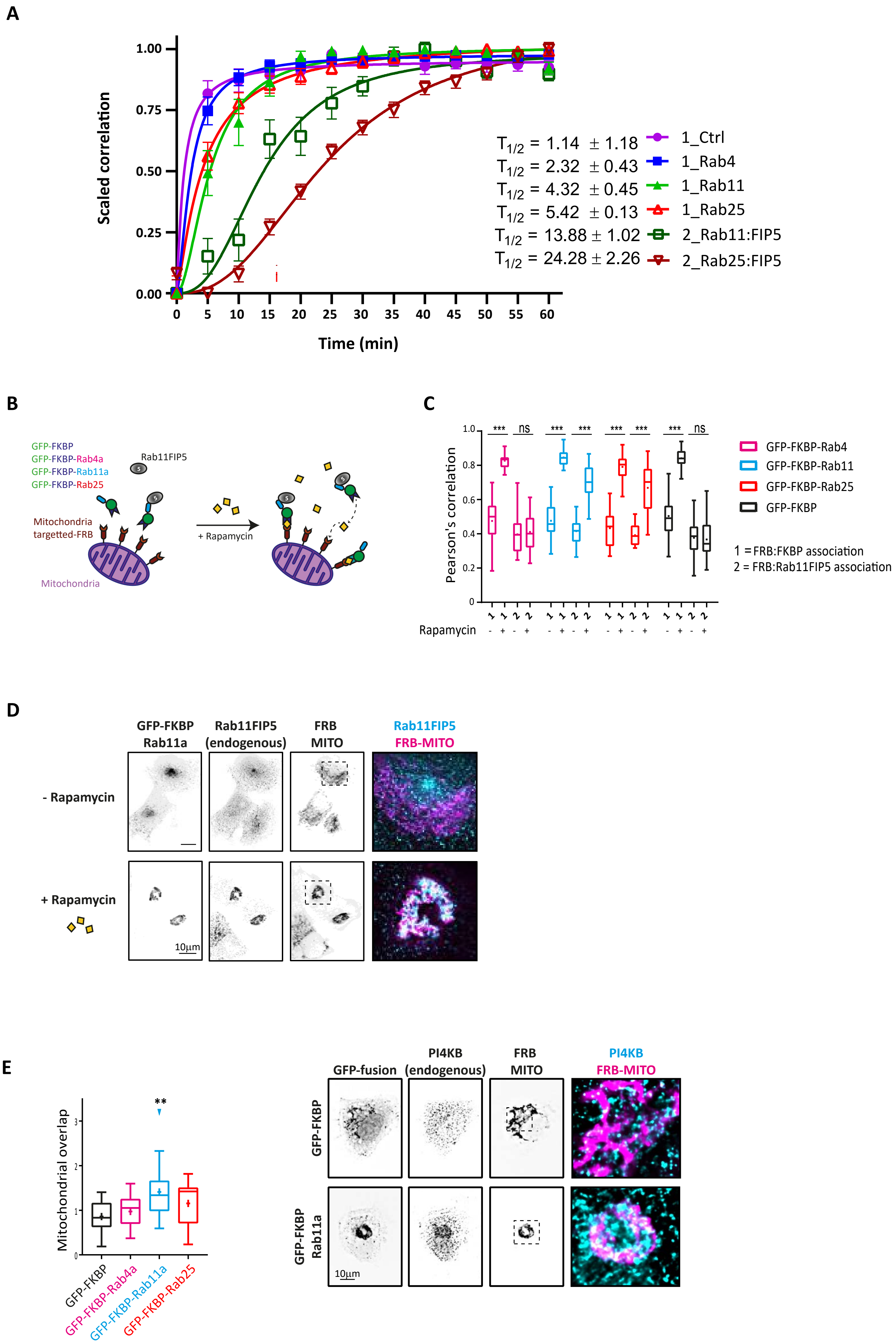

Journal of Cell Science • Supplementary information

Fig. S5. Knock-sideways validation of proximity labelling-identified preys.

(A). Quantification of the redistribution of GFP-tagged proteins and corresponding localisation of mCherry-Rab11-FIP5 in live A2780 cells expressing iRFP670-FRB and GFP-FKBP or GFP-FKBP-Rab4a/11a/25 and mCherry-Rab11-FIP5 (for images see Figure 5C, see methods). **(B).** Schematic illustration of knock-sideways, where mitochondria-targeted FRB/rapamycin is used to induce the re-localisation of GFP-FKBP-tagged Rabs and associated protein complexes. A2780 cells expressing iRFP670-FRB and GFP-FKBP-Rabs treated +/- rapamycin (200 nM 4h) were fixed and stained for endogenous Rab11-FIP5 **(C, D)** or PI4KB **(E)**. Re-distribution of GFP-FKBP fusion (1) and mCherry-Rab11-FIP5 (2) were analysed by Pearson's correlation (B; at least 19 cells/ condition; statistical analysis with ANOVA/Holm-Sidak post hoc test; ***p<0.001), representative images from at least 3 independent experiments are show in (C; scale bar=10μm). Redistribution of PI4KB was analysed by quantifying mitochondrial overlap (D; at least 11 cells/condition; statistical analysis with ANOVA/ Holm-Sidak post-hoc test; **p<0.01) representative images from at least 3 independent experiments are shown (scale bar=10μm).

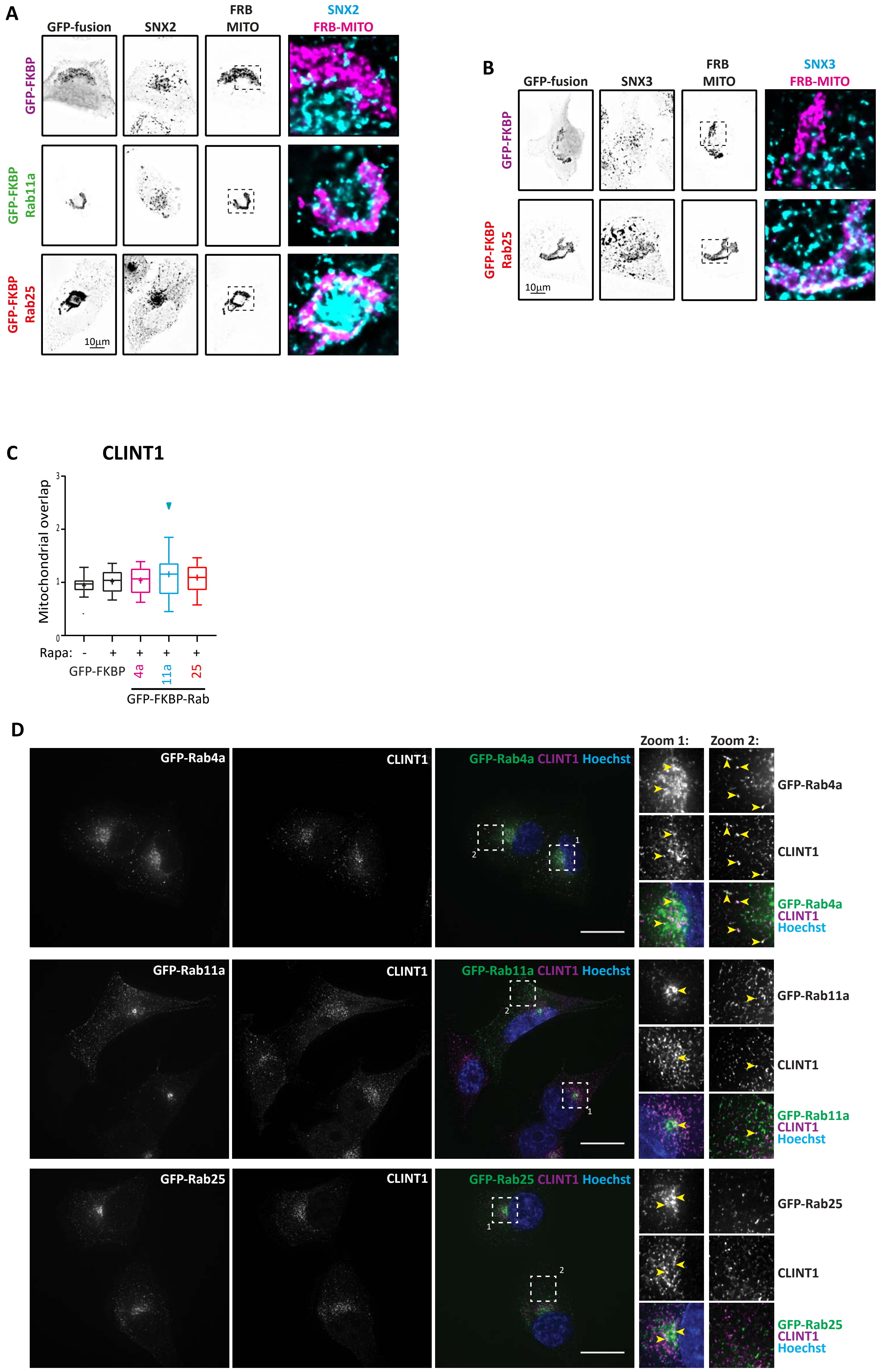

Journal of Cell Science • Supplementary information

Fig. S6. Sorting nexins are selectively recruited to Rab11a and Rab25.

A2780 cells expressing iRFP670-FRB and GFP-FKBP-Rabs treated with rapamycin (200 nM 4h) were fixed and stained for endogenous SNX2 (A) or SNX3 (B). Representative images from at least 3 independent experiments are shown (scale bar=10μm). (C) . A2780 cells expressing iRFP670-FRB and GFP-FKBP-Rabs treated +/- rapamycin (200 nM 4h) were fixed and stained for endogenous CLINT1. Redistribution of CLINT1 was analysed by quantifying mitochondrial overlap (see methods; at least 13 cells/condition; statistical analysis with ANOVA/Tukey post-hoc test;). (D) A2780 cells expressing GFP-Rabs were fixed and stained for CLINT1, and imaged by deconvolution microscopy. Representative images from at least 3 independent experiments are show in (C; scale bar=20μm).

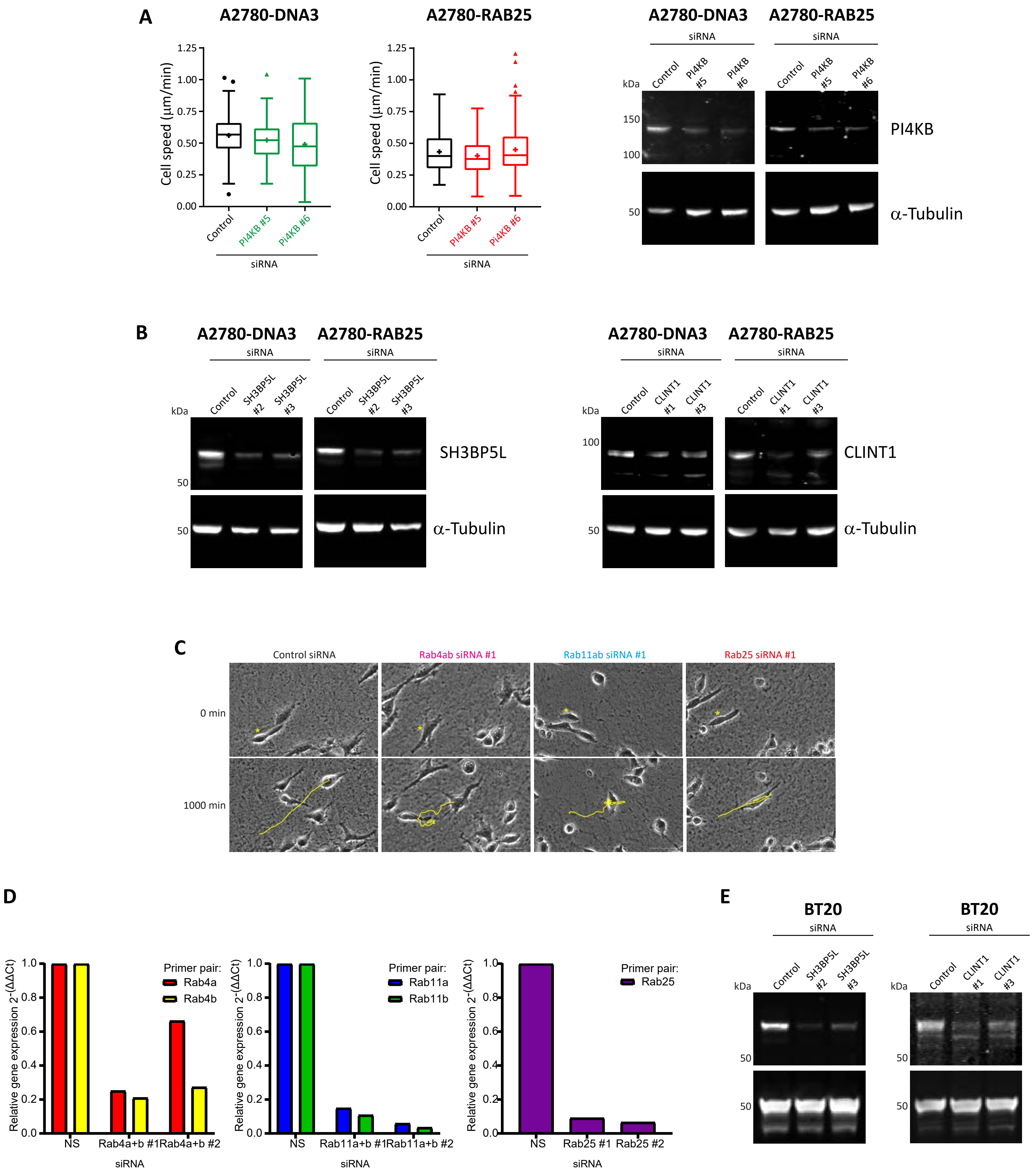

Journal of Cell Science • Supplementary information

Fig. S7. Knockdown efficiency for SH3BP5L and CLINT1

(A). A2780-DNA3 and A2780-Rab25 cells were depleted of PI4KB by siRNA and seeded into cell-derived matrix for 4 hours before migration was analysed by brightfield timelapse imaging for >16 hours. Cell speed was analysed by manual tracking (A; n≥90 cells from 3 independent experiments; statistical analysis with Kruskal-Wallis/Dunn's multiple comparisons test; $**p<0.01$); knockdown analysed by western blot after 48 hours (corresponding to the endpoint of migration analysis). **(B).** A2780-DNA3 and A2780-Rab25 cells were depleted of SH3BP5L or CLINT1 by siRNA and knockdown analysed by western blot after 48 hours (corresponding to the endpoint of migration analysis). Images are representative of 3 independent experiments. **(C).** BT20 cells were depleted of Rab4a and Rab4b, Rab11a and Rab11b, or Rab25 by siRNA and migration analysis performed; representative images from at least 3 independent experiments are shown (scale bars=50μm). **(D).** BT20 cells were depleted of Rab4a and Rab4b, Rab11a and Rab11b, or Rab25 using two different siRNA oligos and knockdown was confirmed using qPCR. Data representative of 3 independent experiments shown. **(E).** BT20 cells were depleted of CLINT1 or SH3BP5L by siRNA (2x transfections) and knockdown analysed by western blot after 6 days (corresponding to the endpoint of migration analysis). Images are representative of 3 independent experiments.

Journal of Cell Science • Supplementary information

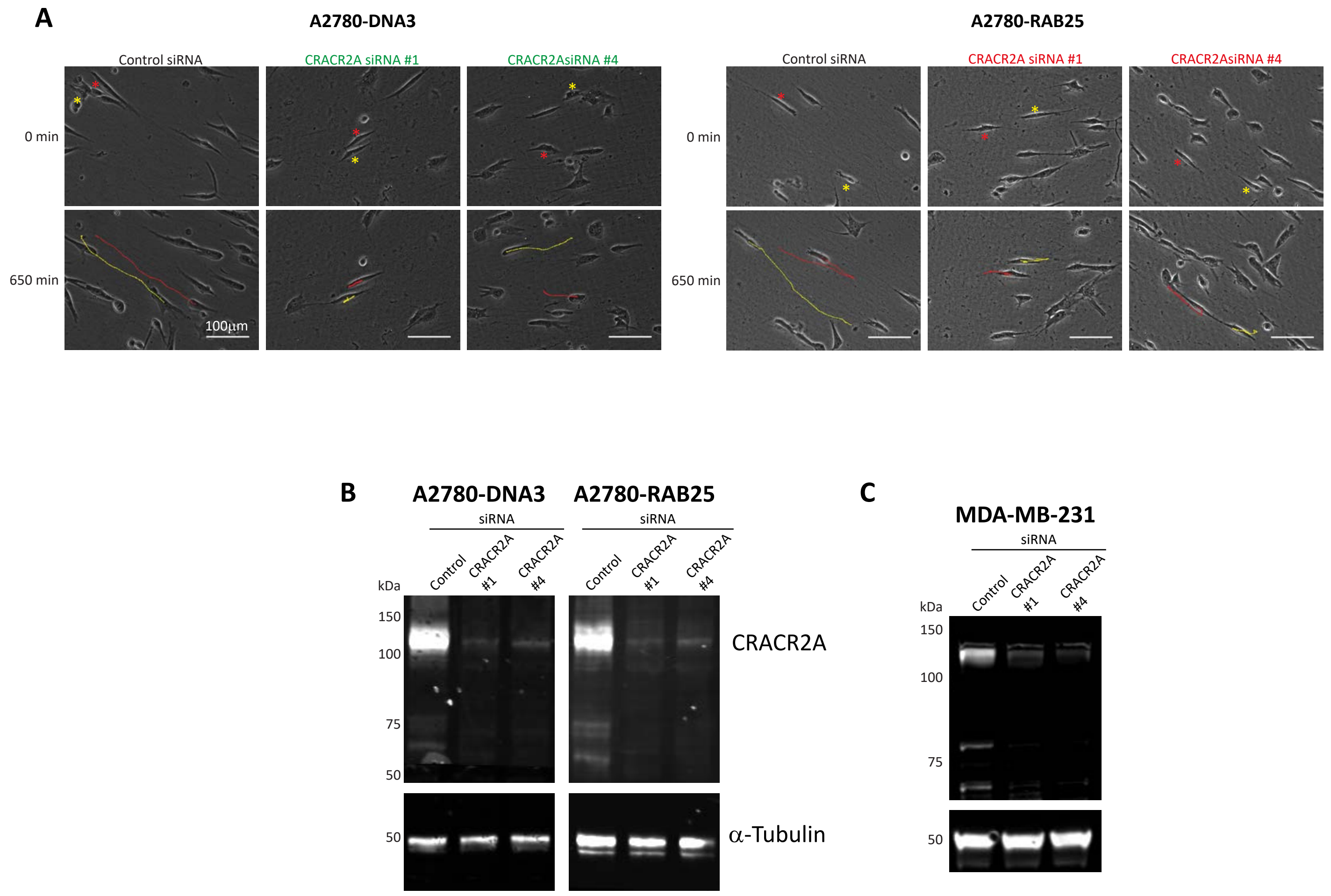

Fig. S8. Recycling machineries required for cell migration in 3D-matrix

(A). A2780-DNA3 and A2780-Rab25 cells were depleted of CRACR2A by siRNA and seeded into cell-derived matrix for 4 hours before migration was analysed by brightfield timelapse imaging for >16 hours. Representative images from at least 3 independent experiments are shown (scale bars=100μm). **(B, C).** A2780-DNA3, A2780-Rab25 or MDA-MB-231 cells were depleted of CRACR2A by siRNA. Knockdown was analysed by western blot after 48 hours (corresponding to the endpoint of migration analysis). Images are representative of 3 independent experiments.

Journal of Cell Science • Supplementary information

Table S1. High confidence Rab proximal proteins identified by BioID

SAINTexpress output for the statistical significance of prey proteins identified by BioID for RAB4A, RAB11A and RAB25.

Click here to download Table S1

Table S2. Longlist Rab proximal proteins identified by BioID

Proteins enriched 2-fold in at least 3 of 4 repeats compared to BirA control. For these proteins the number of unique peptides identified, LFQ intensities in each of four BioID experiments, average LFQ (null values in BirA control were pseudocounted as 0.00001 to give an enrichment ratio) and Rab/BirA enrichment ratio are shown

Click here to download Table S2

Table S3. Peptide modifications identified (including biotinylation).

All biotinylated peptides identified by BioID, in each sample, for RAB4A, RAB11A and RAB25.

Click here to download Table S3

Journal of Cell Science • Supplementary information
