# Supplementary material for: Proximity labelling identifies pro-migratory endocytic recycling cargo and machinery of the Rab4 and Rab11 families
Source: J Cell Sci. 2023 Jun 23;136(12):jcs260468. doi: 10.1242/jcs.260468 (PMC10323252; doi:10.1242/jcs.260468)
Supplement: Supplementary file 1 [file joces-136-260468_review_history.pdf]

Journal of Cell Science

Review COMMONS

Proximity labelling identifies pro-migratory endocytic recycling cargo and machinery of the Rab4 and Rab11 families

Beverley Wilson, Chloe Flett, Jakub Gemperle, Craig Lawless, Matthew Hartshorn, Eleanor Hinde, Tess Harrison, Megan Chastney, Sarah Taylor, Jennifer Allen, Jim C Norman, Thomas T Zacharchenko and Patrick T Caswell
DOI: 10.1242/jcs.260468

Editor: Giampietro Schiavo

Review timeline

Submission to Review Commons:	15 May 2022
Submission to Journal of Cell Science:	28 July 2022
Editorial decision:	1 August 2022
First revision received:	15 May 2023
Accepted:	18 May 2023

Reviewer 1

Evidence, reproducibility and clarity

From methodology and reproducibility point of view it is an excellent manuscript. It is also well-written.

Significance

- This is a manuscript that presents an in-depth analysis of potential interactome and cross-interactome of Rab11a, Rab4a and Rab25 GTPases. BioID and knock-sideway data presented in first half of the manuscript is very interesting and undoubtedly will be of a good use for many laboratories. However, authors tend to over-interpret some of their data, suggesting functional connections between specific Rabs and BioID hits without any additional data. Authors admit themselves that there are some disconnects between BioID and knock-sideways data. Furthermore, BioID measure proximity and not functional connection.

Second half of the manuscript focuses on taking some of the BioID hits and testing whether they are required for mediating cell migration. By itself, it is a great idea since that would provide that functional connection that is missing in the original BioID screen. Unfortunately, the data is limited to few knock-downs without any further analyses of the involvement of these proteins in regulating migration. Consequently, as it stands, this manuscript is essentially a BioID screen with limited insights or validation of specific "hits", thus, does not really lead to any new conclusions about cross-function of Rab11, Rab25 and Rab4 networks.

- Additional comments:

1) There are no blots shown (only boxes) for Figure S1C-D. Data in Figure S1 doe shown that BirA-Rab11a is expressed in similar levels as endogenous Rab11. However, no data supporting similar statement for Rab4 and Rab25 is shown.

2) The presence of specific proteins in BioID does not mean that they either directly bind or regulate particular BirA-Rab. For example, authors state "DENND4C, related to Drosophila Rab11

© 2023. Published by The Company of Biologists under the terms of the Creative Commons Attribution License (https://creativecommons.org/licenses/by/4.0/).

GEF CRAG, was enriched to Rab11a, suggesting that this could be an alternate GEF for Rab11". There is no data supporting such a statement in this manuscript. Actually, DENND4C is better known GEF for Rab10. Rab10 is also known as Rab present in recycling endosomes, thus, could have easily be present in Rab11a-positive recycling endosomes. There are numerous similar statements in the manuscript that implies functional connections between Rabs and BioID "hits" without providing any other functional data.

Authors should not use RCP term to refer to Rab11FIP1 since Rab11FIP1 is its established name and using other names only creates confusion. RCP term was first used to indicate that Rab11FIP1 can bind to both Rab4 and Rab11, the hypothesis that since then was proven to be incorrect.

Reviewer 2

Evidence, reproducibility and clarity

Wilson et al. submitted a paper entitled: "Proximity labelling identifies pro-migratory endocytic recycling cargo and machinery of the Rab4 and Rab11 families". The goal of the paper is to identify new interactors for RAB4/11 and 25 that could be involved in Rab- dependent migration. To do so, they used BioID of the aforementioned 3 Rabs in mesenchymal and migratory ovarian cancer cell line A2780. They validate some of the interactors using the knock-sideways technique and test the requirement of some interactor for migration/invasion in a 3D matrix. This is a very descriptive paper that could benefit from more in-depth mechanistic analysis of fewer candidates.

Major comments:

- For the most part, the data appears of excellent quality and most of the conclusions or interpretations are correct (see below for a few points that can be improved). Some key concepts are missing in the introduction. For example, the concept of GEFs and GAPs only appears later in the experimental section - this should be introduced earlier.

- The description of the BioID data is poorly structured and descriptive, a recurring challenge with big data paper. One suggestion to improve the manuscript would be to exploit the best- known interactions to clearly benchmark the efficiency of the screens. Next the new interactions could be described and figure 2 could be better exploited in that respect (the mentioned complexes could be better drawn etc.). The text could also be more focused on fewer interactors such that it is more digestible for the readers. The major weakness of the manuscript, in my opinion, is the lack of depth in testing functionally some of the uncovered novel interactions.

- Some additional experiments that would be needed to support the claim of novelty in the paper include testing the function of some of the tested interactions. For example, the novel GEF interactions would benefit from biochemical testing in addition to BioID. Likewise, the section on biotinylation and interaction domain mapping is interesting but is, as presented, a theory. Using one interaction to dissect in more details to support this claim is needed.
Alternatively, can the authors demonstrate that this approach can be used to confirmed known protein domains involved in protein-protein interactions of these Rabs? Finally, the authors end their manuscript by screening candidates issued from their BioID which have not been implicated in migration/invasion before. This is somewhat preliminary and fails to provide some depth into the function of one of these potential interactions (domain mapping, knockdown rescue of M or mutants etc.).

- The authors use the knock-sideways technique to validate the strength of their interaction. This is a clever way to validate interaction in cellulo which could be difficult using conventional IP. However, it looks like the expression of FRB-MITO leads to mitochondria fragmentation and aggregation. Is it possible that this cause a bias in their quantification analysis because it becomes difficult to clearly delineate individual mitochondria? In some cases (ex. Fig 5C), the recruitment of the candidate is obvious. However, in other cases (ex. Figure SA) the recruitment to the mitochondria is not very convincing and looks more like the candidates collapse around the aggregated mitochondria. The authors should therefore describe the limitations in more details.

© 2023. Published by The Company of Biologists under the terms of the Creative Commons Attribution License (https://creativecommons.org/licenses/by/4.0/).

Minor comments:

- The authors aim to identify new interactors involved in migration, but they performed the BioID on confluent cells where cell migration is likely limited. Would comparing a BioID performed on confluent cells with one where the cells are sparse enough to migrate possibly interesting to conduct? This could be discussed.

- In Figure 1C, it is difficult to read the name on the candidates. The authors should fit the entire name in the nodes (maybe use an ellipse instead of a circle).

- In Figure 1C and 2 the known interactors could be in a different color emphasize the new potential interactors.

- Figure 4 is very heavy and the images are small making difficult to see the results clearly.

Instead of showing 10 time points per condition, 3 or 4 time point with higher resolution images would have been more appropriate.

- *Methods:' The methods are well described. It is a bit surprising that the BioID samples are run on SDS-PAGE and that bands are cut when on beads digestion is currently done by many lab for this technique.

- *Statistics:* Statistics should be provided for all quantification, not only the one that are significant. For the non-significant, the P-value should be indicated on the figure.

The authors looked at endogenous Rab11 vs BioID-Rab11. Why no do it for the other 2 Rabs. Also, quantification of endo/exo expression should be done.

Significance

- The advance of this work is to expand the potential functional interactome of three Rabs involved in slow recycling of endosomes. Some novel interactions are reported and some screening approaches have been use to reveal functional ones (this could be improved).

- This work is potentially important and part of the priorities in the field to ascribe the overlapping and specific interactions/functions of Rab subfamilies. Similar work has been done for Rho proteins and selected Ras oncogenes.

- The work presented here would be of broad interest for people in the cell biology field.

The expertise of this reviewer is in Ras-superfamily proteins, proteomics, cell migration/invasion and as such was qualified to assess this manuscript in its entirety.

Reviewer 3

Evidence, reproducibility and clarity

Summary:

The manuscript entitled "Proximity labelling identifies pro-migratory endocytic recycling cargo and machinery of the Rab4 and Rab11 families" by Wilson et al, presents an approach, BioID, able to identify and characterize protein complexes associated with Rab proteins in an ovarian cancer cell line. They started the study by coupling a proximity labelling method to mass spectrometry. By doing so they identified the interactomes associated with Rab4a, Rab11a and Rab25. Next, the authors proceeded to detect directly biotinylated peptides.

© 2023. Published by The Company of Biologists under the terms of the Creative Commons Attribution License (https://creativecommons.org/licenses/by/4.0/).

Then, using knock sideways experiments, the authors validated novel links between Rab11/Rab25 and some of the direct interactors identified. Lastly, they propose that SH3BP5L and CRACR2A are required for migration of ovarian cancer cells, in 3D-cell derived matrix.

Major comments:

1) A major limitation of the study is the reliance on a single migratory cell line, the A2780 cell line. As such the authors should include additional cell lines for their key experiments throughout the manuscript.

2) The authors state that BioID-Rabs are expressed at a "level close to endogenous". This should be quantified. Also, authors should clearly show that BioID-Rabs co-localize with the endogenous Rabs. So, immunofluorescence labelling of endogenous Rabs and markers for Early Endosomes (e.g. Rab5, EEA1, etc) and Recycling Endosomes should be performed.

3) In the dot-plot of the high-confidence proximal analysis, the average intensity (represented in the circle colour) should be normalized by the abundance of protein.

4) The knock sideways experiments validated high affinity prey interactions, including of sorting nexins with Rab4/11/25. SNX1 and SNX3 showed that they would only significantly redistribute in FKBP-GFP-Rab11a and FKBP-GFP-Rab25, respectively. Authors should comment on why the role of SNX1 and SNX3 was not assessed in migration studies.

5) Knock sideways showed that Rab4 was unable to induce significant re-localization of CLINT1. This would suggest that CLINT1 would be a candidate less robust than others identified by BioID and validated by knock sideways experiments. Why did the authors decide to proceed to assess the role of CLINT1 in migration studies?

6) Although the authors reported a lack of significant re-localization of CLINT1 by Rab4a, they state that "CLINT1 plays a role in Rab4 (but not Rab25) dependent migration in 3D- CDM". Can the authors comment on this?

7) "CLINT1 was identified as a Rab4, -11 and -25 proximal protein (Figure 2)". The study would benefit from additional evidence showing that CLINT1 does not act downstream of Rab11 to control migration of A2780 cells.

8) Authors should include immunofluorescence studies to better characterise the role of Rab4a, Rab11a and Rab25 networks in migration, adhesion and leading-edge related processes. Focal adhesions should be quantified, and actin cytoskeleton described. Such studies should be coupled to the cell migration studies. These would validate and support the conclusions drawn from the GO analysis.

9) In the discussion, the authors mention two other papers in which "proximity labelling methods have proven an excellent tool for identification of protein complexes, including for Rab4 and Rab11". The authors should also discuss if there are overlapping results.

Minor comments:

1) Figure 1: Panel A is too small. Insets are hard to interpret. The size of the whole panel should be increased.

2) Description of results regarding the trafficking machinery associated with Rab4a, Rab11a and Rab25 does not follow the same organization and structure as in Figure 2. The authors should try to match the organization of data and its description to improve readability.

3) In Figure 4B and S4C there are two labels for 1 and 2.

4) Figure S4E merge of GFP-FKBP Rab11a cells shows poor overlap. A replacement should be considered.

© 2023. Published by The Company of Biologists under the terms of the Creative Commons Attribution License (https://creativecommons.org/licenses/by/4.0/).

There are several typos in the discussion and in Figure 7 ("CRACRA" should be CRACR2A)

Significance

o The manuscript presents an approach that allow the identification of Rab-associated networks and the direct comparison between GTAses. This is of relevance since we still lack robust methodologies to identify the endosomal trafficking machinery underlying migration in cancer cells.

By not targeting Rab4 specific machinery (e.g. TBC1D5), the authors missed the opportunity to expand the knowledge regarding the machinery sustaining Rab4-dependent migration in cancer cells.

o The work targets an audience interested in endosomal trafficking and protein recycling in cancer cell migration.

The reviewer is a translational cancer biologist with expertise in cytoskeleton, endosomal recycling, signaling and cancer.

Review Commons Revision Plan

Manuscript number: RC-2022-01479
Corresponding author(s): Patrick Caswell

[The "revision plan" should delineate the revisions that authors intend to carry out in response to the points raised by the referees. It also provides the authors with the opportunity to explain their view of the paper and of the referee reports.

The document is important for the editors of affiliate journals when they make a first decision on the transferred manuscript. It will also be useful to readers of the reprint and help them to obtain a balanced view of the paper.

If you wish to submit a full revision, please use our "Full Revision" template. ***It is important to use the appropriate template to clearly inform the editors of your intentions.]***

1. General Statements [optional]

This section is optional. Insert here any general statements you wish to make about the goal of the study or about the reviews.

The reviewers highlight the power of our approach that allows "*the identification of Rab-associated networks and the direct comparison between GTPases*" and indicate that whilst our manuscript is "*very interesting and undoubtedly will be of a good use for many laboratories*" and "*the data appears of excellent quality and most of the conclusions or interpretations are correct*" it is more limited in terms of insight and mechanistic analysis. We therefore feel that the paper is most appropriately considered a 'tools and resources' style article.

2. Description of the planned revisions

Insert here a point-by-point reply that explains what revisions, additional experimentations and analyses are planned to address the points raised by the referees.

Reviewer 2
2nd major comment
The description of the BioID data is poorly structured and descriptive, a recurring challenge with big data paper. One suggestion to improve the manuscript would be to exploit the best-known interactions to clearly benchmark the efficiency of the screens. Next the new interactions could be

© 2023. Published by The Company of Biologists under the terms of the Creative Commons Attribution License (https://creativecommons.org/licenses/by/4.0/).

described and figure 2 could be better exploited in that respect (the mentioned complexes could be better drawn etc.). The text could also be more focused on fewer interactors such that it is more digestible for the readers. The major weakness of the manuscript, in my opinion, is the lack of depth in testing functionally some of the uncovered novel interactions.

The manuscript is currently structured to introduce the wider context of RabGTPase associated proteins through gene ontology, before focusing on classes of associated proteins with specific attention paid to trafficking machinery. We prefer to keep this style which we feel is appropriate for a 'tools and resources' style article, but the reviewer makes an excellent point around "benchmarking". We will combine the data in Figures 1 and 2 with exemplar protein complexes (including those known to bind RAB4/11/25 and potential new connections) depicted in more detail. We will then be able to restructure the text around these to make the detailed information more accessible to the reader.

3rd major comment

Some additional experiments that would be needed to support the claim of novelty in the paper include testing the function of some of the tested interactions. For example, the novel GEF interactions would benefit from biochemical testing in addition to BioID. Likewise, the section on biotinylation and interaction domain mapping is interesting but is, as presented, a theory. Using one interaction to dissect in more details to support this claim is needed. Alternatively, can the authors demonstrate that this approach can be used to confirmed known protein domains involved in protein-protein interactions of these Rabs? Finally, the authors end their manuscript by screening candidates issued from their BioID which have not been implicated in migration/invasion before. This is somewhat preliminary and fails to provide some depth into the function of one of these potential interactions (domain mapping, knockdown rescue of wt or mutants etc.).

We do show in the manuscript that integrins beta-1 and beta-5 are biotinylated in the cytoplasmic tail in the region we have identified as critical for the direct interaction between Rab25 and beta-1. We have unpublished biochemical data for this direct interaction first described in Caswell et al. 2007 (using beta-1 integrin truncations and point mutations to reveal the site of interaction) that will be included in a new figure to support this point. Similarly, we will use the known interactor Rab11-FIP5 and it's biotinylation pattern with respect to the known interaction sites to exemplify this. We will generate a new figure that shows the biotinylation pattern/interaction sites in structural representations of bait and prey. It may also be possible to model SH3BP5L and Rab25 interaction based on the published Rab11-SH3BP5L complex (Jenkins et al. Nat. Comms 2018).

4th major comment

The authors use the knock-sideways technique to validate the strength of their interaction. This is a clever way to validate interaction in cellulo which could be difficult using conventional IP. However, it looks like the expression of FRB-MITO leads to mitochondria fragmentation and aggregation. Is it possible that this cause a bias in their quantification analysis because it becomes difficult to clearly delineate individual mitochondria? In some cases (ex. Fig 5C), the recruitment of the candidate is obvious. However, in other cases (ex. Figure 5A) the recruitment to the mitochondria is not very convincing and looks more like the candidates collapse around the aggregated mitochondria. The authors should therefore describe the limitations in more details.
The reviewer is correct that relocalisation can cause mitochondrial aggregation (although we have not noticed fragmentation), and indeed Steve Royle's lab noted the same upon relocalisation of intracellular nanovesicle proteins of the TPD52 family (which interact with Rab4a, Rab11a and Rab25), even showing EM data that demonstrates vesicles docked with more than one mitochondrial surface (Larocque et al 2019, 2021). We see this aggregation with Rab4a, 11a and 25 to similar extents, and feel that in effect they can act as 'control' for one another in this regard since the three GTPases do not show the same re-localization of individual baits. We performed the analysis on deconvolved images which have the highest resolution, however 5A appears to be the native image. This is easily replaced and individual panels will be shown to give a clearer indication of mitochondrial localisation.

Reviewer 2 Minor comments:

-In Figure 1C, it is difficult to read the name on the candidates. The authors should fit the entire name in the nodes (maybe use an ellipse instead of a circle).

© 2023. Published by The Company of Biologists under the terms of the Creative Commons Attribution License (https://creativecommons.org/licenses/by/4.0/).

Thanks for this suggestion, we will resize the nodes/text.

-In Figure 1C and 2 the known interactors could be in a different color emphasize the new potential interactors.
Thanks for this suggestion, we will do this.

-Figure 4 is very heavy and the images are small making difficult to see the results clearly. Instead of showing 10 time points per condition, 3 or 4 time point with higher resolution images would have been more appropriate.
We will reduce the number of images as suggested.

Reviewer 3

Major comments:
1) A major limitation of the study is the reliance on a single migratory cell line, the A2780 cell line. As such the authors should include additional cell lines for their key experiments throughout the manuscript.

We will repeat cell migration experiments in other relevant cell lines that are commonly used and show high levels of cell migration/invasion (MDA-MB-231, H1299).

2) The authors state that BioID-Rabs are expressed at a "level close to endogenous". This should be quantified. Also, authors should clearly show that BioID-Rabs co-localize with the endogenous Rabs. So, immunofluorescence labelling of endogenous Rabs and markers for Early Endosomes (e.g. Rab5, EEA1, etc) and Recycling Endosomes should be performed.

We can quantify for Rab11. This is a good suggestion, we will perform immunfluorescence with markers of early endosomes/recycling endosomes (EEA1, transferrin, Rab11 (for Rab4/Rab25 expressing cells).

3) In the dot-plot of the high-confidence proximal analysis, the average intensity (represented in the circle colour) should be normalized by the abundance of protein.

This is indeed the case and we will make this more clear in legends/methods.

4) "CLINT1 was identified as a Rab4, -11 and -25 proximal protein (Figure 2)". The study would benefit from additional evidence showing that CLINT1 does not act downstream of Rab11 to control migration of A2780 cells.

We will make clear in the text that the level of direct biotinylation (shown in S3D) and the level of detected CLINT1 (shown in Figure 2) is far greater in Rab4a samples than Rab11a or Rab25. We are able to show that CLINT1 is not required for Rab25-driven migration (similar levels of CLINT1 are detected between Rab11 and Rab25)

5) Authors should include immunofluorescence studies to better characterise the role of Rab4a, Rab11a and Rab25 networks in migration, adhesion and leading-edge related processes. Focal adhesions should be quantified, and actin cytoskeleton described. Such studies should be coupled to the cell migration studies. These would validate and support the conclusions drawn from the GO analysis.

This is a great suggestion- for Rab4 there is published data to support the involvement of Rab4 in adheison formation (Roberts et al. Curr Biol. 2011; Gu et al JCB 2011), and we will use knockdown of Rab4/Rab11 and expression of Rab25 to analyze differences in adhesion formation and the actin cytoskeleton to support the gene ontology analysis.

6) In the discussion, the authors mention two other papers in which "proximity labelling methods have proven an excellent tool for identification of protein complexes, including for Rab4 and Rab11". The authors should also discuss if there are overlapping results.

© 2023. Published by The Company of Biologists under the terms of the Creative Commons Attribution License (https://creativecommons.org/licenses/by/4.0/).

We will expand on this.

Minor comments:

• Figure 1: Panel A is too small. Insets are hard to interpret. The size of the whole panel should be increased.

This will be fixed.

• Description of results regarding the trafficking machinery associated with Rab4a, Rab11a and Rab25 does not follow the same organization and structure as in Figure 2. The authors should try to match the organization of data and its description to improve readability.

This will be improved as discussed for reviewer 2.

• In Figure 4B and S4C there are two labels for 1 and 2.

This will be fixed.

• Figure S4E merge of GFP-FKBP Rab11a cells shows poor overlap. A replacement should be considered.

This will be fixed.

• There are several typos in the discussion and in Figure 7 ("CRACRA" should be CRACR2A)

This will be fixed and the manuscript checked carefully

• The manuscript presents an approach that allow the identification of Rab-associated networks and the direct comparison between GTAses. This is of relevance since we still lack robust methodologies to identify the endosomal trafficking machinery underlying migration in cancer cells. By not targeting Rab4 specific machinery (e.g. TBC1D5), the authors missed the opportunity to expand the knowledge regarding the machinery sustaining Rab4-dependent migration in cancer cells.

We will explain our choice of follow up candidates more clearly. TBC1D5 knockdown will be attempted but experiments may not be completed in time for a revised version.

3. Description of the revisions that have already been incorporated in the transferred manuscript

Please insert a point-by-point reply describing the revisions that were already carried out and included in the transferred manuscript. If no revisions have been carried out yet, please leave this section empty.

Reviewer 1:
Additional comments:
1) There are no blots shown (only boxes) for Figure S1C-D. Data in Figure S1 doe shown that BirA-Rab11a is expressed in similar levels as endogenous Rab11. However, no data supporting similar statement for Rab4 and Rab25 is shown.

We are unsure what is meant by the first comment- blots appear visible in our version and that which we downloaded. Hopefully these are OK in the latest version too.

We compared the level of BirA-Rab11a to Rab11 endogenous levels because of the availability of antibodies (we are not aware of reliable antibodies for Rab4). Also, Rab25 is not usually expressed in the cells used here. We then used BirA-Rab11 expression level to approximate a low level expression of BirA-Rab4a and BirA-Rab25, reasoning that similar expression levels allow for more meaningful comparison.

The text now reads:

© 2023. Published by The Company of Biologists under the terms of the Creative Commons Attribution License (https://creativecommons.org/licenses/by/4.0/).

'……we adopted a system to select for low level expression of BioID-Rab4a, BioID-Rab11a, and BioID-Rab25 (alongside a cytoplasmic BioID control) at a level close to endogenous Rab11 protein (Figure S1A-C).'

2) The presence of specific proteins in BioID does not mean that they either directly bind or regulate particular BirA-Rab. For example, authors state "DENND4C, related to Drosophila Rab11 GEF CRAG, was enriched to Rab11a, suggesting that this could be an alternate GEF for Rab11". There is no data supporting such a statement in this manuscript. Actually, DENND4C is better known GEF for Rab10. Rab10 is also known as Rab present in recycling endosomes, thus, could have easily be present in Rab11a-positive recycling endosomes. There are numerous similar statements in the manuscript that implies functional connections between Rabs and BioID "hits" without providing any other functional data.

We had intended that the use of the word 'suggest' would temper any direct statement and apologise that this was not clear enough. It is however important to note that Francis Barr's lab (Yoshimura et al. 2020) compared DENND4B activity in a group of RabGTPases including Rab10 and Rab11a, with highest activity was noted towards Rab10 (although some activity toward Rab11a was noted), but DENND4C activity was only compared between Rab8, Rab10 and Rab13. Xiong et al. (2012) later showed CRAG (the DENND4 homologue in Drosophila) to have GEF activity towards Rab11 which is enhanced in the presence of calmodulin/Ca2+, and whilst Crag does have activity towards Rab10, it's role in Drosphila eye is through Rab11 (where Rab10 expression is low). Upon reflection we have therefore changed the text as below (page 6). We are also careful with our interpretations and have endeavoured to use 'could suggest' or similar wording to indicate that these are not direct conclusions from our data.

DENND4C, related to Drosophila Rab11 GEF CRAG and reported to have high activity towards Rab10 in mammalian cells (Xiong et al., 2012; Yoshimura et al., 2010), was enriched to Rab11a (longlist only, Supplementary Table 2), Rab11 could therefore potentially act upstream of Rab10 in a RabGTPase cascade, or (given that Rab10 was not identified in our dataset and analysis of DENND4C activity towards Rab11 has not been published) it is possible that DENND4C could act as an alternate GEF for Rab11.

We also include the paragraph below as an example of the caveats but also the connections our data could point towards (page 6):

Protein complex formation between RabGTPases and GEFs/GAPs is not necessarily indicative of a substrate: enzyme association, and in fact Rab and Arf cascades have been reported to operate via GTPase-GEF/GAP associations (D'Souza et al., 2014; Knödler et al., 2010; Ortiz et al., 2002). Exemplifying this, in addition to Rab regulators GEFs/GAPs for other Ras superfamily members were also identified (Figure 2; Supplementary Table 1 and 2), predominantly for Arf and Rho families,

3) Authors should not use RCP term to refer to Rab11FIP1 since Rab11FIP1 is its established name and using other names only creates confusion. RCP term was first used to indicate that Rab11FIP1 can bind to both Rab4 and Rab11, the hypothesis that since then was proven to be incorrect.

RCP changed to established name Rab11FIP1 throughout.

Reviewer 2:

1st major comment

For the most part, the data appears of excellent quality and most of the conclusions or interpretations are correct (see below for a few points that can be improved). Some key concepts are missing in the introduction. For example, the concept of GEFs and GAPs only appears later in the experimental section - this should be introduced earlier.

We thank the reviewer for pointing this out and have made this correction on page 1 as follows:

"RabGTPases interact with Rab guanine dissociation inhibit (GDIs) or Rab-escorpt protein (REP) in the cytoplasm, REP facilitates geranylgeranylation at the C-terminus to allow interactions with membranes. Like other GTPases, Rabs cycle through active GTP-bound and inactive GDP-bound

© 2023. Published by The Company of Biologists under the terms of the Creative Commons Attribution License (https://creativecommons.org/licenses/by/4.0/).

states regulated by guanine nucleotide exchange factors (GEFs) and GTPase activating proteins (GAPs) respectively (Stenmark, 2009).”

Minor comments:

-The authors aim to identify new interactors involved in migration, but they performed the BioID on confluent cells where cell migration is likely limited. Would comparing a BioID performed on confluent cells with one where the cells are sparse enough to migrate possibly interesting to conduct? This could be discussed.

We do state in the methods that cells were plated such that they are near-confluent when the BioID experiments are performed, and cells therefore still have space to move into. We make this point more clear in the text on page 18:

“Cells expressing BioID fusion protein constructs were plated onto tissue culture plates as at a density to ensure near-confluency and space for cell motility the following day.”

Methods :The methods are well described. It is a bit surprising that the BioID samples are run on SDS-PAGE and that bands are cut when on beads digestion is currently done by many lab for this technique.

On-bead digestion can be problematic, we optimized our methodology such that streptavidin bound proteins could be eluted to minimize the amount of streptavidin in the sample itself. We therefore preferred in gel digestion for these experiments to prevent streptavidin peptides dominating the peptides identified by LC-MS/MS. We have made this more clear in the mehtods:

“The entire complement of proteins in the ‘gel-top’ protein band were excised and subjected to in-gel digestion as part of an optimised method to prevent contamination of samples with streptavidin peptides (which are released by harsh elution/on-bead digestion in our hands).”

The authors looked at endogenous Rab11 vs BioID-Rab11. Why no do it for the other 2 Rabs. Also, quantification of endo/exo expression should be done.

As indicated for reviewer 1: We compared the level of BirA-Rab11a to Rab11 endogenous levels because of the availability of antibodies (we are not aware of reliable antibodies for Rab4). Also, Rab25 is not usually expressed in the cells used here. We then used BirA-Rab11 expression level to approximate a low level expression of BirA-Rab4a and BirA-Rab25, reasoning that similar expression levels allow for more meaningful comparison.

The text now reads:

‘……we adopted a system to select for low level expression of BioID-Rab4a, BioID-Rab11a, and BioID-Rab25 (alongside a cytoplasmic BioID control) at a level close to endogenous Rab11 protein (Figure S1A-C).’

3. Description of analyses that authors prefer not to carry out

Please include a point-by-point response explaining why some of the requested data or additional analyses might not be necessary or cannot be provided within the scope of a revision. This can be due to time or resource limitations or in case of disagreement about the necessity of such additional data given the scope of the study. Please leave empty if not applicable.

Reviewer 2 (minor comment)

Statistics: Statistics should be provided for all quantification, not only the one that are significant. For the non-significant, the P-value should be indicated on the figure.

We don’t agree with this point- we indicate where all statistical differences are between data in graphs using symbols for clarity, but p values on their own are rather meaningless (and leave figures looking over cluttered).

© 2023. Published by The Company of Biologists under the terms of the Creative Commons Attribution License (https://creativecommons.org/licenses/by/4.0/).

Reviewer 3

4) The knock sideways experiments validated high affinity prey interactions, including of sorting nexins with Rab4/11/25. SNX1 and SNX3 showed that they would only significantly redistribute in FKBP-GFP-Rab11a and FKBP-GFP-Rab25, respectively. Authors should comment on why the role of SNX1 and SNX3 was not assessed in migration studies.

We would have liked to perform these experiments but lacked time. It may be possible to do so, if not we will explain prioritization of other candidates.

5) Knock sideways showed that Rab4 was unable to induce significant re-localization of CLINT1. This would suggest that CLINT1 would be a candidate less robust than others identified by BioID and validated by knock sideways experiments. Why did the authors decide to proceed to assess the role of CLINT1 in migration studies?

Knock sideways is likely to indicate the affinity of interaction, but we felt that the strong indication of close interaction given by biotinylation data, plus the fact that co-localization was evident, gave us sufficient reason to follow up CLINT1.

6) Although the authors reported a lack of significant re-localization of CLINT1 by Rab4a, they state that "CLINT1 plays a role in Rab4 (but not Rab25) dependent migration in 3D-CDM". Can the authors comment on this?

We feel the data in Figure S6A demonstrate that knockdown of CLINT1 slows the migration of cells that lack Rab25 expression, but have no effect on the speed of migration when Rab25 is expressed. This together with co-localisation and biotinylation data suggest a functional relationship between Rab4a and CLINT1.

Original submission

First decision letter

MS ID#: JOCES/2022/260468

MS TITLE: Proximity labelling identifies pro-migratory endocytic recycling cargo and machinery of the Rab4 and Rab11 families

AUTHORS: Beverley Wilson, Jakub Gemperle, Craig Lawless, Chloe Flett, Matthew Hartshorn, Eleanor Hinde, Thomas Harrison, Megan Chastney, Sarah Taylor, Jennifer Allen, and Patrick Caswell
ARTICLE TYPE: Tools and Resources

We have now reached a decision on the above manuscript.

To see a copy of this decision letter, please go to: https://submit-jcs.biologists.org and click on the 'Manuscripts with Decisions' queue in the Author Area. (Corresponding author only has access to reviews.)

Upon analysis of the Review Common Review, I generally agree with the revision plan and the fact that such a revised manuscript would be suitable for publication in the Journal of Cell Science. However, my concern is that the list of proposed experiments is long and demanding and may require a longer timeframe than that usually allocated with a revision. In this light prioritization of the planned experiments seems therefore necessary. Could the authors assemble such a list before embarking on further revisions of their paper? I will then be able to comment and agree on a resubmission timeframe.

I look forward to receiving this list and your revised manuscript in due course.

© 2023. Published by The Company of Biologists under the terms of the Creative Commons Attribution License (https://creativecommons.org/licenses/by/4.0/).

Best wishes,
Giampietro Schiavo
Editor
Journal of Cell Science

Journal of Cell Science
The Company of Biologists Ltd
Bidder Building
Station Road
Histon, Cambridge, CB24 9LF
Phone:44 (0)1223 632867
Fax:44 (0)1223 632869
jcs@biologists.com

The information contained in this message and any attachment is confidential, legally privileged and is intended for the addressee only. Any dissemination, distribution, copying, disclosure or use of this message/attachment or its contents is strictly prohibited and may be unlawful. No contract is intended or implied, unless confirmed by hard copy. If you have received this message in error, please inform the sender and delete it from your mailbox or any other storage mechanism.

The Company Of Biologists Ltd cannot accept liability for any statements made which are clearly the senders' own and not expressly made on behalf of the Company of Biologists Limited or one of their agents.

We thank the editor for their consideration of our manuscript, and we provide a prioritised list of revisions below. We have divided this into revisions requiring experiments, bioinformatics, or text/figure-based revisions. For the two high priority experimental revisions we have previously shown that Rab11 promotes formation of filopodia, and have assays set up that will allow us to quantify any differences in focal adhesion formation. We think these experiments will be completed within two months. For the second high priority experimental revision we have reagents in place and can complete this in one month. The lower priority experimental revisions involve use of other cells or knockdown of a target we do not have oligos/antibodies against. We have deemed experiments with other cell types as lower priority because they are complicated by the fact that we will first need to establish the requirement for Rab4, Rab11 and Rab25 in motility, but we will test the requirement for CRACR2A in invasive migration of cells which we know require Rab11-dependent trafficking for efficient migration/invasion (MDA-MB-231, H1299).

Revisions requiring experiments:

High priority 1
Reviewer 3 Major comment 8) Authors should include immunofluorescence studies to better characterise the role of Rab4a, Rab11a and Rab25 networks in migration, adhesion and leading-edge related processes. Focal adhesions should be quantified, and actin cytoskeleton described. Such studies should be coupled to the cell migration studies. These would validate and support the conclusions drawn from the GO analysis.

This is a great suggestion- for Rab4 there is published data to support the involvement of Rab4 in adhesion formation (Roberts et al. Curr Biol. 2011; Gu et al JCB 2011), and we will use knockdown of Rab4/Rab11 and expression of Rab25 to analyze differences in adhesion formation and the actin cytoskeleton to support the gene ontology analysis.

© 2023. Published by The Company of Biologists under the terms of the Creative Commons Attribution License (https://creativecommons.org/licenses/by/4.0/).

High priority 2
Reviewer 3 Major comment 2) The authors state that BioID-Rabs are expressed at a "level close to endogenous". This should be quantified. Also, authors should clearly show that BioID-Rabs co-localize with the endogenous Rabs. So, immunofluorescence labelling of endogenous Rabs and markers for Early Endosomes (e.g. Rab5, EEA1, etc.) and Recycling Endosomes should be performed.

We can quantify for Rab11. This is a good suggestion, we will perform immunofluorescence with markers of early endosomes/recycling endosomes (EEA1, transferrin, Rab11 (for Rab4/Rab25 expressing cells).

Lower priority 1
Reviewer 3 Major comment 1) A major limitation of the study is the reliance on a single migratory cell line, the A2780 cell line. As such the authors should include additional cell lines for their key experiments throughout the manuscript.

We will repeat cell migration experiments in other relevant cell lines that are commonly used and show high levels of cell migration/invasion (MDA-MB-231, H1299).

Lower priority 2

Reviewer 3 minor comment
By not targeting Rab4 specific machinery (e.g. TBC1D5), the authors missed the opportunity to expand the knowledge regarding the machinery sustaining Rab4-dependent migration in cancer cells.

We will explain our choice of follow up candidates more clearly. TBC1D5 knockdown will be attempted but experiments may not be completed in time for a revised version.

Revisions requiring bioinformatics (and use of existing data):

Reviewer 2 3rd major comment

Some additional experiments that would be needed to support the claim of novelty in the paper include testing the function of some of the tested interactions. For example, the novel GEF interactions would benefit from biochemical testing in addition to BioID. Likewise, the section on biotinylation and interaction domain mapping is interesting but is, as presented, a theory. Using one interaction to dissect in more details to support this claim is needed. Alternatively, can the authors demonstrate that this approach can be used to confirmed known protein domains involved in protein-protein interactions of these Rabs? Finally, the authors end their manuscript by screening candidates issued from their BioID which have not been implicated in migration/invasion before. This is somewhat preliminary and fails to provide some depth into the function of one of these potential interactions (domain mapping, knockdown rescue of wt or mutants etc.).

We do show in the manuscript that integrins beta-1 and beta-5 are biotinylated in the cytoplasmic tail in the region we have identified as critical for the direct interaction between Rab25 and beta-1. We have unpublished biochemical data for this direct interaction first described in Caswell et al. 2007 (using beta-1 integrin truncations and point mutations to reveal the site of interaction) that will be included in a new figure to support this point. Similarly, we will use the known interactor Rab11-FIP5 and it’s biotinylation pattern with respect to the known interaction sites to exemplify this. We will generate a new figure that shows the biotinylation pattern/interaction sites in structural representations of bait and prey. It may also be possible to model SH3BP5L and Rab25 interaction based on the published Rab11-SH3BP5L complex (Jenkins et al. Nat. Comms 2018). This analysis will be performed in collaboration with Thomas Zacharchenko, a structural biologist.

Text/figure revisions:

Reviewer 2 2nd major comment

© 2023. Published by The Company of Biologists under the terms of the Creative Commons Attribution License (https://creativecommons.org/licenses/by/4.0/).

The description of the BioID data is poorly structured and descriptive, a recurring challenge with big data paper. One suggestion to improve the manuscript would be to exploit the best-known interactions to clearly benchmark the efficiency of the screens. Next the new interactions could be described and figure 2 could be better exploited in that respect (the mentioned complexes could be better drawn etc.). The text could also be more focused on fewer interactors such that it is more digestible for the readers. The major weakness of the manuscript, in my opinion, is the lack of depth in testing functionally some of the uncovered novel interactions.

The manuscript is currently structured to introduce the wider context of RabGTPase associated proteins through gene ontology, before focusing on classes of associated proteins with specific attention paid to trafficking machinery. We prefer to keep this style which we feel is appropriate for a 'tools and resources' style article, but the reviewer makes an excellent point around "benchmarking". We will combine the data in Figures 1 and 2 with exemplar protein complexes (including those known to bind RAB4/11/25 and potential new connections) depicted in more detail. We will then be able to restructure the text around these to make the detailed information more accessible to the reader.

Reviewer 2 4th major comment

The authors use the knock-sideways technique to validate the strength of their interaction. This is a clever way to validate interaction in cellulo which could be difficult using conventional IP. However, it looks like the expression of FRB-MITO leads to mitochondria fragmentation and aggregation. Is it possible that this cause a bias in their quantification analysis because it becomes difficult to clearly delineate individual mitochondria? In some cases (ex. Fig 5C), the recruitment of the candidate is obvious. However, in other cases (ex. Figure 5A) the recruitment to the mitochondria is not very convincing and looks more like the candidates collapse around the aggregated mitochondria. The authors should therefore describe the limitations in more details.

The reviewer is correct that relocalisation can cause mitochondrial aggregation (although we have not noticed fragmentation), and indeed Steve Royle's lab noted the same upon relocalisation of intracellular nanovesicle proteins of the TPD52 family (which interact with Rab4a, Rab11a and Rab25), even showing EM data that demonstrates vesicles docked with more than one mitochondrial surface (Larocque et al 2019, 2021). We see this aggregation with Rab4a, 11a and 25 to similar extents, and feel that in effect they can act as 'control' for one another in this regard since the three GTPases do not show the same re-localization of individual baits. We performed the analysis on deconvolved images which have the highest resolution, however 5A appears to be the native image. This is easily replaced and individual panels will be shown to give a clearer indication of mitochondrial localisation.

Reviewer 2 Minor comments:

-In Figure 1C, it is difficult to read the name on the candidates. The authors should fit the entire name in the nodes (maybe use an ellipse instead of a circle).

Thanks for this suggestion, we will resize the nodes/text.

-In Figure 1C and 2 the known interactors could be in a different color emphasize the new potential interactors.

Thanks for this suggestion, we will do this.

-Figure 4 is very heavy and the images are small making difficult to see the results clearly. Instead of showing 10 time points per condition, 3 or 4 time point with higher resolution images would have been more appropriate.

We will reduce the number of images as suggested.

Reviewer 3

3) In the dot-plot of the high-confidence proximal analysis, the average intensity (represented in the circle colour) should be normalized by the abundance of protein.

© 2023. Published by The Company of Biologists under the terms of the Creative Commons Attribution License (https://creativecommons.org/licenses/by/4.0/).

This is indeed the case and we will make this more clear in legends/methods.

7) "CLINT1 was identified as a Rab4, -11 and -25 proximal protein (Figure 2)". The study would benefit from additional evidence showing that CLINT1 does not act downstream of Rab11 to control migration of A2780 cells.

We will make clear in the text that the level of direct biotinylation (shown in S3D) and the level of detected CLINT1 (shown in Figure 2) is far greater in Rab4a samples than Rab11a or Rab25. We are able to show that CLINT1 is not required for Rab25-driven migration (similar levels of CLINT1 are detected between Rab11 and Rab25)

9) In the discussion, the authors mention two other papers in which "proximity labelling methods have proven an excellent tool for identification of protein complexes, including for Rab4 and Rab11". The authors should also discuss if there are overlapping results.

We will expand on this.

Minor comments:
• Figure 1: Panel A is too small. Insets are hard to interpret. The size of the whole panel should be increased.

This will be fixed.

• Description of results regarding the trafficking machinery associated with Rab4a, Rab11a and Rab25 does not follow the same organization and structure as in Figure 2. The authors should try to match the organization of data and its description to improve readability.

This will be improved as discussed for reviewer 2.

• In Figure 4B and S4C there are two labels for 1 and 2.

This will be fixed.

• Figure S4E merge of GFP-FKBP Rab11a cells shows poor overlap. A replacement should be considered.

This will be fixed.

• There are several typos in the discussion and in Figure 7 ("CRACRA" should be CRACR2A)

This will be fixed and the manuscript checked carefully

<u>Author response to reviewers' comments</u>

We thank the editor for their consideration of our manuscript, and the reviewers for their insightful comments that have improved our manuscript. The reviewers highlight the power of our approach that allows “the identification of Rab-associated networks and the direct comparison between GTPases” and indicate that whilst our manuscript is “very interesting and undoubtedly will be of a good use for many laboratories” and “the data appears of excellent quality and most of the conclusions or interpretations are correct” it is more limited in terms of insight and mechanistic analysis. We therefore feel that the paper is most appropriately considered a ‘tools and resources’ style article. Here we provide a point-by-point response (in red) to the reviewer’s comments (in black). In addition, as suggested by the editor, we performed migration experiments in additional cell lines as indicated in Figures 7 and 8.

Reviewer #1 (Evidence, reproducibility and clarity (Required)):
From methodology and reproducibility point of view it is an excellent manuscript. It is also well-written.

© 2023. Published by The Company of Biologists under the terms of the Creative Commons Attribution License (https://creativecommons.org/licenses/by/4.0/).

We thank the reviewer for their positive assessment and suggestions.

Reviewer #1 (Significance (Required)):
This is a manuscript that presents an in-depth analysis of potential interactome and cross-interactome of Rab11a, Rab4a and Rab25 GTPases. BioID and knock-sideway data presented in first half of the manuscript is very interesting and undoubtedly will be of a good use for many laboratories. However, authors tend to over-interpret some of their data, suggesting functional connections between specific Rabs and BioID hits without any additional data. Authors admit themselves that there are some disconnects between BioID and knock-sideways data. Furthermore, BioID measure proximity and not functional connection.

Second half of the manuscript focuses on taking some of the BioID hits and testing whether they are required for mediating cell migration. By itself, it is a great idea since that would provide that functional connection that is missing in the original BioID screen. Unfortunately, the data is limited to few knock-downs without any further analyses of the involvement of these proteins in regulating migration. Consequently, as it stands, this manuscript is essentially a BioID screen with limited insights or validation of specific "hits", thus, does not really lead to any new conclusions about cross-function of Rab11, Rab25 and Rab4 networks.

We feel this manuscript is best considered as a 'tools and resources' style article.

Reviewer 1 Additional comments:
1) There are no blots shown (only boxes) for Figure S1C-D. Data in Figure S1 doe shown that BirA-Rab11a is expressed in similar levels as endogenous Rab11. However, no data supporting similar statement for Rab4 and Rab25 is shown.

We are unsure what is meant by the first comment- blots appear visible in our version and that which we downloaded. Hopefully these are OK in the latest version too.
We compared the level of BirA-Rab11a to Rab11 endogenous levels because of the availability of antibodies (we are not aware of reliable antibodies for Rab4). Also, Rab25 is not usually expressed in the cells used here. We then used BirA-Rab11 expression level to approximate a low-level expression of BirA-Rab4a and BirA-Rab25, reasoning that similar expression levels allow for more meaningful comparison.

As noted for Reviewer 2 and 3, we performed new experiments where we allow uptake of transferrin for 10 minutes (to label early endosomes) or 30 minutes (to label recycling endosomes). In these experiments we see good overlap between internalised transferrin and BioID-Rab4 at 10 minutes, and with BioID-Rab11a and -Rab25 at 30 minutes, indicating that the modified Rabs localize to the appropriate compartments despite the modest overexpression. We also include quantification of Rab11 endo/exo expression levels in Figure 1 for reviewers at the end of this document.

2) The presence of specific proteins in BioID does not mean that they either directly bind or regulate particular BirA-Rab. For example, authors state "DENND4C, related to Drosophila Rab11 GEF CRAG, was enriched to Rab11a, suggesting that this could be an alternate GEF for Rab11". There is no data supporting such a statement in this manuscript. Actually, DENND4C is better known GEF for Rab10. Rab10 is also known as Rab present in recycling endosomes, thus, could have easily be present in Rab11a-positive recycling endosomes. There are numerous similar statements in the manuscript that implies functional connections between Rabs and BioID "hits" without providing any other functional data.

We had intended that the use of the word 'suggest' would temper any direct statement and apologise that this was not clear enough. It is however important to note that Francis Barr's lab (Yoshimura et al. 2020) compared DENND4B activity in a group of Rab GTPases including Rab10 and Rab11a, with highest activity was noted towards Rab10 (although some activity toward Rab11a was noted), but DENND4C activity was only compared between Rab8, Rab10 and Rab13. Xiong et al. (2012) later showed CRAG (the DENND4 homologue in Drosophila) to have GEF activity towards Rab11 which is enhanced in the presence of calmodulin/Ca2+, and whilst Crag does have activity towards Rab10, it's role in Drosophila eye is through Rab11 (where Rab10 expression is low). Upon

© 2023. Published by The Company of Biologists under the terms of the Creative Commons Attribution License (https://creativecommons.org/licenses/by/4.0/).

reflection we have therefore changed the text as below (page 5). We are also careful with our interpretations and have endeavoured to use ‘could suggest’ or similar wording to indicate that these are not direct conclusions from our data.
“DENND4C, related to Drosophila Rab11 GEF CRAG and reported to have high activity towards Rab10 in mammalian cells (Xiong et al., 2012; Yoshimura et al., 2010), was enriched to Rab11a (longlist only, Supplementary Table 2). Rab11 could therefore potentially act upstream of Rab10 in a Rab GTPase cascade, or (given that Rab10 was not identified in our dataset and analysis of DENND4C activity towards Rab11 has not been published) it is possible that DENND4C could act as an alternate GEF for Rab11.”

We also include the paragraph below as an example of the caveats but also the connections our data could point towards (page 6):

“Protein complex formation between Rab GTPases and GEFs/GAPs is not necessarily indicative of a substrate: enzyme association, and in fact Rab and Arf cascades have been reported to operate via GTPase-GEF/GAP associations (D’Souza et al., 2014; Knödler et al., 2010; Ortiz et al., 2002). Exemplifying this, in addition to Rab regulators GEFs/GAPs for other Ras superfamily members were also identified (Figure 3; Supplementary Table 1 and 2). These were predominantly regulators of Arf and Rho families,….”

3) Authors should not use RCP term to refer to Rab11FIP1 since Rab11FIP1 is its established name and using other names only creates confusion. RCP term was first used to indicate that Rab11FIP1 can bind to both Rab4 and Rab11, the hypothesis that since then was proven to be incorrect.

RCP changed to established name Rab11FIP1 throughout.

Reviewer #2 (Evidence, reproducibility and clarity (Required)):
Wilson et al. submitted a paper entitled: "Proximity labelling identifies pro-migratory endocytic recycling cargo and machinery of the Rab4 and Rab11 families". The goal of the paper is to identify new interactors for RAB4/11 and 25 that could be involved in Rab-dependent migration. To do so, they used BioID of the aforementioned 3 Rabs in mesenchymal and migratory ovarian cancer cell line A2780. They validate some of the interactors using the knock-sideways technique and test the requirement of some interactor for migration/invasion in a 3D matrix. This is a very descriptive paper that could benefit from more in-depth mechanistic analysis of fewer candidates.

We feel this manuscript is best considered as a ‘tools and resources’ style article.

Major comments:
For the most part, the data appears of excellent quality and most of the conclusions or interpretations are correct (see below for a few points that can be improved). Some key concepts are missing in the introduction. For example, the concept of GEFs and GAPs only appears later in the experimental section - this should be introduced earlier.

We thank the reviewer for their positive comments and for pointing this out- have made this correction on page 1 as follows:

“Rab GTPases interact with Rab guanine dissociation inhibit (GDIs) or Rab-escort protein (REP) in the cytoplasm, REP facilitates geranylgeranylation at the C-terminus to allow interactions with membranes. Like other GTPases, Rabs cycle through active GTP-bound and inactive GDP-bound states regulated by guanine nucleotide exchange factors (GEFs) and GTPase activating proteins (GAPs) respectively (Stenmark, 2009).”

The description of the BioID data is poorly structured and descriptive, a recurring challenge with big data paper. One suggestion to improve the manuscript would be to exploit the best-known interactions to clearly benchmark the efficiency of the screens. Next the new interactions could be described and figure 2 could be better exploited in that respect (the mentioned complexes could be better drawn etc.). The text could also be more focused on fewer interactors such that it is more digestible for the readers. The major weakness of the manuscript, in my opinion, is the lack of depth in testing functionally some of the uncovered novel interactions.

© 2023. Published by The Company of Biologists under the terms of the Creative Commons Attribution License (https://creativecommons.org/licenses/by/4.0/).

The manuscript is structured as a ‘tools and resources’ style article, and we have emphasised the links between our data and known interactors, as well as highlighting potential new associations with trafficking machinery etc. that are of interest to those in the field. We introduce the wider context of Rab GTPase associated proteins through gene ontology, before focusing on classes of associated proteins with specific attention paid to trafficking machinery. We prefer to keep this style which we feel is appropriate for the type of article.

Some additional experiments that would be needed to support the claim of novelty in the paper include testing the function of some of the tested interactions. For example, the novel GEF interactions would benefit from biochemical testing in addition to BioID. Likewise, the section on biotinylation and interaction domain mapping is interesting but is, as presented, a theory. Using one interaction to dissect in more details to support this claim is needed. Alternatively, can the authors demonstrate that this approach can be used to confirmed known protein domains involved in protein-protein interactions of these Rabs? Finally, the authors end their manuscript by screening candidates issued from their BioID which have not been implicated in migration/invasion before. This is somewhat preliminary and fails to provide some depth into the function of one of these potential interactions (domain mapping, knockdown rescue of wt or mutants etc.).

We discuss the known interactor Rab11-FIP5 and its biotinylation pattern with respect to the known interaction sites to exemplify this. We show in the manuscript that integrin beta-1 is biotinylated within the cytoplasmic tail in the same region we now show using new biochemical data is critical for the direct interaction between Rab25 and beta-1 (new Figure S4A). We modelled the SH3BP5:Rab11a complex (Jenkins et al. Nat. Comms 2018) and used homology to SH3BP5L to indicate how the Rab25 binding site is potentially different in SH3BP5L. Biochemical testing of GEF activity will form part of our future plans, but we fell this is beyond the scope of a tools and resources style article.

The authors use the knock-sideways technique to validate the strength of their interaction. This is a clever way to validate interaction in cellulo which could be difficult using conventional IP. However, it looks like the expression of FRB-MITO leads to mitochondria fragmentation and aggregation. Is it possible that this cause a bias in their quantification analysis because it becomes difficult to clearly delineate individual mitochondria? In some cases (ex. Fig 5C), the recruitment of the candidate is obvious. However, in other cases (ex. Figure 5A) the recruitment to the mitochondria is not very convincing and looks more like the candidates collapse around the aggregated mitochondria. The authors should therefore describe the limitations in more details.

The reviewer is correct that relocalisation can cause mitochondrial aggregation (although we have not noticed fragmentation), and indeed Steve Royle’s lab noted the same upon relocalisation of intracellular nanovesicle proteins of the TPD52 family (which interact with Rab4a, Rab11a and Rab25), even showing EM data that demonstrates vesicles docked with more than one mitochondrial surface (Larocque et al 2019, 2021). We see this aggregation with Rab4a, 11a and 25 to similar extents, and feel that in effect they can act as ‘control’ for one another in this regard since the three GTPases do not show the same re-localization of individual baits. We performed the analysis on deconvolved images which have the highest resolution, however we have replaced some images including 5A to give a clearer indication of mitochondrial localisation.

Minor comments:
-The authors aim to identify new interactors involved in migration, but they performed the BioID on confluent cells where cell migration is likely limited. Would comparing a BioID performed on confluent cells with one where the cells are sparse enough to migrate possibly interesting to conduct? This could be discussed.

We state in the methods that cells were plated such that they are near-confluent when the BioID experiments are performed, and cells therefore still have space to move into. We make this point more clear in the text on page 13:

“Cells expressing BioID fusion protein constructs were plated onto tissue culture plates at a density to ensure near-confluency and space for cell motility the following day.”

© 2023. Published by The Company of Biologists under the terms of the Creative Commons Attribution License (https://creativecommons.org/licenses/by/4.0/).

-In Figure 1C, it is difficult to read the name on the candidates. The authors should fit the entire name in the nodes (maybe use an ellipse instead of a circle).

Thanks for this suggestion, we have resized the nodes/text.

-In Figure 1C and 2 the known interactors could be in a different color emphasize the new potential interactors.

Thanks for this suggestion, but we prefer to leave the figure as it is.

-Figure 4 is very heavy and the images are small making difficult to see the results clearly. Instead of showing 10 time points per condition, 3 or 4 time point with higher resolution images would have been more appropriate.

Thanks for this suggestion, but we prefer to show the full dynamics of relocalisation (given they are different for Rab11/25 and 'prey' Rab11-FIP5).

Methods :The methods are well described. It is a bit surprising that the BioID samples are run on SDS-PAGE and that bands are cut when on beads digestion is currently done by many lab for this technique.

On-bead digestion proved problematic for us, so we optimized our methodology such that streptavidin bound proteins could be eluted to minimize the amount of streptavidin in the sample itself. We therefore preferred in gel digestion for these experiments to prevent streptavidin peptides dominating the peptides identified by LC-MS/MS. We have made this clearer in the methods:

"The entire complement of proteins in the 'gel-top' protein band were excised and subjected to in-gel digestion as part of an optimised method to prevent contamination of samples with streptavidin peptides (which are released by harsh elution in our hands)."

Statistics: Statistics should be provided for all quantification, not only the one that are significant. For the non-significant, the P-value should be indicated on the figure.

We respectfully do not agree with this point- we indicate where all statistical differences are between data in graphs using symbols for clarity, but p values on their own are rather meaningless (and leave figures looking over cluttered).

The authors looked at endogenous Rab11 vs BioID-Rab11. Why no do it for the other 2 Rabs. Also, quantification of endo/exo expression should be done.

As indicated for reviewer 1 and 3 : We compared the level of BirA-Rab11a to Rab11 endogenous levels because of the availability of antibodies (we are not aware of reliable antibodies for Rab4). Also, Rab25 is not usually expressed in the cells used here. We used BirA-Rab11 expression level to approximate a low level expression of BirA-Rab4a and BirA-Rab25, reasoning that similar expression levels allow for more meaningful comparison.

The text now reads:
'……we adopted a system to select for low level expression of BioID-Rab4a, BioID-Rab11a, and BioID-Rab25 (alongside a cytoplasmic BioID control) at a level close to endogenous Rab11 protein (Figure S1A-C).'

We also include quantification of Rab11 endo/exo expression levels in Figure 1 for reviewers at the end of this document.

Reviewer #2 (Significance (Required)):
The advance of this work is to expand the potential functional interactome of three Rabs involved in slow recycling of endosomes. Some novel interactions are reported and some screening approaches have been use to reveal functional ones (this could be improved).

© 2023. Published by The Company of Biologists under the terms of the Creative Commons Attribution License (https://creativecommons.org/licenses/by/4.0/).

This work is potentially important and part of the priorities in the field to ascribe the overlapping and specific interactions/functions of Rab subfamilies. Similar work has been done for Rho proteins and selected Ras oncogenes.

The work presented here would be of broad interest for people in the cell biology field.
The expertise of this reviewer is in Ras-superfamily proteins, proteomics, cell migration/invasion and as such was qualified to assess this manuscript in its entirety.

We thank the reviewer for their fair assessment of our work.

Reviewer #3 (Evidence, reproducibility and clarity (Required)):
Summary:
The manuscript entitled "Proximity labelling identifies pro-migratory endocytic recycling cargo and machinery of the Rab4 and Rab11 families" by Wilson et al, presents an approach, BioID, able to identify and characterize protein complexes associated with Rab proteins in an ovarian cancer cell line. They started the study by coupling a proximity labelling method to mass spectrometry. By doing so they identified the interactomes associated with Rab4a, Rab11a and Rab25. Next, the authors proceeded to detect directly biotinylated peptides. Then, using knock sideways experiments, the authors validated novel links between Rab11/Rab25 and some of the direct interactors identified. Lastly, they propose that SH3BP5L and CRACR2A are required for migration of ovarian cancer cells, in 3D-cell derived matrix.

Major comments:
1) A major limitation of the study is the reliance on a single migratory cell line, the A2780 cell line. As such the authors should include additional cell lines for their key experiments throughout the manuscript.

We thank the reviewer for these comments that helped us strengthen our manuscript. We chose to use BT20 cells, a triple negative breast cancer cell line, for experiments in a new Figure 7/S7 as this line also expresses Rab25 (described in the text on page 8):

“We next sought to confirm our findings using BT20 triple negative breast cancer cells, which express Rab4a/b, 11a/b and 25 (Figure S7D). BT20 cells maintain directional persistence as they migrate in 3D-CDM, but depletion of Rab4a/b, Rab11a/b or Rab25 decreased both speed and persistence of motility (Figure 7E, F, S7C, D), indicating that migration of this cell line requires each of the Rab GTPase subfamilies. Depletion of the Rab4 recruited trafficking machinery CLINT1, or the Rab25 GEF SH3BP5L, reduced the speed of migration of BT20 cells in 3D-CDM (Figure 7G, H, S7E), suggesting that their function in motility is conserved.”

For our new Figure 8/S8, we used MDA-MB-231 cells to confirm our findings, as this cell line expressed high levels of CRACR2A (described in the text on page 8/9:

“CRACR2A was also required to support migration of MDA-MB-231 breast cancer cells (which lack Rab25 expression, but express high levels of CRACR2A) in 3D-CDM (Figure 8C, S8C).”

2) The authors state that BioID-Rabs are expressed at a "level close to endogenous". This should be quantified. Also, authors should clearly show that BioID-Rabs co-localize with the endogenous Rabs. So, immunofluorescence labelling of endogenous Rabs and markers for Early Endosomes (e.g. Rab5, EEA1, etc) and Recycling Endosomes should be performed.

As indicated for reviewer 1 and 2: We compared the level of BirA-Rab11a to Rab11 endogenous levels because of the availability of antibodies (we are not aware of reliable antibodies for Rab4). Also, Rab25 is not usually expressed in the cells used here. We used BirA-Rab11 expression level to approximate a low level expression of BirA-Rab4a and BirA-Rab25, reasoning that similar expression levels allow for more meaningful comparison.

The text now reads:
‘……we adopted a system to select for low level expression of BioID-Rab4a, BioID-Rab11a, and BioID-Rab25 (alongside a cytoplasmic BioID control) at a level close to endogenous Rab11 protein (Figure S1A-C).’

© 2023. Published by The Company of Biologists under the terms of the Creative Commons Attribution License (https://creativecommons.org/licenses/by/4.0/).

As noted for Reviewer 1 and 2, we performed new experiments where we allow uptake of transferrin for 10 minutes (to label early endosomes) or 30 minutes (to label recycling endosomes). In these experiments we see good overlap between internalised transferrin and BioID-Rab4 at 10 minutes, and with BioID-Rab11a and -Rab25 at 30 minutes, indicating that the modified Rabs localize to the appropriate compartments despite the modest overexpression. We also include quantification of Rab11 endo/exo expression levels in Figure 1 for reviewers at the end of this document.

3) In the dot-plot of the high-confidence proximal analysis, the average intensity (represented in the circle colour) should be normalized by the abundance of protein.

This was indeed the case and we have made this more clear in legend- apologies for this lack of clarity.

4) The knock sideways experiments validated high affinity prey interactions, including of sorting nexins with Rab4/11/25. SNX1 and SNX3 showed that they would only significantly redistribute in FKBP-GFP-Rab11a and FKBP-GFP-Rab25, respectively. Authors should comment on why the role of SNX1 and SNX3 was not assessed in migration studies.

We would have liked to perform these experiments but lacked time. These will be the focus of our ongoing studies though.

5) Knock sideways showed that Rab4 was unable to induce significant re-localization of CLINT1. This would suggest that CLINT1 would be a candidate less robust than others identified by BioID and validated by knock sideways experiments. Why did the authors decide to proceed to assess the role of CLINT1 in migration studies?

We felt that the strong indication of close interaction given by biotinylation data, plus the fact that co-localization was evident gave us sufficient reason to follow up CLINT1.

6) Although the authors reported a lack of significant re-localization of CLINT1 by Rab4a, they state that "CLINT1 plays a role in Rab4 (but not Rab25) dependent migration in 3D-CDM". Can the authors comment on this?

We feel the data in Figure S7A demonstrate that knockdown of CLINT1 slows the migration of cells that lack Rab25 expression, but have no effect on the speed of migration when Rab25 is over-expressed. We know from previous work that Rab25 overexpression changes the migration/invasion phenotype of the A2780 cells used here, such that extend longer pseudopodial processes as they move (Caswe 2007, Dozynkiewicz 2012). However, for BT20 cells (which express endogenous levels of Rab25) CLINt1 knockdown does decrease speed of migration in 3D-CDM. We discuss this on page 11 as follows:

“This confirms that the importance of each is generalisable to motility of other cell types, but the reliance of BT20 cells on CLINT1 suggests there could be differences in the way that the two cell types tested here rely on trafficking pathways to migrate. Enodgenous Rab25 is expressed in BT20 cells, albeit at a lower level than in the A2780-Rab25 line where overexpression levels reflect that found in aggressive ovarian cancer. The difference in migration could therefore reflect expression level, and it is interesting to speculate that high expression of Rab25 could overcome the requirement for the Rab4 pathway in motility (as is the case when Rab11-RCP trafficking is activated (Caswell et al., 2008; Christoforides et al., 2012)).”

7) "CLINT1 was identified as a Rab4, -11 and -25 proximal protein (Figure 2)". The study would benefit from additional evidence showing that CLINT1 does not act downstream of Rab11 to control migration of A2780 cells.

We have clarified in the text that the level of direct biotinylation (shown in S3D) and the level of detected CLINT1 (shown in Figure 3) is greater in Rab4a samples than Rab11a or Rab25. We are able to show that CLINT1 is not required for Rab25-driven migration in A2780 cells (Figure 7).

© 2023. Published by The Company of Biologists under the terms of the Creative Commons Attribution License (https://creativecommons.org/licenses/by/4.0/).

8) Authors should include immunofluorescence studies to better characterise the role of Rab4a, Rab11a and Rab25 networks in migration, adhesion and leading-edge related processes. Focal adhesions should be quantified, and actin cytoskeleton described. Such studies should be coupled to the cell migration studies. These would validate and support the conclusions drawn from the GO analysis.

This is a great suggestion- for Rab4 there is published data to support the involvement of Rab4 in adhesion formation (Roberts et al. Curr Biol. 2011; Gu et al JCB 2011), and we cite this. We also performed new experiments (new Figure 2,S2D and S2E) using knockdown of Rab4/Rab11 and expression of Rab25 to analyze differences the actin cytoskeleton and paxillin-positive adhesion formation to support the gene ontology analysis. We therefore added the following text on page 4:

"Rab4 is required for focal adhesion and lamellipodia formation, Rab11 and Rab25 promote filopodia

We and others have shown that Rab4 and Rab11 families control integrin recycling and cell migration (Moreno-Layseca et al., 2019; Paul et al., 2015a), and we further demonstrated that Rab11-RCP recycling can be induced to promote formation of filopodia in cells moving in 3D matrices (Jacquemet et al., 2013; Paul et al., 2015b). Interestingly, knockdown of Rab4a and Rab4b led to a decrease in the number of paxillin positive focal adhesions formed by cells in 2D, whereas Rab11 knockdown or Rab25 overexpression had no effect (Figure S2D-E). Given that gene ontological analysis also pointed towards cytoskeletal regulation, we analysed the morphology of F-actin within protrusions formed by cells moving within 3D-cell derived matrix (CDM). A2780 cells moving in 3D-CDM extend several protrusions, and these protrusions are often tipped by small lamellipodia, characterised by wider veils of F-actin at the leading edge, and/or by narrow finger-like filopodia (Figure 2A). We therefore measured the width of the most advanced protrusion, and scored for presence of lamellipodia and filopodia bearing protrusion, or filopodia alone (lamellipodia alone were not observed). Knockdown of Rab4a and Rab4b led to decrease in protrusion width, and an increase the number of cells bearing protrusions tipped solely by filopodia (Figure 2A-C). Conversely knockdown of Rab11a and Rab11b had no effect on protrusion width, however significantly fewer cells bore filopodial protrusions. (Figure 2A-C). Overexpression of Rab25 (at levels close to those observed in aggressive ovarian cancers (Caswell et al., 2007; Cheng et al., 2004)) also significantly reduced the width of protrusions extended from cells moving in 3D-CDM, and most of these protrusions were tipped by filopodia alone (Figure 2A-C). These data reinforce the notion that Rab4 and Rab11 pathways (and/or the cargoes they carry) are antagonistic, and demonstrate that Rab4 supports formation of wide lamellipodial protrusions, whereas the Rab11 family promotes filopodial protrusion."

9) In the discussion, the authors mention two other papers in which "proximity labelling methods have proven an excellent tool for identification of protein complexes, including for Rab4 and Rab11". The authors should also discuss if there are overlapping results.
We have expanded our discussion of these as follows:

"Our data show good overlap with these studies but differs primarily in the direct comparison between Rab4a, Rab11a and Rab25 analysed using quantitative label free proteomics to generate a statistically robust interaction network linking these three recycling regulators. Interestingly, CLINT1 was identified as a Rab4-proximal protein in other datasets (Go et al., 2021; Del Olmo et al., 2019), ESCPE-1 and retromer complex members have been identified with Rab11 and Rab4 baits (Gillingham et al., 2019; Go et al., 2021; Del Olmo et al., 2019), and the CRACR2A:Rab11a proximity was also found by Go et al. Our study furthers this complementarity by using directly biotinylated peptides…."

Minor comments:

• Figure 1: Panel A is too small. Insets are hard to interpret. The size of the whole panel should be increased.

This has been fixed.

© 2023. Published by The Company of Biologists under the terms of the Creative Commons Attribution License (https://creativecommons.org/licenses/by/4.0/).

• Description of results regarding the trafficking machinery associated with Rab4a, Rab11a and Rab25 does not follow the same organization and structure as in Figure 2. The authors should try to match the organization of data and its description to improve readability.

We have improved this.

• In Figure 4B and S4C there are two labels for 1 and 2.

We thank the reviewer for pointing out our mistake, this has been fixed.

• Figure S4E merge of GFP-FKBP Rab11a cells shows poor overlap. A replacement should be considered.

We increased the contrast of this and the control images to better show this in Figure S5E.

• There are several typos in the discussion and in Figure 7 ("CRACRA" should be CRACR2A)

We thank the reviewer for identifying this error and have carefully proofread our manuscript.

Reviewer #3 (Significance (Required)):
• The manuscript presents an approach that allow the identification of Rab-associated networks and the direct comparison between GTPases. This is of relevance since we still lack robust methodologies to identify the endosomal trafficking machinery underlying migration in cancer cells. By not targeting Rab4 specific machinery (e.g. TBC1D5), the authors missed the opportunity to expand the knowledge regarding the machinery sustaining Rab4-dependent migration in cancer cells.

We have explained our choice of follow up candidates more clearly. TBC1D5 knockdown could not be completed in time for a revised version unfortunately but we do aim to follow this up.

• The work targets an audience interested in endosomal trafficking and protein recycling in cancer cell migration.

• The reviewer is a translational cancer biologist with expertise in cytoskeleton, endosomal recycling, signaling and cancer.

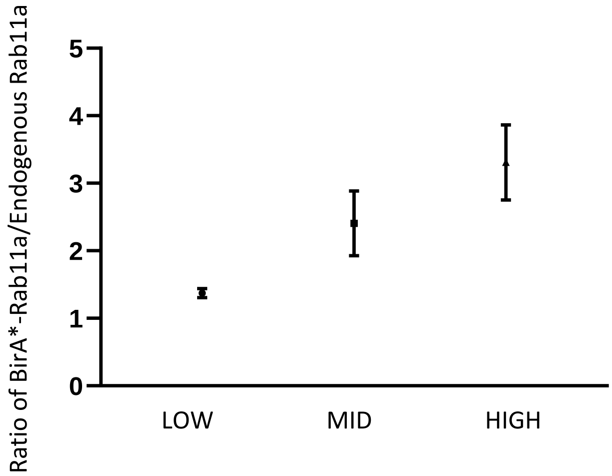

Figure 1 for reviewers: Expression of myc-BirA*-Rab11a in stable cell lines and endogenous Rab11 was quantified by western blotting in cells sorted for low, mid or high myc-BirA*-Rab11 levels. Graph shows the ratio of expressed fusion protein to endogenous Rab11a from two independent experiments (error bars=SD).

© 2023. Published by The Company of Biologists under the terms of the Creative Commons Attribution License (https://creativecommons.org/licenses/by/4.0/).

Second decision letter

MS ID#: JOCES/2022/260468

MS TITLE: Proximity labelling identifies pro-migratory endocytic recycling cargo and machinery of the Rab4 and Rab11 families

AUTHORS: Beverley Wilson, Chloe Flett, Jakub Gemperle, Craig Lawless, Matthew Hartshorn, Eleanor Hinde, Tess Harrison, Megan Chastney, Sarah Taylor, Jennifer Allen, Jim C Norman, Thomas T Zacharchenko, and Patrick Caswell
ARTICLE TYPE: Tools and Resources

I am happy to tell you that your manuscript has been accepted for publication in Journal of Cell Science, pending standard ethics checks.

© 2023. Published by The Company of Biologists under the terms of the Creative Commons Attribution License (https://creativecommons.org/licenses/by/4.0/).
